# Discovery of 2-(4-Acrylamidophenyl)-Quinoline-4-Carboxylic Acid Derivatives as Potent SIRT3 Inhibitors

**DOI:** 10.3389/fchem.2022.880067

**Published:** 2022-03-30

**Authors:** Qian Hui, Xueming Li, Wenli Fan, Congying Gao, Lin Zhang, Hongyu Qin, Liuya Wei, Lei Zhang

**Affiliations:** ^1^ Department of Medicinal Chemistry, School of Pharmacy, Weifang Medical University, Weifang, China; ^2^ Department of Inorganic Chemistry, School of Pharmacy, Weifang Medical University, Weifang, China

**Keywords:** SIRT3, selective inhibitor, mixed-lineage leukemias, cell cycle arrest, differentiation

## Abstract

In discovery of novel SIRT3 inhibitors for the treatment of cancer, a series of 2-(4-acrylamidophenyl)-quinoline-4-carboxylic acid derivatives were designed and synthesized. Among the derived compounds, molecule P6 exhibited SIRT3 inhibitory selectivity with IC_50_ value of 7.2 µM over SIRT1 (32.6 µM) and SIRT2 (33.5 µM). molecular docking analysis revealed a specific binding pattern of P6 in the active site of SIRT3 compared with the bindings in the active site of SIRT1 and SIRT2. In the antiproliferative and colony forming assay, molecule P6 showed potent inhibitory activity against a group of MLLr leukemic cell lines. Further analysis revealed that induction of G0/G1 phase cell cycle arrest and cell differentiation, but not apoptosis, makes contributions to the anticancer effects of P6. Collectively, a potent SIRT3 inhibitor (P6) was discovered as a lead compound for the leukemic differentiation therapy.

## Introduction

Sirtuins (SIRTs) are a group of nicotinamide adenine dinucleotide (NAD^+^) dependent deacetylases (HDACs) belonging to the class III HDAC family (Zhao et al., 2004; Schwer and Verdin, 2008). SIRTs are divided into four subclasses according to the homology of the conserved catalytic core domain. Class I is comprised of SIRT1, 2, and 3, which exhibit robust deacetylase activity. SIRT4 and SIRT5 locating in mitochondria are classified into class II and class III, respectively. SIRT6 and 7 primarily found in the nucleus are assigned to Class IV. With various enzymatic activities, SIRTs play an important role in the regulation of a wide range of intracellular processes including metabolism, longevity, aging, response to stress and especially cancer (Yamamoto et al., 2007; Hirschey, 2011).

Class I SIRTs has been extensively studied in the pathogenesis of various diseases compared with other SIRT isoforms (Anderson et al., 2014). Activation or inhibition of SIRT1-3 have been considered as potential therapeutic strategies for the treatment cancer and neurodegenerative disorders (Lee, 2019). Compared with SIRT1 (located in the nucleus) and SIRT2 (located in the cytosol), the SIRT3 isoform is the only mitochondrially localized SIRT that exhibits potent deacetylase activity (Michishita et al., 2005). While the other two isoforms of mitochondrial sirtuins, SIRT4 and SIRT5, have less deacetylase activity (Verdin et al., 2004). Therefore, SIRT3 is a key mitochondrial deacetylase which can be specifically targeted for the development of therapeutic drugs.

In different types of cancers, SIRT3 has dichotomous role in cancer process as a tumor promoter or suppressor (Chen et al., 2014). It is reported that overexpression of SIRT3 can reprogram mitochondrial metabolism by enhancement of oxidative phosphorylation (OxPhos) and decrease of ROS generation in acute myeloid leukemia (AML) cells (Ma et al., 2019). As a result, AML cells were protected from chemotherapy and Ara-C-induced apoptosis. SIRT3 overexpressed in chronic lymphocytic leukemia (CLL) (Maiti et al., 2021) and diffuse large B cell lymphomas (DLBCLs) (Li et al., 2019) cells contributes to the proliferation, survival and self-renewal of tumor cells. SIRT3 overexpression was also revealed to promote survival and tumorigenesis in oral squamous cell carcinoma (OSCC) (Alhazzazi et al., 2011a). Therefore, SIRT3 is a potential therapeutic target for the treatment of cancer. Selective inhibition of SIRT3 is promising in the development of anticancer drug by targeting a specific type of cancer with abnormal SIRT3 functions such AML and OSCC.

**FIGURE 1:**
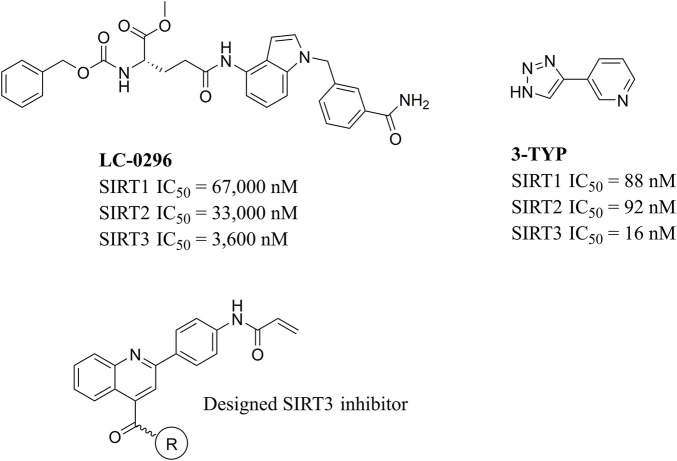
Structure of LC-0296, 3-TYP and the designed SIRT3 inhibitors in the current study.

Due to the high similarity of mechanism and active site residues between SIRT1-3, it is difficult to design SIRT3 selective inhibitors (Alhazzazi et al., 2011b). Although a lot of molecules exhibited SIRT3 inhibitory potency, the SIRT3 selective inhibition strategy has been rarely reported (Troelsen et al., 2021). To the best of our knowledge, only two compounds, LC-0296 (Alhazzazi et al., 2016) and 3-TYP (Galli et al., 2012), were elucidated as high SIRT3 selective inhibitors and showed selectivity for SIRT3 over SIRT1 and SIRT2 (Figure 1). In discovery of potent SIRT3 inhibitors for the treatment of cancer, a series of compounds were designed and synthesized for the activity screening. Considering that bulky groups with aromatic rings are usually presented in the structures of SIRT3 inhibitors (Zhang et al., 2020), 4-acrylamidophenyl-quinoline group was introduced to the designed SIRT3 inhibitors in the present study. Substituted groups were introduced to the 4-carboxylic group in the quinoline ring. The derived compounds were investigated in the enzyme inhibitory screening, molecular docking analysis, *in vitro* antiproliferative assay, colony formation test, cell cycle, apoptotic and cell differentiation studies.

## Chemistry

**SCHEME 1:**
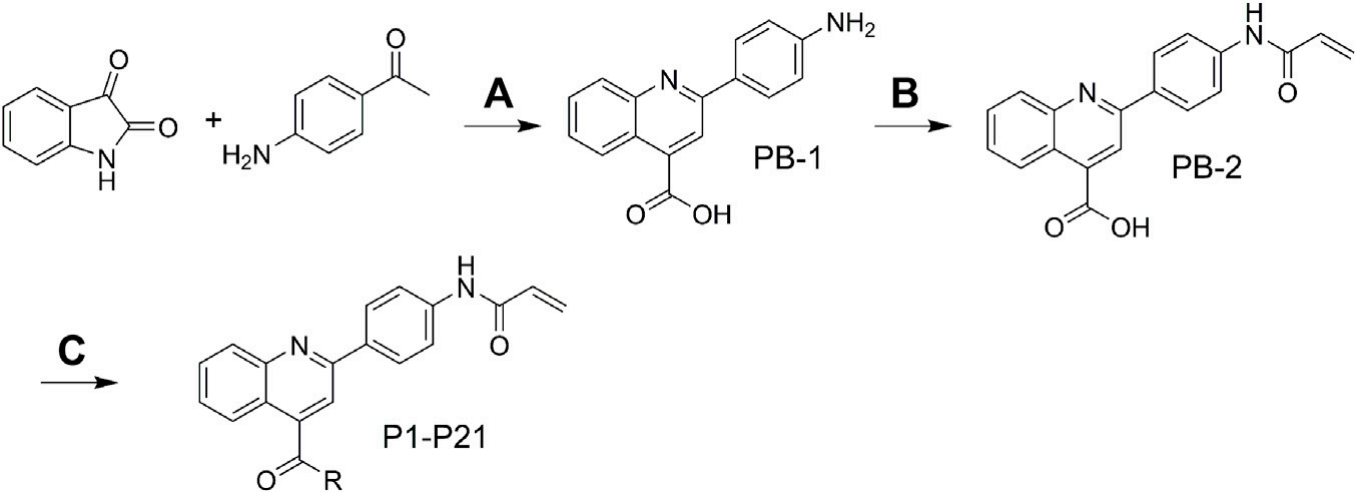
**(A)** 33% KOH, EA, 85°C; **(B)** THF, H_2_O, NaHCO_3_; **(C)** DCM, Et_3_N, TBTU.

The target molecules were synthesized as illustrated in Scheme 1. The starting material isatin and 1-(4-aminophenyl)ethan-1-one were heated under alkaline condition to afford intermediate PB-1. The followed coupling of acryloyl chloride to PB-1 was performed to synthesize intermediate PB-2. At last, various phenylamines and substituted phenylpiperazines were introduced to the carboxylic group in the quinoline. The structures of target molecules (P1-P21) were confirmed by high-resolution mass, ^1^H NMR and ^13^C NMR spectra.

## Results and Discussion

### SIRT3 Inhibitory Activity

The synthesized molecules were firstly screened in the enzyme inhibitory assay. Percentage inhibitory rate was used to evaluate the SIRT3 inhibitory activity of the derived compounds at concentration of 10 µM (Table 1). Nicotinamide was utilized as positive control, and showed IC_50_ value of 39.1 µM in the enzymatic inhibition test. The SIRT3 inhibitory result revealed that molecule P6 and P19 have good inhibitory potency with inhibitory rate of 65.15 and 55.26 compared with other compounds. Among P1-P9, ortho-substitution in the phenyl group exhibited improved inhibitory potency, such as molecule P6 and P8. Substitutions with increased size in the phenyl ring might improve the inhibitory potency in the rest compounds (P10-P21), such as P15, P19, and P20.

**TABLE 1:** Structure, SIRT3 inhibitory and antiproliferative activity of the derived compounds.

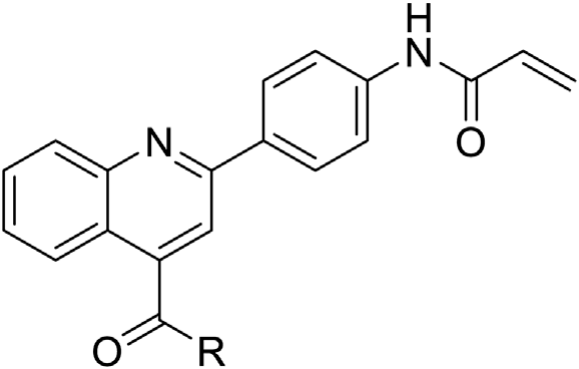			
**Copounds**	**R**	**SIRT3^a^**	**THP-1^b^**
P1	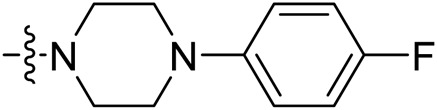	20.21 ± 1.22	32.48 ± 2.56
P2	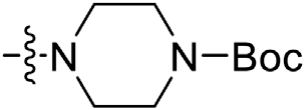	23.52 ± 2.13	26.37 ± 1.55
P3	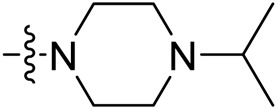	30.62 ± 2.45	8.78 ± 0.37
P4	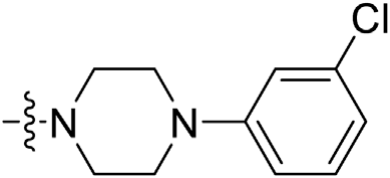	21.77 ± 1.62	57.93 ± 3.04
P5	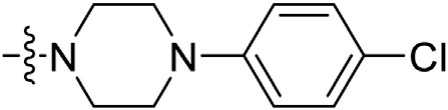	22.63 ± 1.87	55.96 ± 2.66
P6	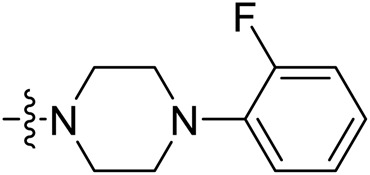	65.15 ± 3.26	85.90 ± 3.42
P7	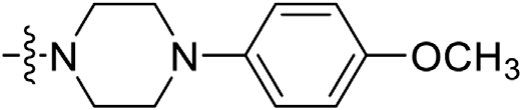	24.85 ± 1.47	12.43 ± 1.07
P8	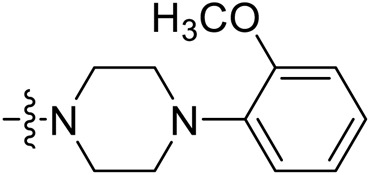	45.54 ± 3.67	73.66 ± 3.74
P9	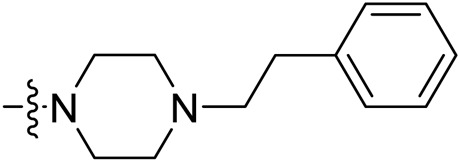	30.22 ± 1.94	11.71 ± 0.88
P10	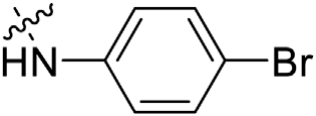	26.76 ± 1.88	44.27 ± 1.96
P11	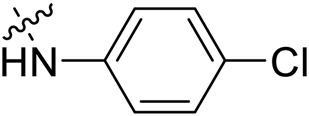	35.71 ± 1.54	57.97 ± 2.08
P12	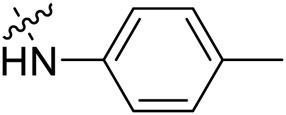	38.91 ± 2.62	72.85 ± 3.25
P13	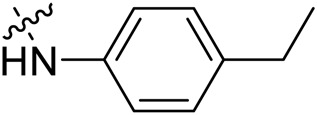	40.25 ± 2.91	59.89 ± 3.27
P14	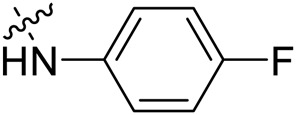	26.43 ± 1.78	53.21 ± 4.26
P15	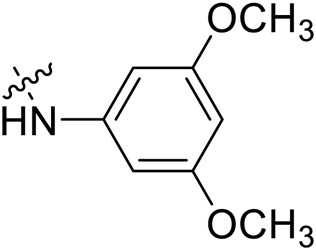	42.76 ± 1.26	49.63 ± 2.52
P16	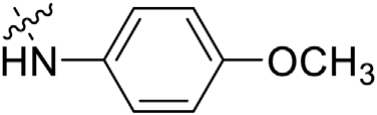	28.38 ± 1.85	13.33 ± 1.23
P17	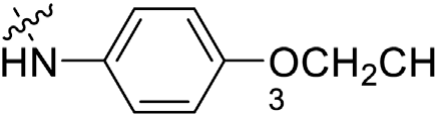	29.55 ± 1.43	13.84 ± 1.01
P18	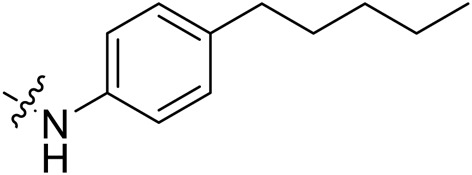	30.62 ± 2.89	29.97 ± 1.39
P19	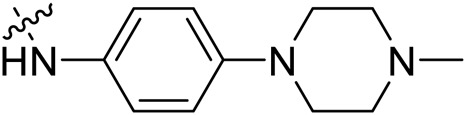	55.26 ± 3.21	55.64 ± 2.24
P20	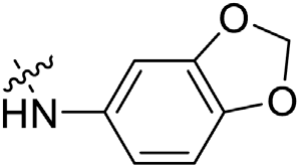	45.13 ± 2.55	28.09 ± 1.76
P21	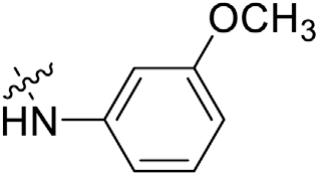	22.22 ± 1.72	35.42 ± 1.58
Nicotinamide		35.58 ± 2.15	ND
Ara-C		ND	80.44 ± 3.22

a Illustrated as percentage inhibitory rate at dose of 10 μM, and each value is the mean of three experiments.

b Illustrated as percentage inhibitory rate at dose of 1 μM, and each value is the mean of three experiments.

ND, not determined.

Enzyme selectivity test was performed to evaluate the inhibitory pattern of active compounds (Table 2). Molecule P6 and P19 with high SIRT3 inhibitory rate were selected for the selectivity assay. The control compound, nicotinamide, did not exhibit selectivity among SIRT1-3 in the test. It is notably that molecule P6 can selectively inhibit SIRT3 with IC_50_ value of 7.2 µM compared with the IC_50_ values against SIRT1 (32.6 µM) and SIRT2 (33.5 µM). Both the activity and selectivity of molecule P19 against SIRT3 were not as high as that of P6. It is suggested that molecule P6 can be used as a SIRT3 selective inhibitor for further analysis.

**TABLE 2:** Enzyme inhibitory selectivity of representative compounds (IC_50_, µM^a^).

Compounds	SIRT1	SIRT2	SIRT3
P6	32.6 ± 0.8	33.5 ± 1.6	7.2 ± 0.5
P19	57.6 ± 2.2	63.6 ± 4.2	27.3 ± 1.7
Nicotinamide	32.1 ± 2.6	24.0 ± 1.9	15.5 ± 1.9

a Each value is the mean of two experiments.

### Binding Pattern Analysis

**FIGURE 2:**
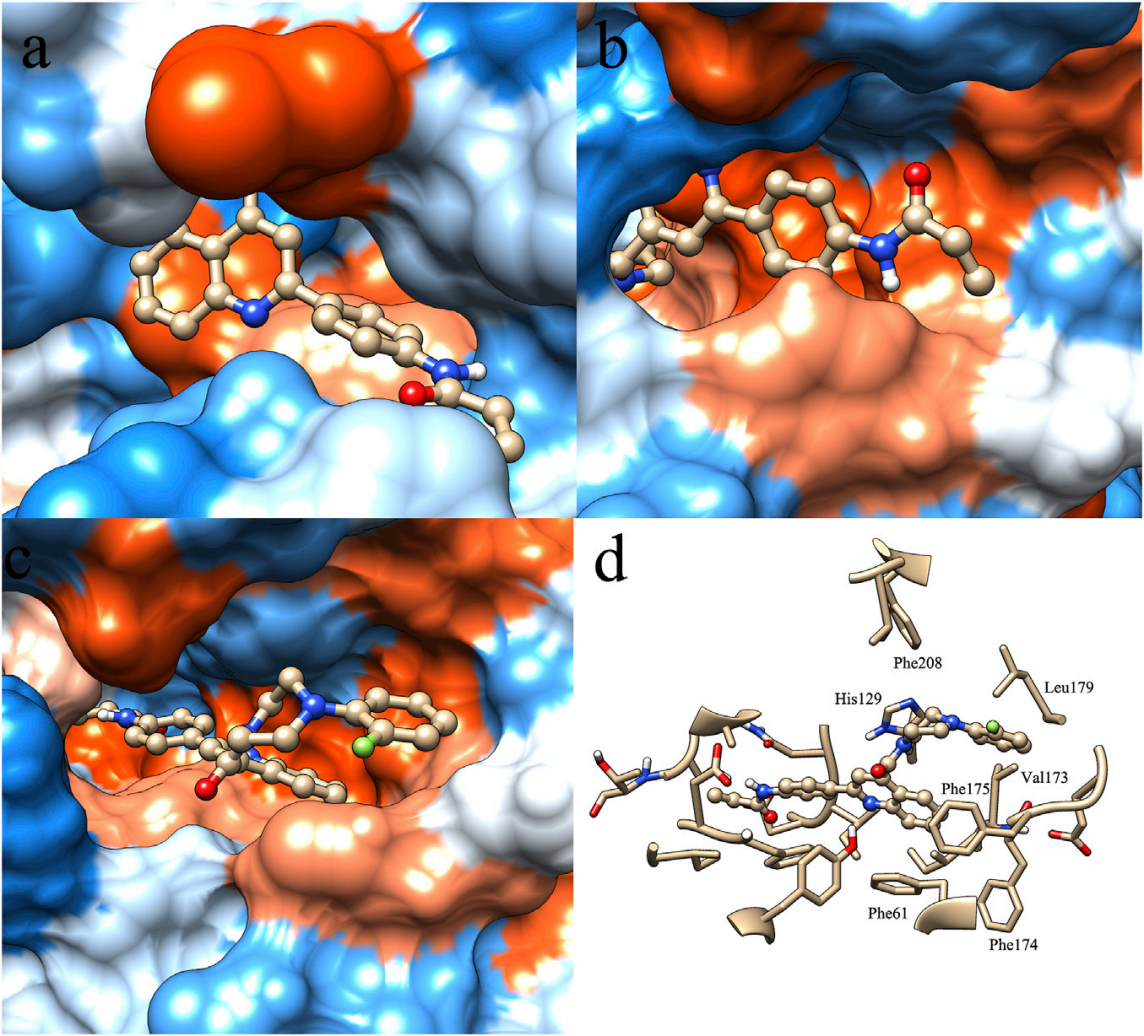
Binding pattern of molecule P6 in the active site of SIRT1 **(A)**, SIRT2 **(B)** and SIRT3 **(C,D)**.

In order to find clues that affect the selectivity of molecule P6, molecular docking was performed based on the structure of SIRT1-3. Compound P6 was docked flexibly to the NAD-binding site of SIRT1-3, and the binding patterns were analyzed (Figure 2). It is significant that the active of SIRT3 (Figure 2C) is characterized with bigger opening and multiple hydrophobic pockets compared with the catalytic site of SIRT1 (Figure 2A) and SIRT2 (Figure 2B). The quinoline and 2-fluorophenyl groups occupied different hydrophobic pockets in the opening of SIRT3 active site. Phe61 and Phe175 were key residues that make contributions to the hydrophobic interactions of SIRT3-P6 complex (Figure 2D). As to SIRT1 and SIRT2, the acrylamide terminal of P6 located in the opening of both active sites. The lack of corresponding hydrophobic interactions was considered to result in the reduced inhibitory potency of P6 against SIRT1 and SIRT2.

### Antiproliferative Assay

**FIGURE 3:**
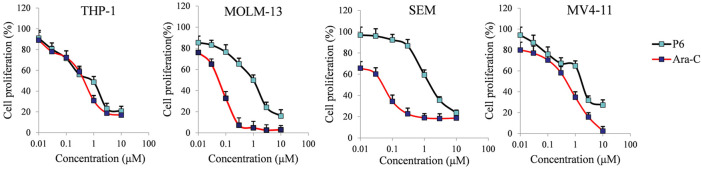
The effect of molecule P6 in inhibition of MLLr cell proliferation. Cells were treated with different concentrations of P6 or Ara-C (0–10 µM) for 72 h and subjected to CCK-8 assay. Error bars represent the mean ± SD.

Mixed-lineage leukemias (MLLs), including acute myeloid leukemia (AML) and acute lymphoblastic leukemia (ALL), are very aggressive hematologic malignancies with unique clinical and biological characteristics (Pigneux et al., 2015). MLLs are often lethal due to the development of resistance and high relapse rates after established treatment (Liang et al., 2017). Therefore, the antiproliferative activities of P6 against several leukemic cell lines with MLL gene rearrangements (MLLr) were investigated in the current study. Cytarabine (Ara-C) clinically used in the treatment AML and ALL was utilized as the positive control in the *in vitro* test against THP-1, MOLM-13, SEM and MV4-11 cell lines. Molecule P6 exhibited the highest inhibitory activity among the derived compounds in the *in vitro* antiproliferative screening using THP-1 cells (Table 1). Therefore, only compound P6 was selected for further anticancer evaluation against the MLLr cell lines. The results showed that molecule P6 could effectively inhibit the growth of THP-1, MOLM-13, SEM and MV4-11 cells with IC_50_ value of 0.87, 0.98, 1.79, and 1.90 µM, respectively (Figure 3), comparing with Ara-C (IC_50_ value of 0.52, 0.06, 0.06, and 0.54 µM, respectively). It is revealed that molecule P6 is potent in inhibition the growth of MLLr leukemic cells.

### Colony Formation Test

**FIGURE 4:**
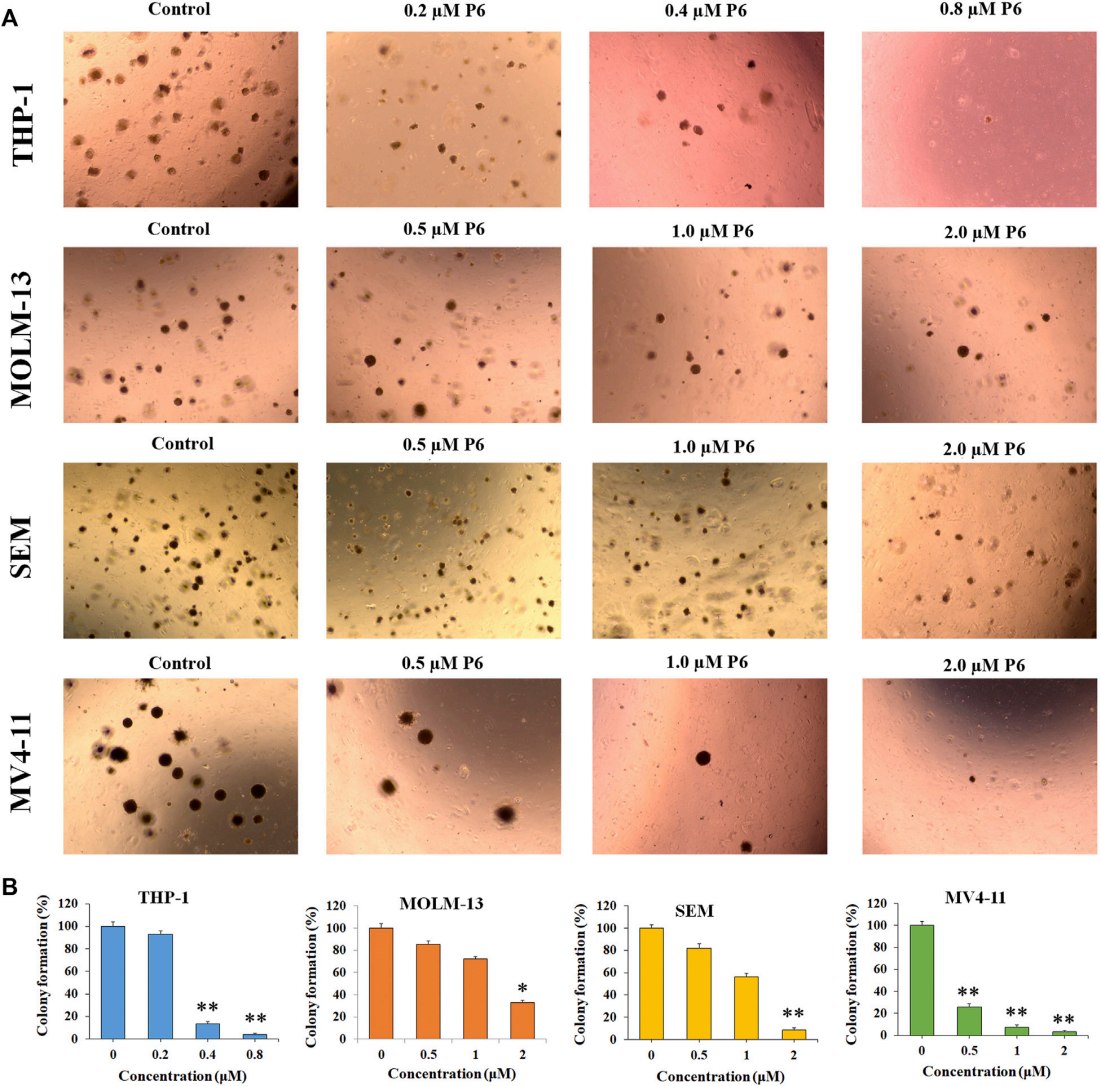
P6 suppresses the colony formation in THP-1, MOLM-13, SEM and MV4-11 cells. **(A)** Cells morphology observed under light microscopy after treated with P6 at the concentrations of 0.2–2 mM for 12 days. **(B)** Graph illustration of the colony number (*p < 0.05, **p < 0.01).

Colony forming assay is an *in vitro* quantitative technique to examine the capability of a single cell to grow into a large colony. In the current study, the effects of compound P6 on MLLr leukemic cells was determined by the colony formation assay. Cells (THP-1, MOLM-13, SEM and MV4-11) were treated with P6 at dose of 0.2–2.0 µM for 12 days and the formation of colonies was observed under a microscope. The result showed that molecule P6 can significantly reduce the number of colonies with a dose dependent manner (Figure 4). Notably, THP-1 cell line was the most vulnerable among the tested cells, and the colony forming ability of THP-1 cells could be inhibited by a low concentration of P6 (colony formation percentage of 93.2, 13.4, and 4.1 at concentration of 0.2, 0.4, and 0.8 µM). The results revealed the anticancer effects of P6 by inhibiting the colony forming ability of the tested MLLr leukemic cell lines.

### Cell Cycle Analysis

**FIGURE 5:**
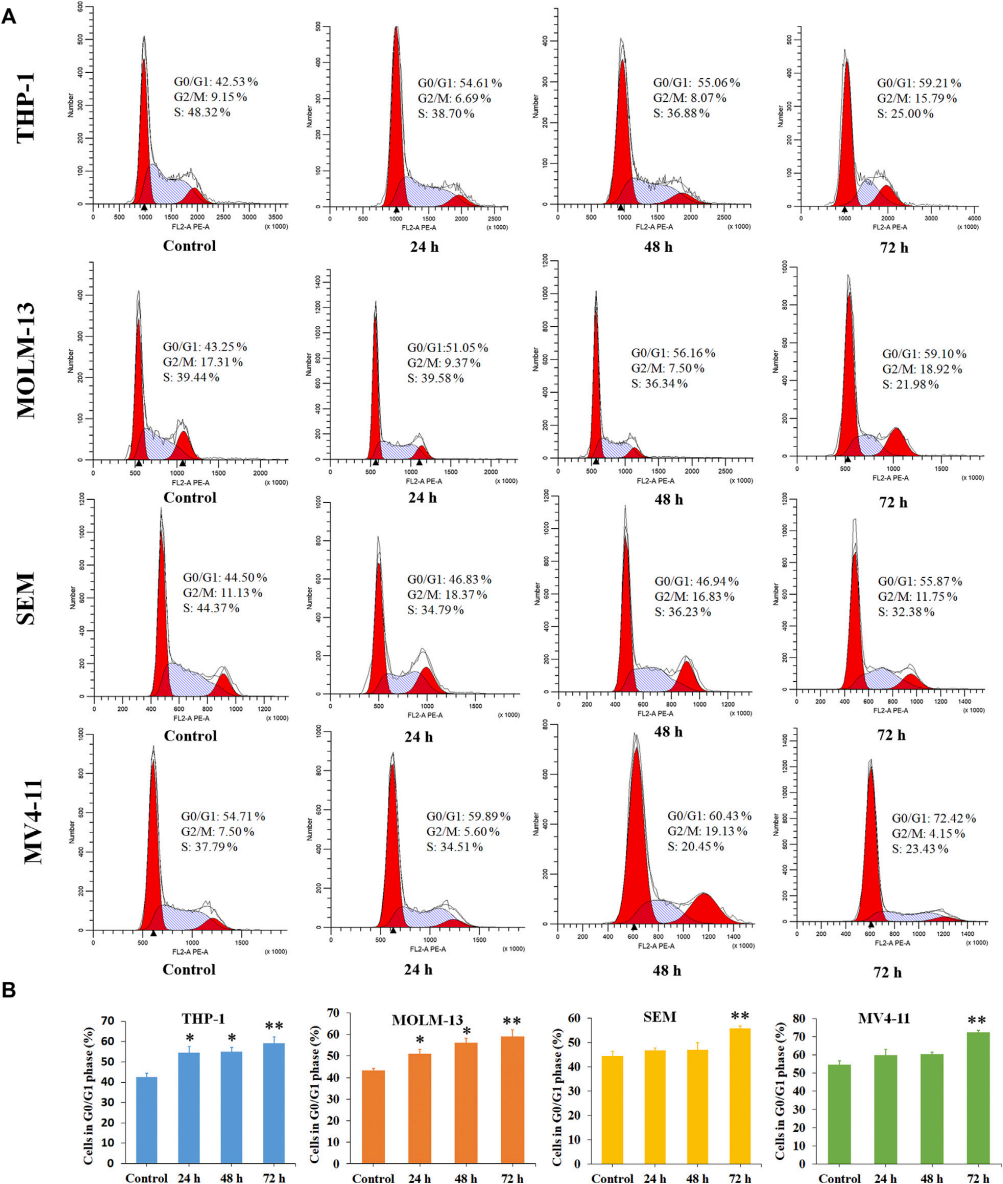
Molecule P6 induces cell cycle arrest in THP-1, MOLM-13, SEM and MV4-11 cells. **(A)** Cells were treated with 1.0 (THP-1, MOLM-13 cells) and 2.0 (SEM and MV4-11 cells) µM of P6 for 72 h, then flow cytometry was used to detect the percentage of cells in various phase of the cell cycle. **(B)** Graph illustration of the cell proportion in G0/G1 phase (*p < 0.05, **p < 0.01).

Cancer is a group of diseases in which cells divide continuously and excessively. Cancer-associated mutations perturb cell cycle control, and allow continuous cell division (Matthews et al., 2021). Cell cycle arrest is usually induced by chemotherapeutic drugs in the cancer treatment. Therefore, in the present study, the effect of P6 on cell cycle progression was evaluated in THP-1, MOLM-13, SEM and MV4-11 cells at dose of 0.9–2.0 µM. As shown in Figure 5, it is significant that the proportion of G0/G1 cells increased with a time dependent manner in the MLLr leukemic cells treated with different doses of P6. It is suggested that molecule P6 is capable of causing MLLr leukemic cell cycle arrest at G0/G1 phase, and the cell cycle arrest inducing role makes contribution to the antiproliferation effects of P6.

### Apoptosis Study

**FIGURE 6:**
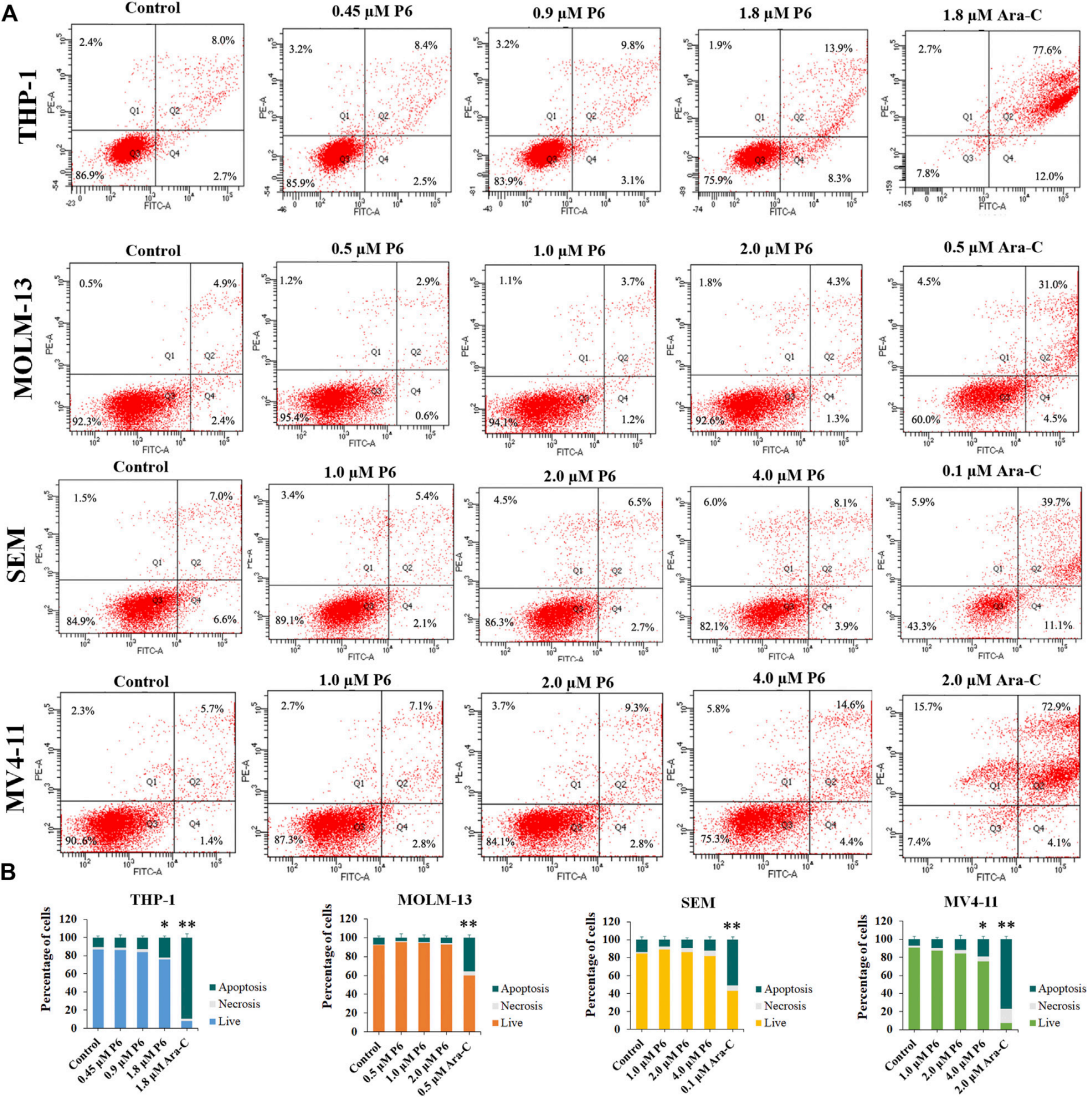
Molecule P6 does not induce significant apoptosis in THP-1, MOLM-13, SEM and MV4-11 cells. **(A)** Cells were treated with P6 or Ara-C for 72 h and cell apoptosis was determined by flow cytometric analysis. **(B)** Graph illustration of the percentage of living cells and cells undergoing necrosis/apoptosis (*p < 0.05, **p < 0.01).

Dysregulation of apoptosis is involved in tumor initiation, progression and metastasis (Carneiro and El-Deiry, 2020). Chemotherapeutic drugs have a common intent to activate apoptosis in cancer cells. To evaluated whether apoptosis is associated with the antiproliferation effects of P6, THP-1, MOLM-13, SEM, and MV4-11 cells were treated with different concentrations of P6 or Ara-C for 72 h and cell apoptosis was determined by flow cytometric analysis. The result showed no significant apoptosis of the tested MLLr leukemic cells after treatment with various doses of P6 (Figure 6). In contrast, the positive control Ara-C, led to obvious apoptosis in the tested cell lines at indicated concentrations, especially in THP-1 and MV4-11 cells. It is indicated that apoptosis is not involved in the anticancer effects of P6 due to the minimal signs of apoptosis in MLLr cell lines.

### Cell differentiation Test

**FIGURE 7:**
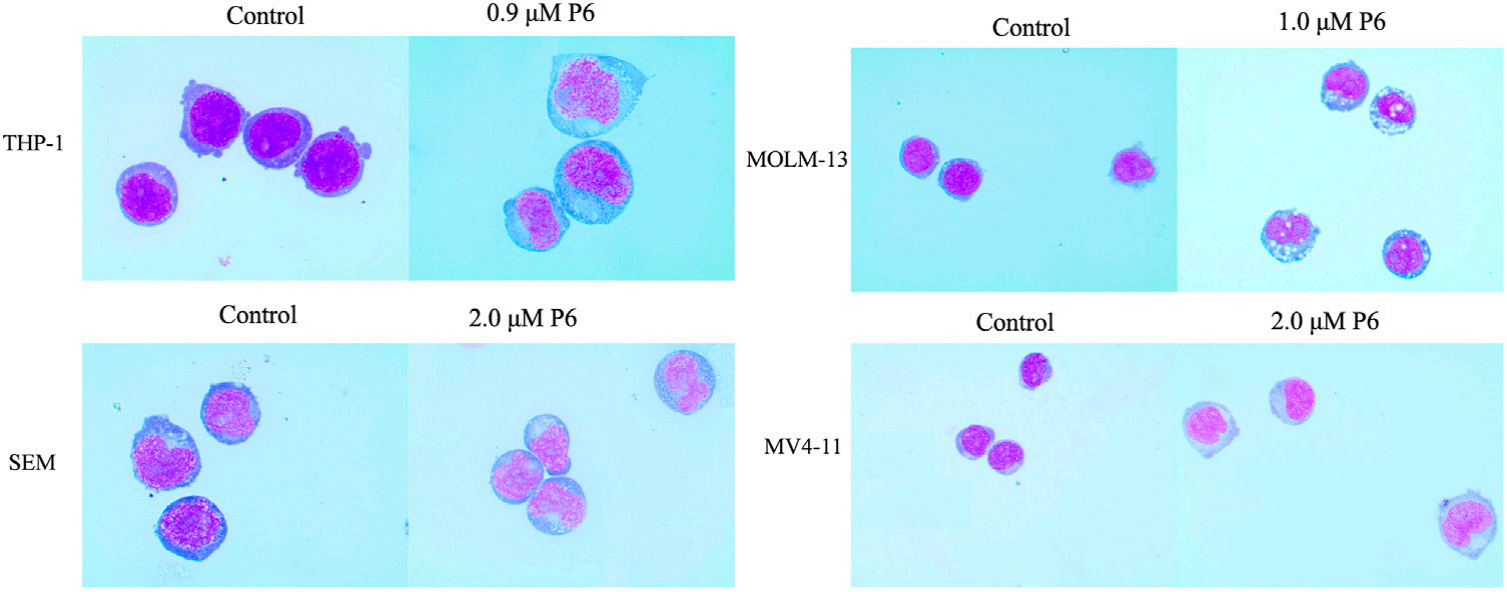
Wright-Giemsa staining images of cells captured by oil immersion lens (×1,000).

Since apoptosis did not play a role in the antiproliferative effect of P6, morphology and flow cytometry analysis were performed to assess the differentiation of MLLr leukemic cells treated with molecule P6. It was observed that all the four cell lines showed increased cell size with a decrease in the nuclear-cytoplasmic ratio (Figure 7). It is indicated that molecule P6 induced the cell differentiation accompanied with morphological changes. Differentiation therapy holds great promise for cancer treatment with improved safety properties comparing with conventional cancer treatments (Yan and Liu, 2016). In the present analysis, compound P6 exhibited potential of inducing MLLr cells to normal cells by elimination of tumor phenotypes. Therefore, the SIRT3 selective inhibitor P6 could be utilized as a lead compound in the MLLr leukemic differentiation therapy.

## Conclusion

SIRT3, a key mitochondrial deacetylase, has been revealed to be a potential therapeutic target for the treatment of leukemia. In the current study, a series of 4-acrylamidophenyl-quinoline containing compounds were designed and synthesized. The SIRT3 enzyme inhibitory results showed that the synthesized molecules could block the deacetylation activity of SIRT3. In the enzyme inhibition selectivity assay, molecule P6 exhibited SIRT3 inhibitory selectivity over SIRT1 and SIRT2. Molecular docking study predicted a specific binding pattern of P6 in the active site of SIRT3 comparing with the bindings of P6 in the active sites of SIRT1 and SIRT2. In the *in vitro* antiproliferative test, the SIRT3 selective inhibitor P6 showed potent anticancer activity against a group of MLLr leukemic cell lines (THP-1, MOLM-13, SEM, and MV4-11) compared with the positive control Ara-C. Molecule P6 also exhibited potency in inhibiting the colony forming ability of all the tested MLLr leukemic cell lines in the colony formation assay. Moreover, G0/G1 phase cell cycle arrest played a role in the anticancer effects of P6 on the tested MLLr cells. Apoptotic analysis revealed that molecule P6 can not induce apoptosis of the MLLr leukemic cell lines. It is suggested that apoptosis is not involved in the antiproliferation of P6. In the cell differentiation test, molecule P6 promoted MLLr leukemic cell differentiation with increased cell size and decreased nuclear-cytoplasmic ratio. In conclusion, a potent SIRT3 selective inhibitor (P6) was discovered with *in vitro* MLLr leukemia inhibitory activity. The findings in the present study also revealed the potential of SIRT3 selective inhibitors in the MLLr leukemic differentiation therapy.

## Materials and Methods

All chemicals were obtained from commercial suppliers and can be used without further refinement. All reactions were detected by TLC using 0.25 mm silica gel plate (60GF-254). UV light and ferric chloride were used to show TLC spots. ^1^H NMR and ^13^C NMR spectra were recorded on a Bruker DRX spectrometer at 500 MHz, using TMS as an internal standard. High-resolution mass spectra was performed in Weifang Medical University.

And 1-(4-aminophenyl)ethan-1-one were heated under alkaline condition to afford intermediate PB-1. The followed coupling of acryloyl chloride to PB-1 was performed to synthesize intermediate PB-2. At last, various phenylamines and substituted phenylpiperazines were introduced to the carboxylic group in the quinoline. The structures of target molecules (P1-P21) were confirmed by high-resolution mass, ^1^H NMR and ^13^C NMR spectras (Supplementary Material).

### 2-(4-Aminophenyl)Quinoline-4-Carboxylic Acid (PB-1)

The starting material isatin (0.5 g, 3.4 mmol) was dissolved in 33% KOH (10 ml). Then, the 1-(4-aminophenyl)ethan-1-one (0.50 g, 3.75 mmol) ethanol solution (20 ml) was added. The mixture was heated at 85°C for 5 h in an oil bath. Then, the reaction solution was evaporated in vacuum, diluted with 100 ml of H_2_O, and adjusted to PH 3 ∼ 4 with 3 mol/L HCl. The derived brownish red solid was filtered and washed with EtOAc to obtain PB-1 (0.60 g, yield of 54%). HRMS C_16_H_12_N_2_O_2_ [M + H]^+^ calc. 265.0320, found 265.0960. ^1^H NMR (400 MHz, DMSO) δ 8.58 (d, J = 8.3 Hz, 1H), 8.32 (s, 1H), 8.05 (d, J = 8.1 Hz, 3H), 7.77 (t, J = 7.3 Hz, 1H), 7.59 (t, J = 7.4 Hz, 1H), 6.72 (d, J = 8.1 Hz, 2H).

### 2-(4-Acrylamidophenyl)Quinoline-4-Carboxylic Acid (PB-2)

To a solution of PB-1 (0.5 g, 1.9 mmol) in THF, NaHCO_3_ (0.24 g, 2.8 mmol) was added, and acryloyl chloride (0.2 g, 2.27 mmol) was added dropwise. Then, the mixture was stirred for 4 h. Then, the reaction solution was evaporated in vacuum, diluted with H_2_O. Orange solid was derived and washed with EtOAc to afford PB-2 (0.48 g, yield of 80%). HRMS C_19_H_14_N_2_O_3_ [M + H]^+^ calc. 319.1038, found 319.1066. ^1^H NMR (400 MHz, DMSO) δ 10.44 (s, 1H), 8.63 (d, J = 8.4 Hz, 1H), 8.30 (d, J = 2.4 Hz, 2H), 8.27 (s, 1H), 8.08 (d, J = 8.4 Hz, 1H), 7.89 (d, J = 8.4 Hz, 2H), 7.82 (d, J = 11.2 Hz, 1H), 7.77 (d, J = 7.7 Hz, 1H), 7.60 (t, J = 7.6 Hz, 1H), 6.51 (dd, J = 16.9, 10.0 Hz, 1H), 6.31 (d, J = 16.9 Hz, 1H), 5.81 (d, J = 10.3 Hz, 1H).

To the solution of PB-2 (0.50 g, 1.57 mmol) in DCM, Et_3_N (0.18 g, 1.73 mmol) and TBTU (0.56 g, 1.73 mmol) was added at 0°C. The solution was kept at 0°C for 20 min, 1-(4-fluorophenyl)piperazine Dihydrochloride (0.44 g, 1.73 mmol) was added. The reaction mixture was stirred at room temperature for overnight. Then, the solvent was evaporated under vacuum. The concentrate is dissolved in EtOAc (50 ml), washed with saturated citric acid (3 × 20 ml), NaHCO_3_ (3 × 20 ml), and NaCl (3 × 20 ml), and then dried with MgSO_4_. Target compound P1 (0.54 g, yield of 72%) was derived by crystallization in EtOAc as white powder. HRMS C_29_H_25_FN_4_O_2_ [M + H]^+^ calc. 481.54074, found 481.19843. ^1^H NMR (400 MHz, DMSO) δ 10.39 (s, 1H), 8.34 (d, *J* = 8.7 Hz, 2H), 8.18–8.10 (m, 2H), 7.90–7.80 (m, 4H), 7.64 (t, *J* = 7.6 Hz, 1H), 7.06 (t, *J* = 8.8 Hz, 2H), 6.97 (dd, *J* = 9.1, 4.6 Hz, 2H), 6.49 (dd, *J* = 16.9, 10.1 Hz, 1H), 6.35–6.27 (m, 1H), 5.85–5.76 (m, 1H), 4.03 (dd, *J* = 14.1, 7.1 Hz, 2H), 3.91 (d, *J* = 4.9 Hz, 1H), 3.31–3.22 (m, 3H), 3.09 (s, 1H), 2.91 (s, 1H). ^13^C NMR (400 MHz, DMSO) δ 166.25, 163.83, 155.84, 148.09, 143.72, 141.22, 133.47, 132.22, 130.98, 130.04, 128.45, 127.76, 125.18, 123.17, 119.82, 118.33, 115.95, 115.70, 49.97, 49.66, 46.92, 41.55, 14.56.

### Derivatives P2-P21 Were Prepared as Described for P1

4-[2-(4-Acryloylamino-phenyl)-quinoline-4-carbonyl]-piperazine-1-carboxylic acid tert-butyl ester (P2) Derived by crystallization in EtOAc as white powder (0.20 g, yield of 70%). HRMS C_28_H_3_0N_4_O_4_ [M + H]^+^ calc. 487.23006, found 487.22934. ^1^H NMR (400 MHz, DMSO) δ 10.39 (s, 1H), 8.32 (d, *J* = 8.7 Hz, 2H), 8.11 (d, *J* = 9.3 Hz, 2H), 7.87 (t, *J* = 6.5 Hz, 2H), 7.84–7.78 (m, 2H), 7.62 (t, *J* = 7.5 Hz, 1H), 6.49 (dd, *J* = 17.0, 10.1 Hz, 1H), 6.31 (dd, *J* = 16.9, 1.6 Hz, 1H), 5.84–5.78 (m, 1H), 3.79 (s, 2H), 3.55 (d, *J* = 12.3 Hz, 2H), 3.16 (dd, *J* = 30.6, 8.5 Hz, 3H), 1.40 (s, 9H). ^13^C NMR (400 MHz, DMSO) δ 166.50, 163.82, 155.80, 154.31, 148.16, 143.61, 141.21, 133.48, 132.22, 130.95, 130.01, 128.43, 127.73, 125.21, 123.14, 119.81, 115.65, 79.73, 46.84, 41.56, 28.47.

N-{4-[4-(4-Isopropyl-piperazine-1-carbonyl)-quinolin-2-yl]-phenyl}-acrylamide (P3) Derived by crystallization in EtOAc as white powder (0.46 g, yield of 69%). HRMS C_26_H_28_N_4_O_2_ [M + H]^+^ calc.429.22458, found 429.22415. ^1^H NMR (400 MHz, DMSO) δ 10.40 (s, 1H), 8.32 (d, *J* = 8.7 Hz, 2H), 8.14–8.07 (m, 2H), 7.87 (d, *J* = 8.6 Hz, 2H), 7.79 (dd, *J* = 17.5, 8.0 Hz, 2H), 7.64 (t, *J* = 7.5 Hz, 1H), 6.49 (dd, *J* = 16.9, 10.1 Hz, 1H), 6.31 (dd, *J* = 16.9, 1.4 Hz, 1H), 5.84–5.78 (m, 1H), 5.76 (s, 1H), 3.88 (s, 1H), 3.68 (d, *J* = 8.9 Hz, 1H), 3.17 (d, *J* = 17.0 Hz, 1H), 3.10 (s, 1H), 2.61 (dd, *J* = 23.6, 6.3 Hz, 2H), 2.41 (s, 1H), 2.23 (d, *J* = 7.2 Hz, 1H), 0.96 (d, *J* = 6.5 Hz, 6H). ^13^C NMR (400 MHz, DMSO) δ 166.11, 163.82, 155.83, 148.12, 143.96, 141.19, 133.48, 132.23, 130.92, 130.03, 128.44, 127.79, 127.61, 125.09, 123.16, 119.82, 115.51, 54.20, 48.95, 48.32, 47.56, 42.05, 18.55.

N-(4-{4-[4-(3-Chloro-phenyl)-piperazine-1-carbonyl]-quinolin-2-yl}-phenyl)-acrylamide (P4) Derived by crystallization in EtOAc as white powder (0.23 g, yield of 73%). HRMS C_29_H_25_ClN_4_O_2_ [M + H]^+^ calc.497.16364, found 497.16895. ^1^H NMR (400 MHz, DMSO) δ 10.39 (s, 1H), 8.34 (d, *J* = 8.6 Hz, 2H), 8.17–8.10 (m, 2H), 7.90–7.80 (m, 4H), 7.63 (t, *J* = 7.6 Hz, 1H), 7.22 (t, *J* = 8.1 Hz, 1H), 6.97 (s, 1H), 6.91 (d, *J* = 8.4 Hz, 1H), 6.82 (d, *J* = 7.7 Hz, 1H), 6.49 (dd, *J* = 16.9, 10.1 Hz, 1H), 6.35–6.27 (m, 1H), 5.85–5.77 (m, 1H), 3.98 (s, 1H), 3.91 (s, 1H), 3.43 (t, *J* = 4.9 Hz, 2H), 3.28–3.19 (m, 2H), 3.04 (s, 1H), 2.69 (s, 1H). ^13^C NMR (400 MHz, DMSO) δ 166.30, 163.83, 155.84, 152.32, 148.16, 143.67, 141.22, 134.33, 133.47, 132.22, 130.98, 130.04, 128.45, 127.76, 125.20, 123.15, 119.82, 119.08, 115.63, 114.64, 48.54, 48.21, 46.63, 41.36, 38.72.

N-(4-{4-[4-(4-Chloro-phenyl)-piperazine-1-carbonyl]-quinolin-2-yl}-phenyl)-acrylamide (P5) Derived by crystallization in EtOAc as white powder (0.23 g, yield of 74%). HRMS C_29_H_25_C_l_N_4_O_2_ [M + H]^+^ calc. 497.16996, found 497.16888. ^1^H NMR (400 MHz, DMSO) δ 10.40 (s, 1H), 8.34 (d, *J* = 8.7 Hz, 2H), 8.18–8.10 (m, 2H), 7.88 (d, *J* = 8.7 Hz, 2H), 7.85–7.80 (m, 2H), 7.63 (t, *J* = 7.6 Hz, 1H), 7.25 (d, *J* = 8.9 Hz, 2H), 6.96 (d, *J* = 8.9 Hz, 2H), 6.49 (dd, *J* = 16.9, 10.1 Hz, 1H), 6.31 (dd, *J* = 16.9, 1.6 Hz, 1H), 5.84–5.77 (m, 1H), 3.99 (d, *J* = 7.1 Hz, 1H), 3.92 (s, 1H), 3.36 (d, *J* = 8.8 Hz, 3H), 3.27 (s, 1H), 3.15 (s, 1H), 2.99 (d, *J* = 5.8 Hz, 1H). ^13^C NMR (400 MHz, DMSO) δ 166.28, 163.84, 155.84, 149.91, 148.16, 143.68, 141.23, 133.46, 132.23, 130.97, 130.04, 129.18, 128.44, 127.74, 125.18, 123.40, 123.16, 119.83, 117.87, 115.68, 48.95, 48.58, 46.71, 41.39, 14.56.

N-(4-{4-[4-(2-Fluoro-phenyl)-piperazine-1-carbonyl]-quinolin-2-yl}-phenyl)-acrylamide (P6) Derived by crystallization in EtOAc as white powder (0.57 g, yield of 76%). HRMS C_29_H_25_FN_4_O_2_ [M + H]^+^ calc. 481.19951, found 481.19855. ^1^H NMR (400 MHz, DMSO) δ 10.39 (s, 1H), 8.34 (d, *J* = 8.7 Hz, 2H), 8.17–8.10 (m, 2H), 7.91–7.80 (m, 4H), 7.65 (t, *J* = 7.6 Hz, 1H), 7.18–7.07 (m, 3H), 7.00 (dd, *J* = 13.3, 6.5 Hz, 1H), 6.49 (dd, *J* = 16.9, 10.1 Hz, 1H), 6.31 (dd, *J* = 17.0, 1.5 Hz, 1H), 5.84–5.78 (m, 1H), 4.10–4.03 (m, 1H), 3.98–3.88 (m, 1H), 3.36 (d, *J* = 4.8 Hz, 1H), 3.28 (s, 1H), 3.23 (d, *J* = 4.4 Hz, 2H), 3.02 (s, 1H), 2.85 (d, *J* = 7.0 Hz, 1H). ^13^C NMR (400 MHz, DMSO) δ 166.35, 163.83, 156.63, 155.85, 154.20, 148.17, 143.69, 141.21, 139.86, 133.50, 132.22, 130.96, 130.04, 128.46, 127.75, 125.24, 123.42 (s), 123.19, 120.16, 119.83, 116.59, 116.39, 115.68, 50.90, 50.57, 47.18, 41.75.

N-(4-{4-[4-(4-Methoxy-phenyl)-piperazine-1-carbonyl]-quinolin-2-yl}-phenyl)-acrylamide (P7) Derived by crystallization in EtOAc as white powder (0.54 g, yield of 70%). HRMS C_30_H_28_N_4_O_3_ [M + H]^+^ calc. 493.21950, found 493.21820. ^1^H NMR (400 MHz, DMSO) δ 10.39 (s, 1H), 8.34 (d, *J* = 8.7 Hz, 2H), 8.17–8.10 (m, 2H), 7.88 (d, *J* = 8.7 Hz, 2H), 7.82 (d, *J* = 7.8 Hz, 2H), 7.64 (t, *J* = 7.6 Hz, 1H), 6.91 (d, *J* = 9.1 Hz, 2H), 6.82 (d, *J* = 9.0 Hz, 2H), 6.49 (dd, *J* = 16.9, 10.1 Hz, 1H), 6.31 (dd, *J* = 17.0, 1.5 Hz, 1H), 5.84–5.77 (m, 1H), 4.07–3.97 (m, 1H), 3.96–3.82 (m, 1H), 3.68 (s, 3H), 3.29 (s, 1H), 3.29–3.16 (m, 3H), 3.01 (s, 1H), 2.83 (d, *J* = 7.0 Hz, 1H). ^13^C NMR (400 MHz, DMSO) δ 166.23, 163.83, 155.84, 153.84, 148.16, 145.42, 143.78, 141.21, 133.48, 132.22, 130.97, 130.04, 128.45, 127.75, 125.17, 123.18, 119.82, 118.59, 115.64, 114.75, 55.64, 50.66, 50.35, 47.08, 41.68.

N-(4-{4-[4-(2-Methoxy-phenyl)-piperazine-1-carbonyl]-quinolin-2-yl}-phenyl)-acrylamide (P8) Derived by crystallization in EtOAc as white powder (0.60 g, yield of 78%). HRMS C_30_H_28_N_4_O_3_ [M + H]^+^ calc. 493.21950, found 493.21841. ^1^H NMR (400 MHz, DMSO) δ 10.50 (s, 1H), 8.34 (d, *J* = 8.8 Hz, 2H), 8.19–8.08 (m, 2H), 7.90 (d, *J* = 8.7 Hz, 2H), 7.83 (dd, *J* = 12.5, 6.6 Hz, 2H), 7.65 (t, *J* = 7.5 Hz, 1H), 7.01–6.83 (m, 6H), 6.53 (dd, *J* = 16.9, 10.1 Hz, 1H), 6.31 (dd, *J* = 17.0, 1.6 Hz, 1H), 5.80 (dd, *J* = 10.2, 1.6 Hz, 1H), 3.89 (d, *J* = 4.6 Hz, 1H), 3.31–3.22 (m, 1H), 3.16 (d, *J* = 4.2 Hz, 2H), 2.97 (d, *J* = 7.4 Hz, 2H), 2.75 (dd, *J* = 19.4, 7.5 Hz, 2H), 2.64–2.55 (m, 1H). ^13^C NMR (400 MHz, DMSO) δ 166.32, 163.86, 155.86, 152.45, 148.16, 143.81, 141.27, 141.07, 133.46, 132.30, 130.93, 130.04, 128.42, 127.66, 125.19, 123.45, 123.21, 121.25, 119.82, 118.90, 115.64, 112.38, 60.23, 55.81, 50.91, 50.55, 47.42, 41.93, 21.24, 14.56.

N-{4-[4-(4-Phenethyl-piperazine-1-carbonyl)-quinolin-2-yl]-phenyl}-acrylamide (P9) Derived by crystallization in EtOAc as white powder (0.62 g, yield of 81%). HRMS C_31_H_30_N_4_O_2_ [M + H]^+^ calc. 491.24023, found 491.23877. ^1^H NMR (400 MHz, DMSO) δ 10.39 (s, 1H), 8.33 (d, *J* = 8.6 Hz, 2H), 8.15–8.07 (m, 2H), 7.90–7.76 (m, 4H), 7.64 (t, *J* = 7.5 Hz, 1H), 7.22 (ddt, *J* = 21.2, 14.2, 7.2 Hz, 5H), 6.49 (dd, *J* = 16.9, 10.1 Hz, 1H), 6.32 (d, *J* = 16.8 Hz, 1H), 5.81 (d, *J* = 10.1 Hz, 1H), 3.92 (s, 1H), 3.71 (s, 1H), 3.15 (d, *J* = 20.1 Hz, 2H), 2.72 (dd, *J* = 15.6, 7.4 Hz, 3H), 2.55 (dd, *J* = 14.7, 7.8 Hz, 3H), 2.45 (s, 1H), 2.24 (s, 1H). ^13^C NMR (400 MHz, DMSO) δ 166.17, 163.82, 155.83, 148.13, 143.90, 141.20, 140.72, 133.48, 132.23, 130.93, 130.04, 129.11, 128.69, 128.44, 127.72, 126.33, 125.10, 123.16, 119.82, 115.52, 59.87, 53.29, 52.74, 47.15, 41.67, 38.72, 33.06.

2-(4-Acryloylamino-phenyl)-quinoline-4-carboxylic acid (4-bromo-phenyl)-amide (P10) Derived by crystallization in EtOAc as white powder (0.53 g, yield of 72%). HRMS C_25_H_18_BrN_3_O_2_ [M + H]^+^ calc. 472.06159, found 472.05997. ^1^H NMR (400 MHz, DMSO) δ 10.95 (s, 1H), 10.40 (s, 1H), 8.40–8.33 (m, 3H), 8.14 (dd, *J* = 8.2, 4.1 Hz, 2H), 7.92–7.77 (m, 5H), 7.64 (dd, *J* = 18.5, 8.1 Hz, 3H), 6.49 (dd, *J* = 16.9, 10.1 Hz, 1H), 6.31 (dd, *J* = 17.0, 1.4 Hz, 1H), 5.85–5.78 (m, 1H). ^13^C NMR (400 MHz, DMSO) δ 165.95, 163.85, 155.76, 148.42, 143.13, 141.26, 138.71, 133.46, 132.19, 130.80, 129.98, 128.47, 127.74, 125.50, 123.38, 122.39, 119.87, 116.96, 116.33.

2-(4-Acryloylamino-phenyl)-quinoline-4-carboxylic acid (4-chloro-phenyl)-amide (P11) Derived by crystallization in EtOAc as white powder (0.51 g, yield of 76%). HRMS C_25_H_18_ClN_3_O_2_ [M + H]^+^ calc. 428.11211, found 428.11087. ^1^H NMR (400 MHz, DMSO) δ 10.96 (s, 1H), 10.40 (s, 1H), 8.41–8.34 (m, 3H), 8.15 (d, *J* = 8.6 Hz, 2H), 7.93–7.81 (m, 5H), 7.65 (t, *J* = 7.7 Hz, 1H), 7.49 (d, *J* = 8.8 Hz, 2H), 6.50 (dd, *J* = 16.9, 10.1 Hz, 1H), 6.36–6.28 (m, 1H), 5.84–5.78 (m, 1H). ^13^C NMR (400 MHz, DMSO) δ 165.94, 163.85, 155.76, 148.42, 143.14, 141.26, 138.30, 133.46, 132.22, 130.80, 129.98, 129.26, 128.47, 128.24, 127.74, 125.51, 123.40, 122.03, 119.87, 116.97.

2-(4-Acryloylamino-phenyl)-quinoline-4-carboxylic acid p-tolylamide (P12) Derived by crystallization in EtOAc as white powder (0.53 g, yield of 82%). HRMS C_26_H_21_N_3_O_2_ [M + H]^+^ calc. 408.16673, found 408.16550 [M + H]. ^1^H NMR (400 MHz, DMSO) δ 10.74 (s, 1H), 10.40 (s, 1H), 8.34 (d, *J* = 23.1 Hz, 3H), 8.14 (s, 2H), 7.77 (dd, *J* = 76.6, 25.5 Hz, 6H), 7.22 (s, 2H), 6.48 (d, *J* = 9.4 Hz, 1H), 6.32 (d, *J* = 16.6 Hz, 1H), 5.81 (d, *J* = 8.3 Hz, 1H), 2.32 (s, 3H). ^13^C NMR (400 MHz, DMSO) δ 165.64, 163.85, 155.77, 148.43, 143.55, 141.22, 136.86, 133.58, 132.23, 130.73, 129.95, 129.68, 128.47, 127.82, 127.55, 125.59, 123.54, 120.47, 119.87, 116.85, 21.04.

2-(4-Acryloylamino-phenyl)-quinoline-4-carboxylic acid (4-ethyl-phenyl)-amide (P13) Derived by crystallization in EtOAc as white powder (0.53 g, yield of 80%). HRMS C_27_H_23_N_3_O_2_ [M + H]^+^ calc. 422.18238, found 422.18082. ^1^H NMR (400 MHz, DMSO) δ 10.75 (s, 1H), 10.41 (s, 1H), 8.34 (d, *J* = 23.9 Hz, 3H), 8.14 (s, 2H), 7.78 (dd, *J* = 72.6, 31.3 Hz, 6H), 7.25 (s, 2H), 6.48 (d, *J* = 8.8 Hz, 1H), 6.32 (d, *J* = 16.2 Hz, 1H), 5.81 (d, *J* = 7.2 Hz, 1H), 2.61 (s, 2H), 1.20 (s, 3H). ^13^C NMR (400 MHz, DMSO) δ 165.64, 163.85, 155.76, 148.42, 143.56, 141.22, 140.11, 137.05, 133.54, 132.23, 130.73, 129.95, 128.48, 127.81, 127.55, 125.59, 123.54, 120.55, 119.87, 116.85, 28.19, 16.26.

2-(4-Acryloylamino-phenyl)-quinoline-4-carboxylic acid (4-fluoro-phenyl)-amide (P14) Derived by crystallization in EtOAc as white powder (0.41 g, yield of 63%) HRMS C_25_H_18_FN_3_O_2_ [M + H]^+^ calc. 412.14166, found 412.14032. ^1^H NMR (400 MHz, DMSO) δ 10.89 (s, 1H), 10.41 (s, 1H), 8.38 (s, 3H), 8.16 (s, 2H), 7.87 (s, 5H), 7.65 (s, 1H), 7.27 (s, 2H), 6.50 (s, 1H), 6.33 (d, *J* = 13.8 Hz, 1H), 5.78 (d, *J* = 18.5 Hz, 1H). ^13^C NMR (400 MHz, DMSO) δ 165.74, 163.85, 155.76, 148.43, 143.29, 141.25, 135.74, 133.49, 132.22, 130.78, 129.97, 128.46, 127.82, 127.61, 125.56, 123.46, 122.32, 119.87, 116.94, 116.05, 115.83, 55.39.

2-(4-Acryloylamino-phenyl)-quinoline-4-carboxylic acid (3,5-dimethoxy-phenyl)-amide (P15) Derived by crystallization in EtOAc as white powder (0.56 g, yield of 72%). HRMS C_27_H_23_N_3_O_4_ [M + H]^+^ calc.497. 454.17221, found 454.17041. ^1^H NMR (400 MHz, DMSO) δ 10.77 (s, 1H), 10.41 (s, 1H), 8.38 (d, *J* = 8.6 Hz, 2H), 8.31 (s, 1H), 8.14 (dd, *J* = 8.3, 3.0 Hz, 2H), 7.90 (d, *J* = 8.6 Hz, 2H), 7.84 (t, *J* = 7.7 Hz, 1H), 7.65 (t, *J* = 7.7 Hz, 1H), 7.10 (d, *J* = 1.8 Hz, 2H), 6.50 (dd, *J* = 16.9, 10.1 Hz, 1H), 6.34 (s, 2H), 5.81 (d, *J* = 11.3 Hz, 1H), 3.77 (s, 6H). ^13^C NMR (400 MHz, DMSO) δ 165.90, 163.85, 161.02, 155.75, 148.41, 143.35, 141.24, 140.98, 133.50, 132.22, 130.77, 129.96, 128.47, 127.82, 127.61, 125.53, 123.42, 119.87, 116.86, 98.78, 96.61, 60.23, 55.67.

2-(4-Acryloylamino-phenyl)-quinoline-4-carboxylic acid (4-methoxy-phenyl)-amide (P16) Derived by crystallization in EtOAc as white powder (0.48 g, yield of 73%). HRMS C_26_H_21_N_3_O_3_ [M + H]+ calc. 424.16165, found 424.15900. ^1^H NMR (400 MHz, DMSO) δ 10.68 (s, 1H), 10.40 (s, 1H), 8.37 (d, *J* = 8.6 Hz, 2H), 8.30 (s, 1H), 8.14 (t, *J* = 7.9 Hz, 2H), 7.89 (d, *J* = 8.6 Hz, 2H), 7.83 (t, *J* = 7.7 Hz, 1H), 7.74 (d, *J* = 8.9 Hz, 2H), 7.64 (t, *J* = 7.6 Hz, 1H), 6.99 (d, *J* = 8.9 Hz, 2H), 6.49 (dd, *J* = 16.9, 10.1 Hz, 1H), 6.31 (d, *J* = 17.3 Hz, 1H), 5.81 (d, *J* = 11.4 Hz, 1H), 3.77 (s, 3H). ^13^C NMR (400 MHz, DMSO) δ 165.38, 163.84, 156.31, 155.76, 148.43, 143.58, 141.21, 133.54, 132.47, 132.23, 130.71, 129.94, 128.46, 127.81, 127.52, 125.63, 123.58, 122.02, 119.86, 116.85, 114.42, 55.72.

2-(4-Acryloylamino-phenyl)-quinoline-4-carboxylic acid (4-ethoxy-phenyl)-amide (P17) Derived by crystallization in EtOAc as white powder (0.50 g, yield of 73%). HRMS C_29_H_25_ClN_4_O_2_ [M + H]^+^ calc. 438.17730, found 438.17468. ^1^H NMR (400 MHz, DMSO) δ 10.67 (s, 1H), 10.40 (s, 1H), 8.37 (d, *J* = 8.6 Hz, 2H), 8.30 (s, 1H), 8.15 (t, *J* = 7.7 Hz, 2H), 7.89 (d, *J* = 8.6 Hz, 2H), 7.83 (t, *J* = 7.7 Hz, 1H), 7.73 (d, *J* = 8.9 Hz, 2H), 7.64 (t, *J* = 7.6 Hz, 1H), 6.97 (d, *J* = 8.9 Hz, 2H), 6.49 (dd, *J* = 16.9, 10.1 Hz, 1H), 6.31 (d, *J* = 16.2 Hz, 1H), 5.81 (d, *J* = 11.1 Hz, 1H), 4.04 (q, *J* = 6.9 Hz, 2H), 1.34 (t, *J* = 6.9 Hz, 3H). ^13^C NMR (400 MHz, DMSO) δ 165.37, 163.84, 155.76, 155.57, 148.43, 143.60, 141.21, 133.54, 132.37, 132.23, 130.71, 129.94, 128.46, 127.81, 127.51, 125.64, 123.58, 122.00, 119.86, 116.85, 114.93, 63.63, 15.17.

2- (4-Acryloylamino-phenyl)-quinoline-4-carboxylic acid (4-pentyl-phenyl)-amide (P18) Derived by crystallization in EtOAc as white powder (0.58 g, yield of 80%). HRMS C_30_H_29_N_3_O_2_ [M + H]^+^ calc. 464.22933, found 464.22659. ^1^H NMR (400 MHz, DMSO) δ 10.74 (s, 1H), 10.40 (s, 1H), 8.37 (d, *J* = 8.6 Hz, 2H), 8.31 (s, 1H), 8.14 (d, *J* = 8.6 Hz, 2H), 7.89 (d, *J* = 8.6 Hz, 2H), 7.84 (t, *J* = 7.7 Hz, 1H), 7.72 (d, *J* = 8.3 Hz, 2H), 7.64 (t, *J* = 7.6 Hz, 1H), 7.22 (d, *J* = 8.3 Hz, 2H), 6.49 (dd, *J* = 16.9, 10.1 Hz, 1H), 6.31 (d, *J* = 16.1 Hz, 1H), 5.81 (d, *J* = 11.3 Hz, 1H), 2.58 (t, *J* = 7.5 Hz, 2H), 1.65–1.53 (m, 2H), 1.37–1.23 (m, 4H), 0.88 (t, *J* = 6.9 Hz, 3H). ^13^C NMR (400 MHz, DMSO) δ 165.64, 163.84, 155.76, 148.43, 143.55, 141.23, 138.65, 137.04, 133.53, 132.23, 130.72, 129.95, 129.03, 128.45, 127.80, 127.53, 125.59, 123.55, 120.48, 119.86, 116.86, 35.07, 31.25, 22.46, 14.43.

3- (4-Acryloylamino-phenyl)-quinoline-4-carboxylic acid [4-(4-methyl-cyclohexyl)-phenyl]-amide (P19) Derived by crystallization in EtOAc as white powder (0.53 g, yield of 69%). HRMS C_32_H_31_N_3_O_2_ [M + H]^+^ calc. 491.24834, found 492.23212. ^1^H NMR (400 MHz, DMSO) δ 10.63 (s, 1H), 10.40 (s, 1H), 8.37 (d, *J* = 8.6 Hz, 2H), 8.29 (s, 1H), 8.14 (dd, *J* = 8.2, 5.1 Hz, 2H), 7.89 (d, *J* = 8.6 Hz, 2H), 7.84 (t, *J* = 7.7 Hz, 1H), 7.70 (d, *J* = 8.9 Hz, 2H), 7.64 (t, *J* = 7.6 Hz, 1H), 7.03 (d, *J* = 8.9 Hz, 2H), 6.50 (dd, *J* = 16.9, 10.1 Hz, 1H), 6.31 (d, *J* = 16.5 Hz, 1H), 5.81 (d, *J* = 11.4 Hz, 1H), 3.44 (s, 8H), 2.57 (s, 3H). ^13^C NMR (400 MHz, DMSO) δ 165.31, 163.86, 155.76, 148.44, 147.41, 143.61, 141.21, 133.56, 132.22, 131.81, 130.71, 129.95, 128.46, 127.83, 127.51, 126.92, 125.62, 124.57, 123.60, 121.62, 119.87, 119.41, 116.83, 116.51, 110.38, 53.82, 47.60, 44.23.

2-(4-Acryloylamino-phenyl)-quinoline-4-carboxylic acid benzo [1,3]dioxol-5-ylamide (P20) Derived by crystallization in EtOAc as white powder (0.51 g, yield of 74%). HRMS C_26_H_19_N_3_O_4_ [M + H]^+^ calc. 438.14091, found 438.13861. ^1^H NMR (400 MHz, DMSO) δ 10.72 (s, 1H), 10.40 (s, 1H), 8.37 (d, *J* = 8.6 Hz, 2H), 8.30 (s, 1H), 8.14 (d, *J* = 8.6 Hz, 2H), 7.89 (d, *J* = 8.6 Hz, 2H), 7.84 (t, *J* = 7.7 Hz, 1H), 7.64 (t, *J* = 7.6 Hz, 1H), 7.51 (s, 1H), 7.23 (d, *J* = 8.4 Hz, 1H), 6.96 (d, *J* = 8.4 Hz, 1H), 6.49 (dd, *J* = 17.0, 10.1 Hz, 1H), 6.31 (d, *J* = 16.6 Hz, 1H), 6.05 (s, 2H), 5.79 (t, *J* = 13.3 Hz, 1H). ^13^C NMR (400 MHz, DMSO) δ 165.49, 163.85, 155.75, 148.42, 147.61, 144.06, 143.45, 141.23, 133.65, 133.51, 132.22, 130.74, 129.95, 128.46, 127.82, 127.56, 125.60, 123.51, 119.87, 116.86, 113.53, 108.60, 102.56, 101.62, 60.23, 21.24, 14.56.

2-(4-Acryloylamino-phenyl)-quinoline-4-carboxylic acid (3-methoxy-phenyl)-amide (P21) Derived by crystallization in EtOAc as white powder (0.50 g, yield of 76%). HRMS C_26_H_21_N_3_O_3_ [M + H]^+^ calc. 424.16165, found 424.16431. ^1^H NMR (400 MHz, DMSO) δ 10.80 (s, 1H), 10.40 (s, 1H), 8.38 (d, *J* = 8.6 Hz, 2H), 8.32 (s, 1H), 8.14 (d, *J* = 8.5 Hz, 2H), 7.89 (d, *J* = 8.6 Hz, 2H), 7.84 (t, *J* = 7.7 Hz, 1H), 7.65 (t, *J* = 7.6 Hz, 1H), 7.52 (s, 1H), 7.38 (d, *J* = 8.1 Hz, 1H), 7.31 (t, *J* = 8.1 Hz, 1H), 6.76 (d, *J* = 8.1 Hz, 1H), 6.49 (dd, *J* = 16.9, 10.1 Hz, 1H), 6.31 (d, *J* = 17.1 Hz, 1H), 5.81 (d, *J* = 11.2 Hz, 1H), 3.78 (s, 3H). ^13^C NMR (400 MHz, DMSO) δ 165.88, 163.85, 160.04, 155.77, 148.42, 143.41, 141.24, 140.50, 133.51, 132.22, 130.76, 130.14, 129.96, 128.47, 127.82, 127.60, 125.54, 123.46, 119.87, 116.88, 112.72, 110.10, 106.25, 55.57.

### Enzyme Inhibitory Assay

All of the enzymatic reactions were conducted at 37°C for 30 min. The reaction mixture contains 25 mM Tris, 1 mM MgCl_2_, 0.1 mg/ml BSA, 137 mM NaCl, 2.7 mM KCl, SIRTs and the enzyme substrate. The compounds were diluted in 10% DMSO and 5 ul of the dilution was added to each reaction mixture (50 ul). The assay was performed by quantitating the amount of fluorescent product generated from the enzyme reaction. Fluorescence intensity is then analyzed at excitation wavelength of 350–360 nm and emission wavelength of 450–460 nm on a SpectraMax M5 microtiter plate reader. The IC_50_ values were calculated using Prism GraphPad software.

### Molecular Docking

The molecular docking process was performed using Glide (schrodinger Inc., supported by Shanghai Institute of Materia Medica Chinese Academy of Sciences). Crystal structure of SIRT1-3 (PDB Entry: 5BTR, 5Y0Z, 4JT8) was derived from RCSB protein data bank (www.rcsb.org). Structural modifications were performed to make the protein suitable for docking. The water molecules and the metals crystallized in the protein structure were removed. OPLS 2005 force field was assigned to the refined structure. The structure of molecule P6 was sketched by maestro and prepared by LigPrep. The docked ligand was confined to an enclosing box with size similar to the workspace ligand. Extra precision was applied in the docking process, and other parameters were set as default.

### *In Vitro* Antiproliferative Assay

The proliferation of THP-1, MOLM-13, SEM and MV4-11 cells was tested by CCK-8 assay. Briefly, cells were seeded in 96-well plate with about 5 × 10^3^ cells in each well. The cells were treated with P6 or Ara-C (0–10 µM) after 24 h of incubation. CCK-8 reagent (10 ml) was added to each well after 72 h of incubation, and cells were incubated at 37°C for 4 h. The light absorbance at 450 nm was measured by using an Opsys microplate reader (Dynex Technologirs, Chantilly, VA, United States). Results are illustrated as percent of cell viability normalized to DMSO-treated control cells.

### Colony Formation Assay

THP-1, MOLM-13, SEM and MV4-11 cells were cultured with P6 (0–2.0 µM) in 2.6% methycellulose medium containing 10% FBS in a 24-well flat-bottomed plate for 12 days (Qu et al., 2021). The number of individual colonies consisting of more than approximately 50 cells was counted by a CX43 microscope (Olympus, Shinjuku-ku, Tokyo, Japan) with an Olympus EP50 camera (Olympus, Shinjuku-ku, Tokyo, Japan).

### Cell Cycle Analysis

THP-1, MOLM-13, SEM and MV4-11 cells were incubated with 1.0 (THP-1, MOLM-13 cells) and 2.0 (SEM and MV4-11 cells) µM of P6 for 72 h. After treatment, cells were collected and fixed with 70% pre-cold ethanol in PBS and stored at −20°C for at least 24 h. Then the cells were stained with 50 mg/ml PI and 100 mg/ml RNase A for 30 min in the dark at room temperature. Finally, flow cytometry was used to detect the percentage of cells in the sub-G1, G0/G1, S, and G2/M phases with a Beckman Coulter DxFLEX flow cytometer (Florida, Miami, USA). The data was analyzed and fitted by ModFit software.

### Cell Apoptosis Analysis

THP-1, MOLM-13, SEM and MV4-11 cells were treated with P6 (0.45–4 µM) or Ara-C (0.1–2.0 µM) for 72 h, collected and resuspended in 1× binding buffer. Cells were incubated with FITC Annexin V and PI double labeling for 30 min in the dark at room temperature and measured by flow cytometry.

### Cell Morphology Analysis

THP-1, MOLM-13, SEM and MV4-11 cells were incubated with P6 or Ara-C for 72 h and then collected. Slides were made by cytospin and subsequently air dried. The cells were stained with Wright-Giemsa and observed for morphological features using a light microscope.

### Statistical Analysis

All experiments were repeated at least three times unless otherwise stated. The data were represented as mean ± SD. Statistical analysis were performed with Student’s t test for two group comparisons and using one-way ANOVA with Tukey’s post hoc test for multigroup comparisons. *p* < 0.05 or *p* < 0.01 were considered statistically significant.

## Data Availability

The original contributions presented in the study are included in the article/Supplementary Material, further inquiries can be directed to the corresponding authors.
