# Supplementary material for: Discovery of 2-(4-Acrylamidophenyl)-Quinoline-4-Carboxylic Acid Derivatives as Potent SIRT3 Inhibitors
Source: Front Chem. 2022 Mar 30;10:880067. doi: 10.3389/fchem.2022.880067 (PMC9005971; doi:10.3389/fchem.2022.880067)
Supplement: Supplementary file 1 [file DataSheet1.PDF]

Discovery of 2-(4-acrylamidophenyl)-quinoline-4-carboxylic acid Derivatives as Potent SIRT3 inhibitors

Qian Hui [a†], Xueming Li [b†], Wenli Fan[a†], Congying Gao[b], Lin Zhang[a], Hongyu Qin[a], Liuya Wei[b]* and Lei Zhang[a]*

[a] *Department of Medicinal Chemistry, School of Pharmacy, Weifang Medical University, Weifang, Shandong, China;*

[b] *Department of Inorganic Chemistry, School of Pharmacy, Weifang Medical University, Weifang, Shandong, China.*

[†]*These authors have contributed equally to this work.*

Author for correspondence: Liuya Wei, xiaoyawfmc@163.com; Lei Zhang, Tel./fax: +86-536-8462014, E-mail: leizhangchemical@gmail.com.

Contents

HRMS and ^{1}H-NMR spectrum of PB-1 1

HRMS and ^{1}H-NMR spectrum of PB-2 2

HRMS, ^{1}H-NMR and ^{13}C NMR spectrum of P1 3

HRMS, ^{1}H-NMR and ^{13}C NMR spectrum of P2 4

HRMS, ^{1}H-NMR and ^{13}C NMR spectrum of P3 6

HRMS, ^{1}H-NMR and ^{13}C NMR spectrum of P4 7

HRMS, ^{1}H-NMR and ^{13}C NMR spectrum of P5 9

HRMS, ^{1}H-NMR and ^{13}C NMR spectrum of P6 10

HRMS, ^{1}H-NMR and ^{13}C NMR spectrum of P7 12

HRMS, ^{1}H-NMR and ^{13}C NMR spectrum of P8 13

HRMS, ^{1}H-NMR and ^{13}C NMR spectrum of P9 15

HRMS, ^{1}H-NMR and ^{13}C NMR spectrum of P10 16

HRMS, ^{1}H-NMR and ^{13}C NMR spectrum of P11 18

HRMS, ^{1}H-NMR and ^{13}C NMR spectrum of P12 19

HRMS, ^{1}H-NMR and ^{13}C NMR spectrum of P13 21

HRMS, ^{1}H-NMR and ^{13}C NMR spectrum of P14 22

HRMS, ^{1}H-NMR and ^{13}C NMR spectrum of P15 24

HRMS, ^{1}H-NMR and ^{13}C NMR spectrum of P16 25

HRMS, ^{1}H-NMR and ^{13}C NMR spectrum of P17 27

HRMS, ^{1}H-NMR and ^{13}C NMR spectrum of P18 28

HRMS, ^{1}H-NMR and ^{13}C NMR spectrum of P19 30

HRMS, ^{1}H-NMR and ^{13}C NMR spectrum of P20 31

HRMS, ^{1}H-NMR and ^{13}C NMR spectrum of P21 33

SIRT1 enzyme inhibitory assay data 34

SIRT2 enzyme inhibitory assay data 37

SIRT3 enzyme inhibitory assay data 40

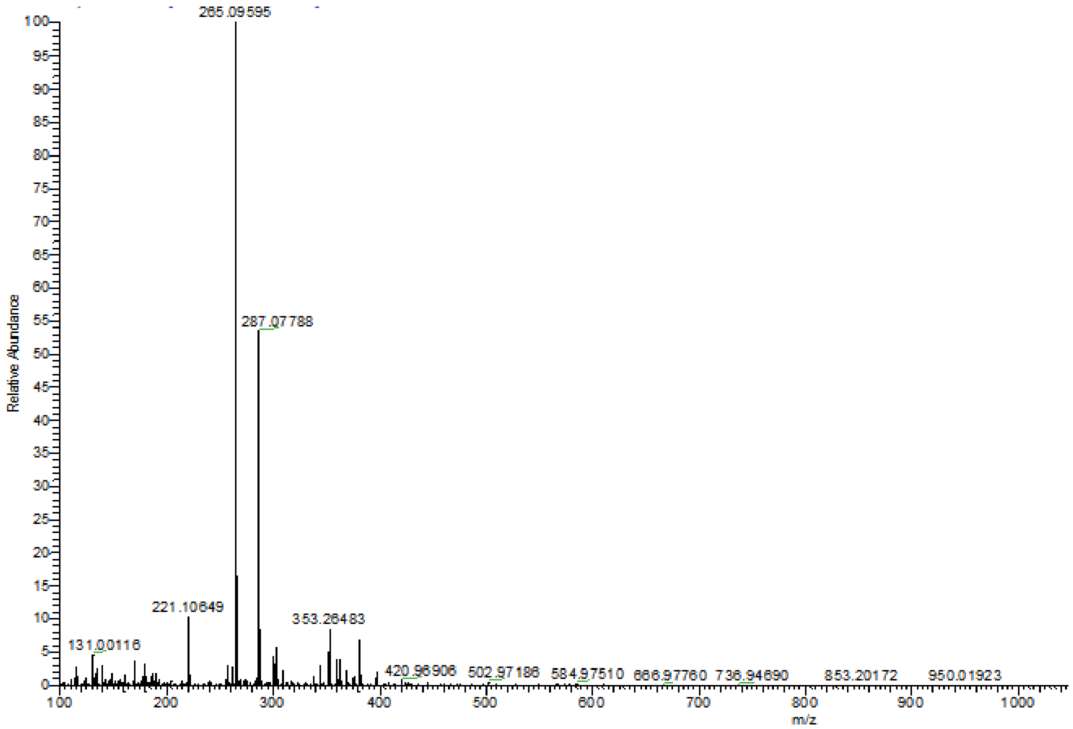

HRMS spectrum of PB-1

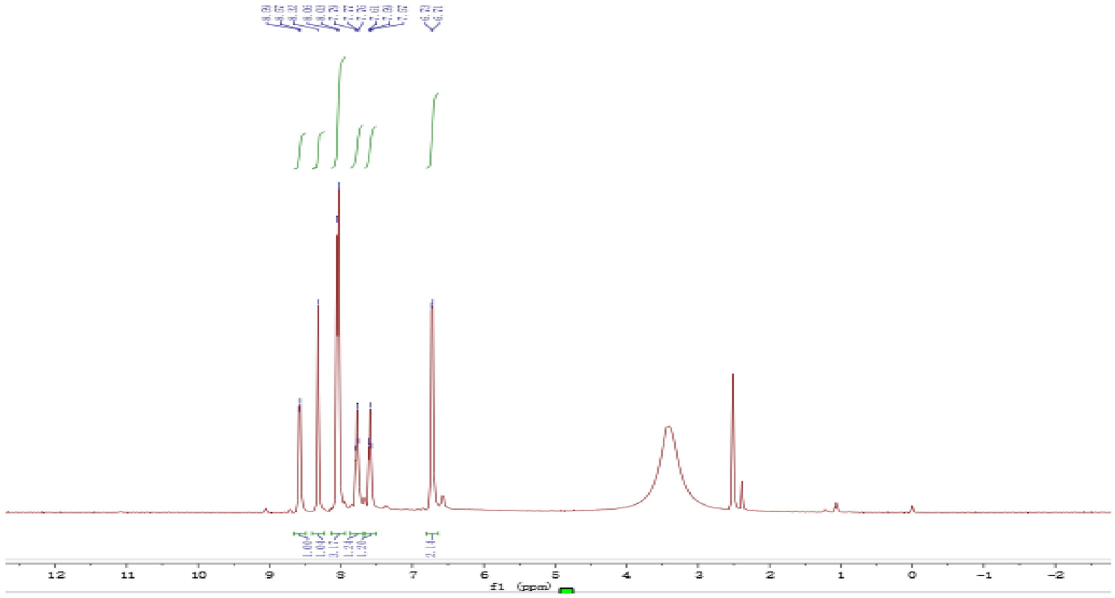

^{1}H NMR spectrum of PB-1

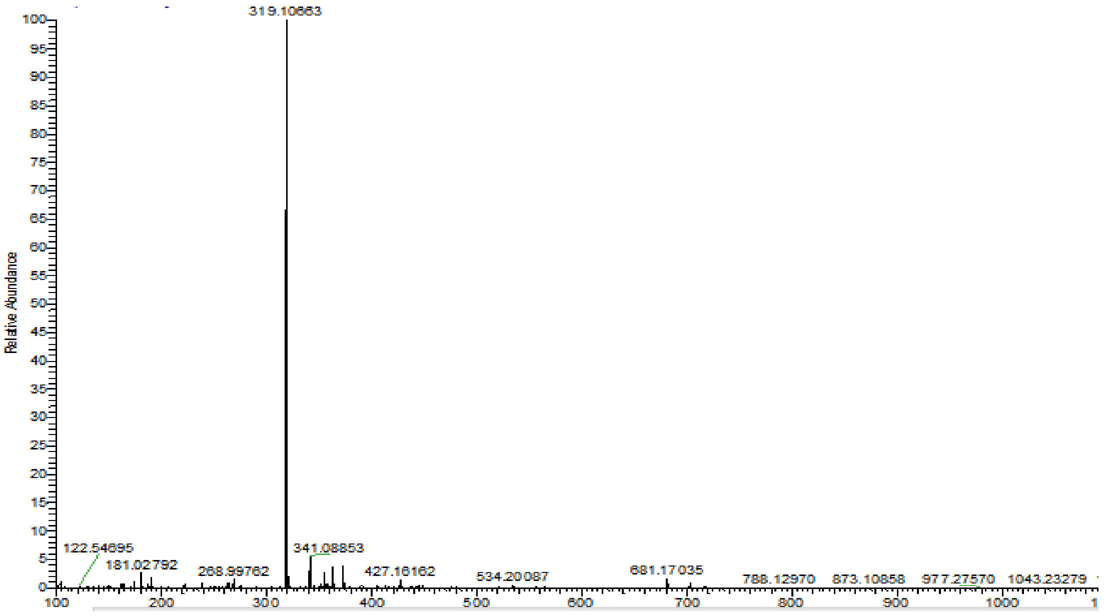

HRMS spectrum of PB-2

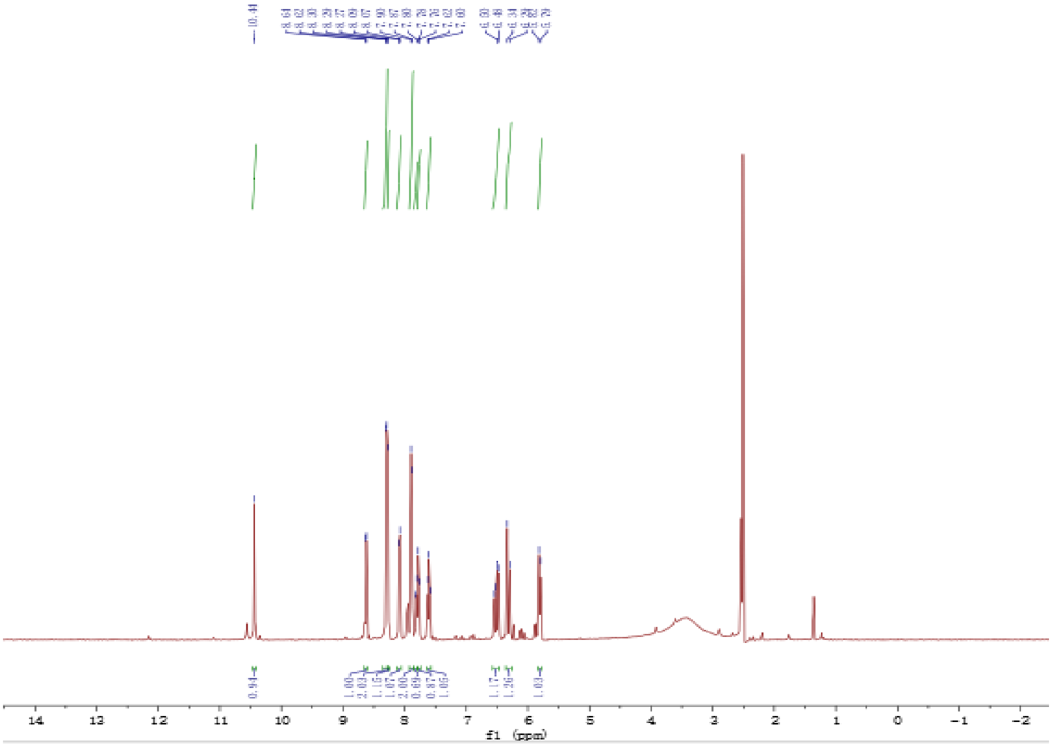

^{1}H NMR spectrum of PB-2

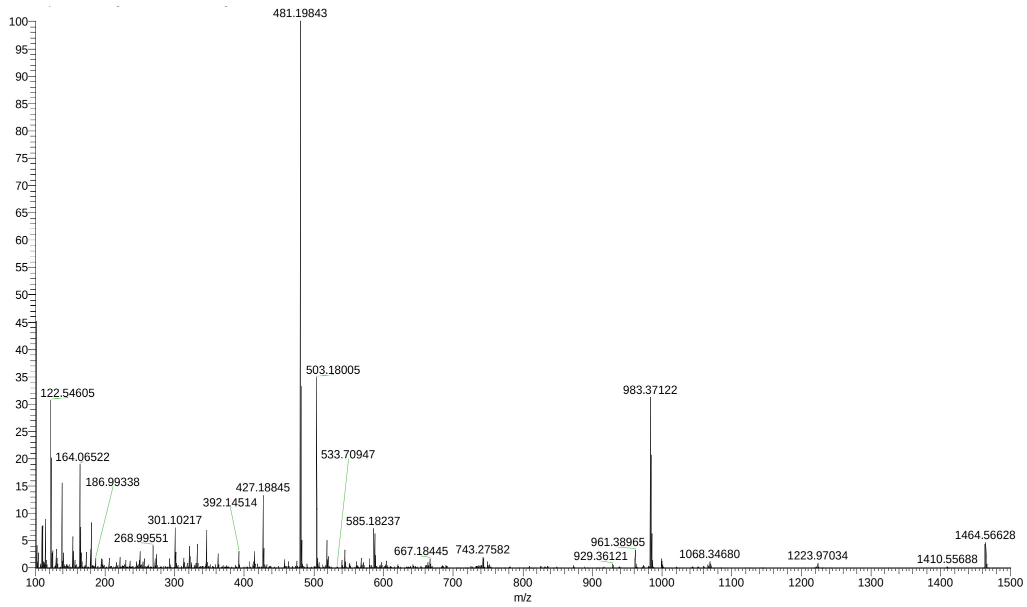

HRMS spectrum of P1

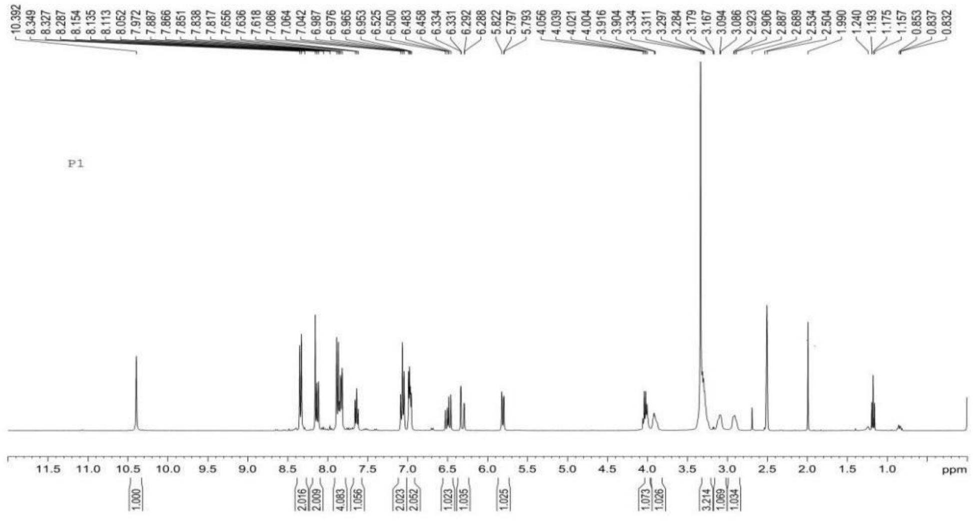

^{1}H NMR spectrum of P1

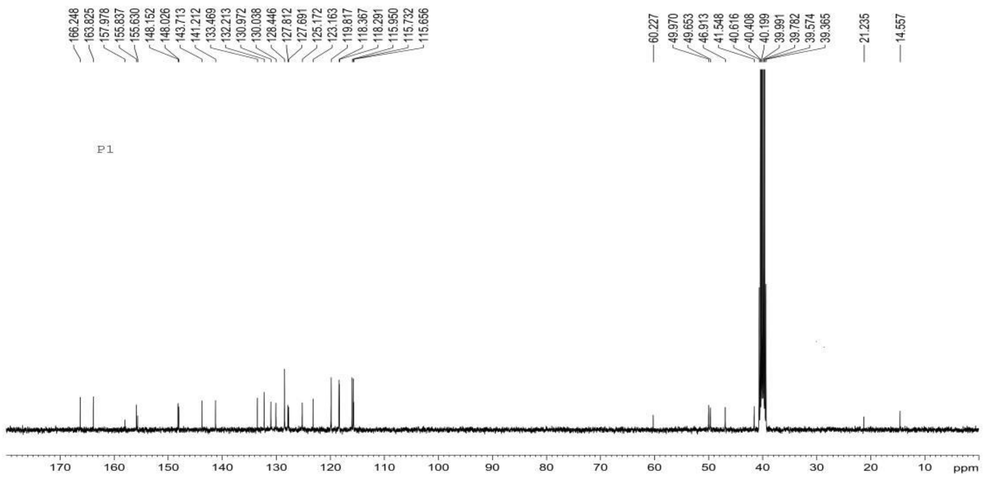

^{13}C NMR spectrum of P1

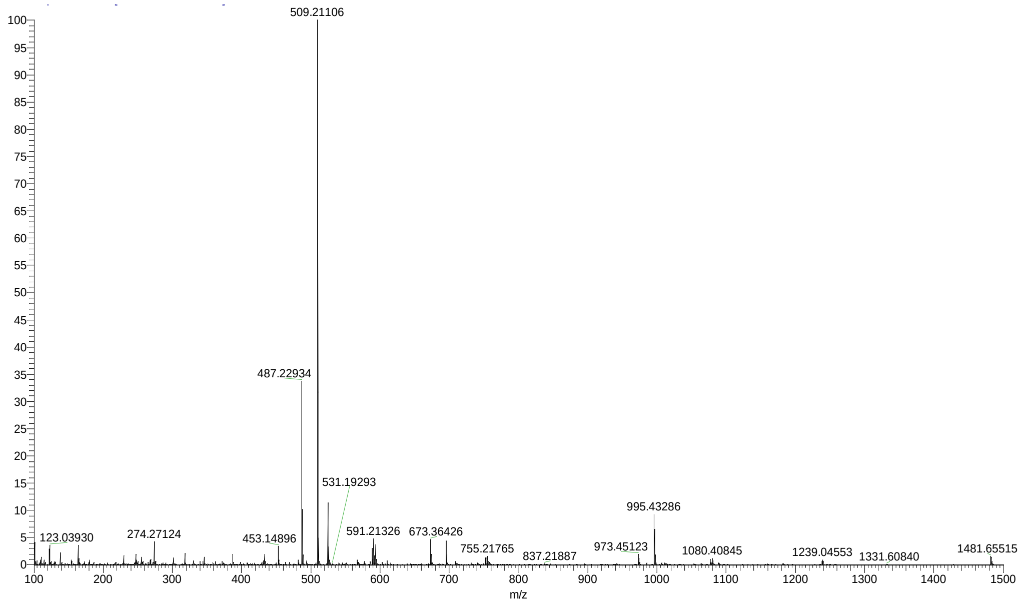

HRMS spectrum of P2

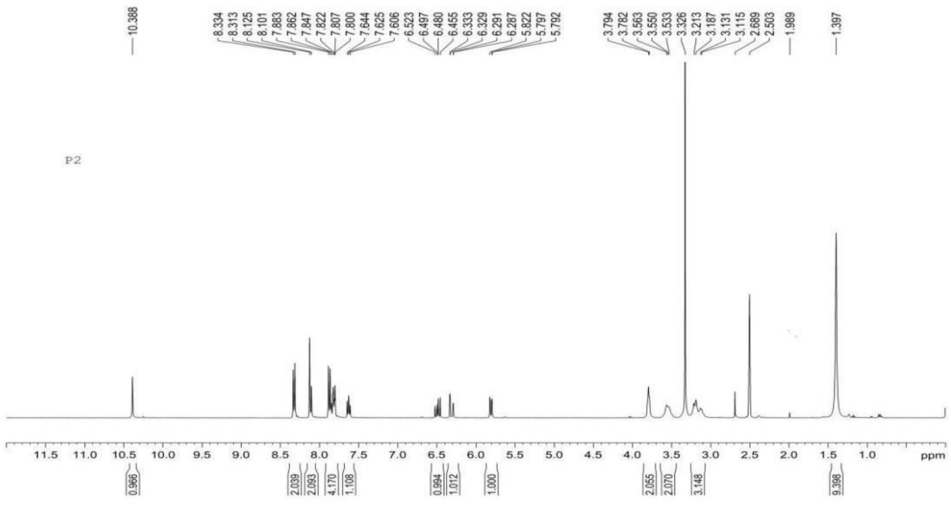

^{1}H NMR spectrum of P2

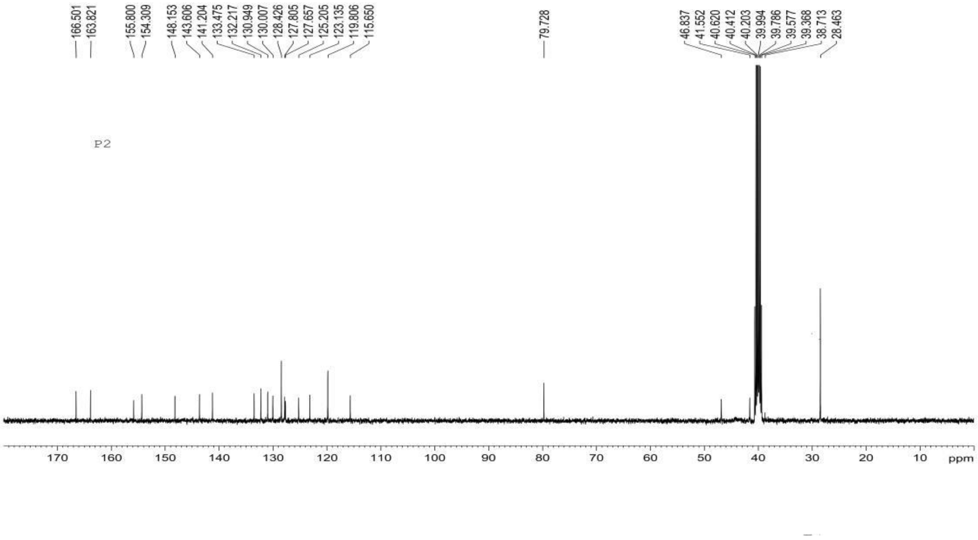

^{13}C NMR spectrum of P2

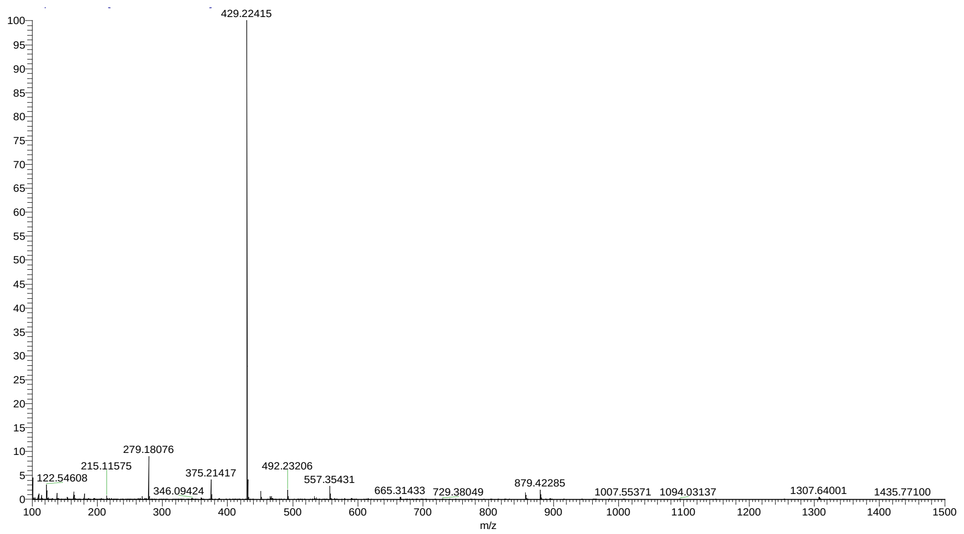

HRMS spectrum of P3

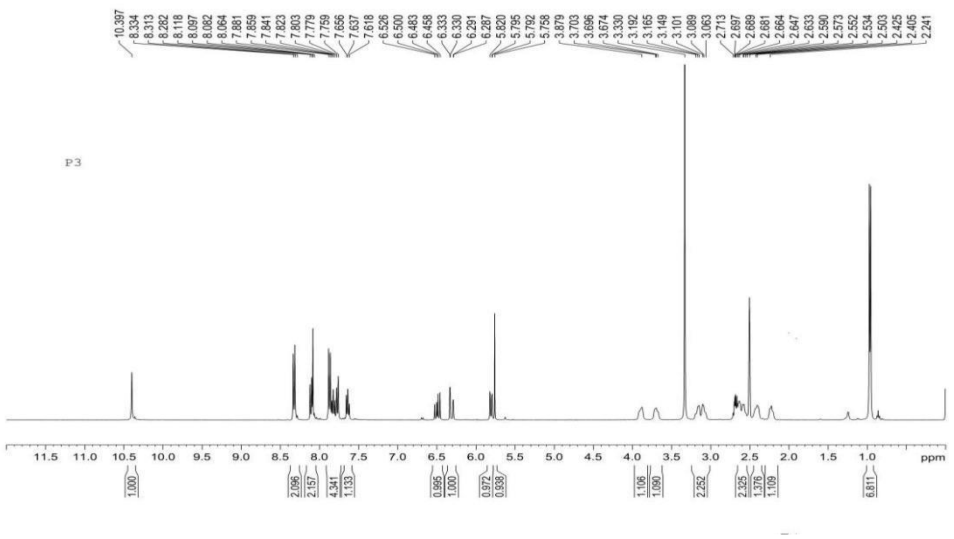

^{1}H NMR spectrum of P3

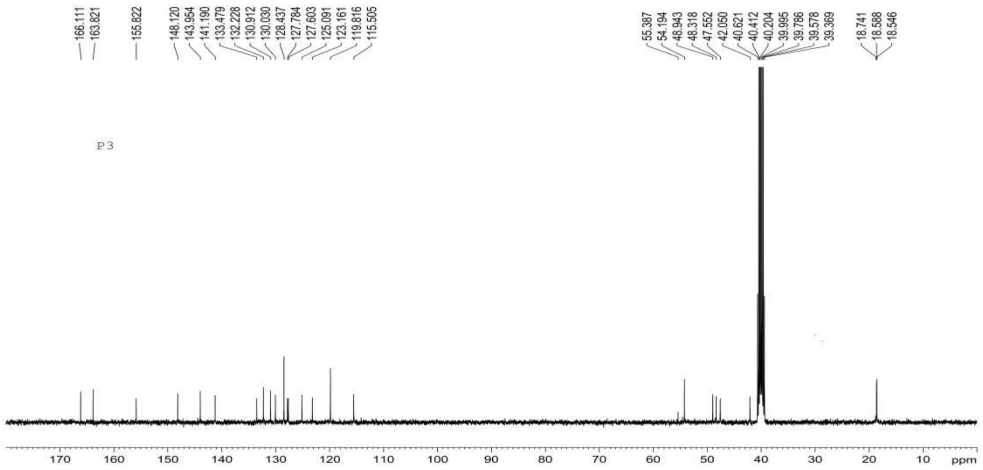

^{13}C NMR spectrum of P3

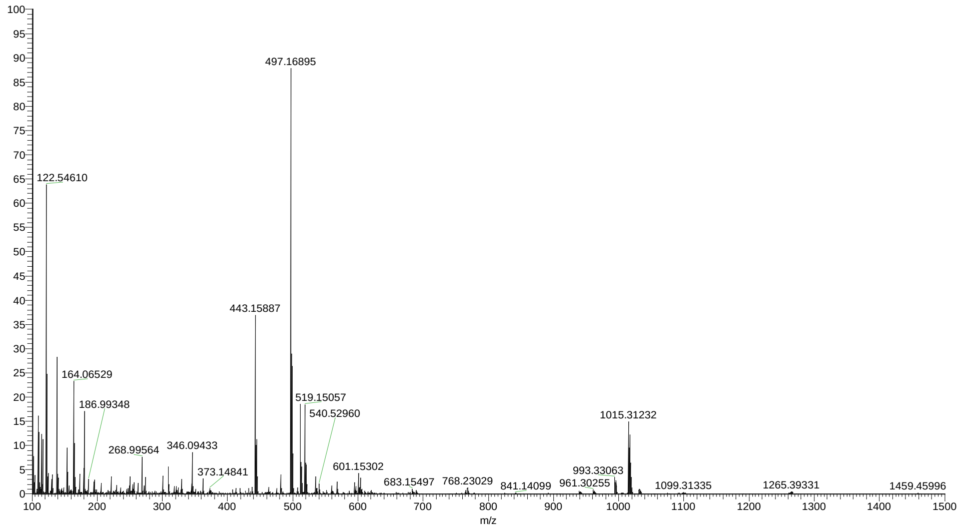

HRMS spectrum of P4

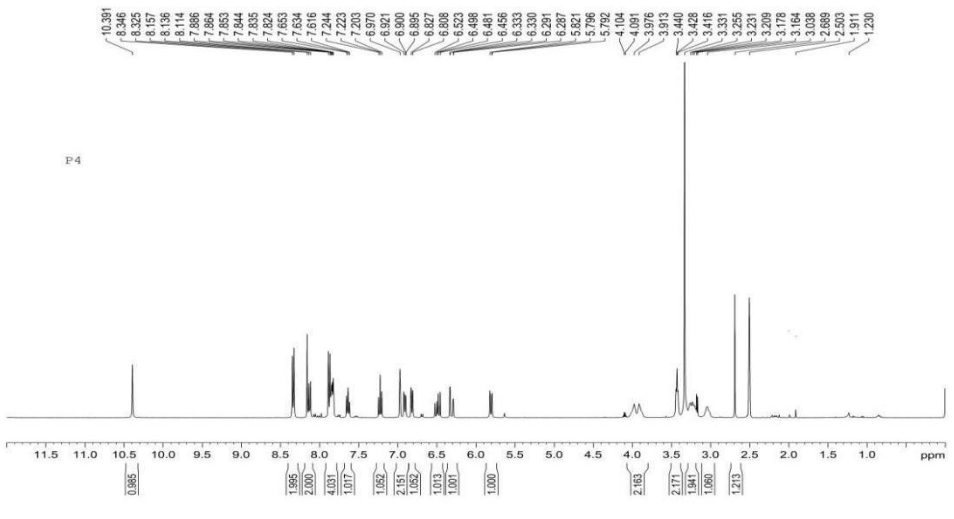

^{1}H NMR spectrum of P4

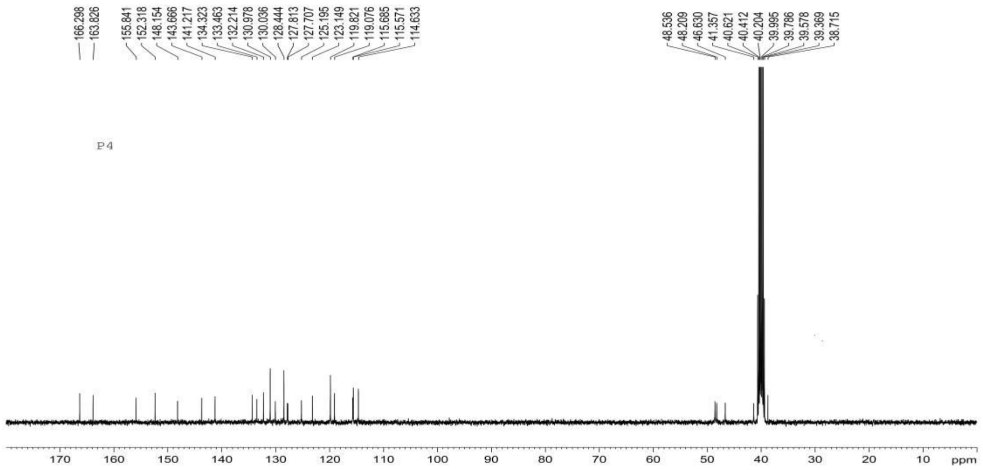

^{13}C NMR spectrum of P4

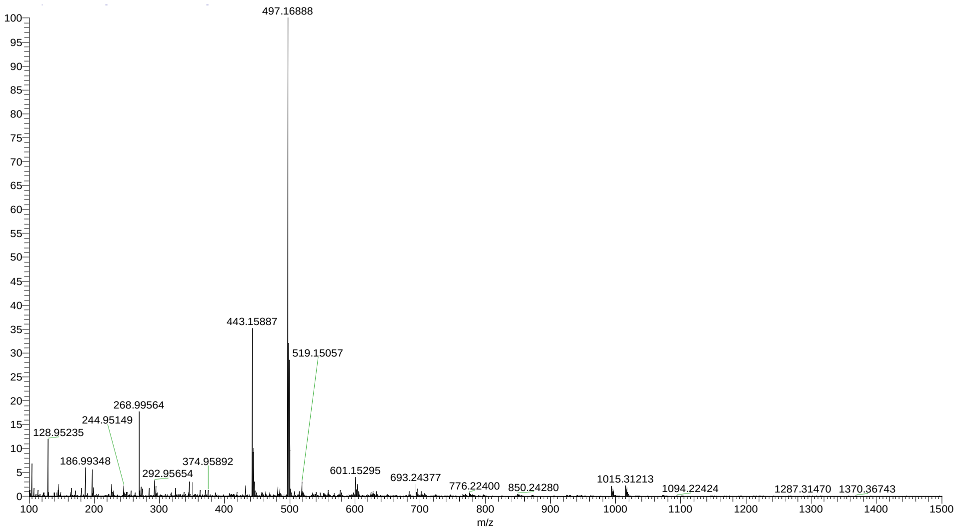

HRMS spectrum of P5

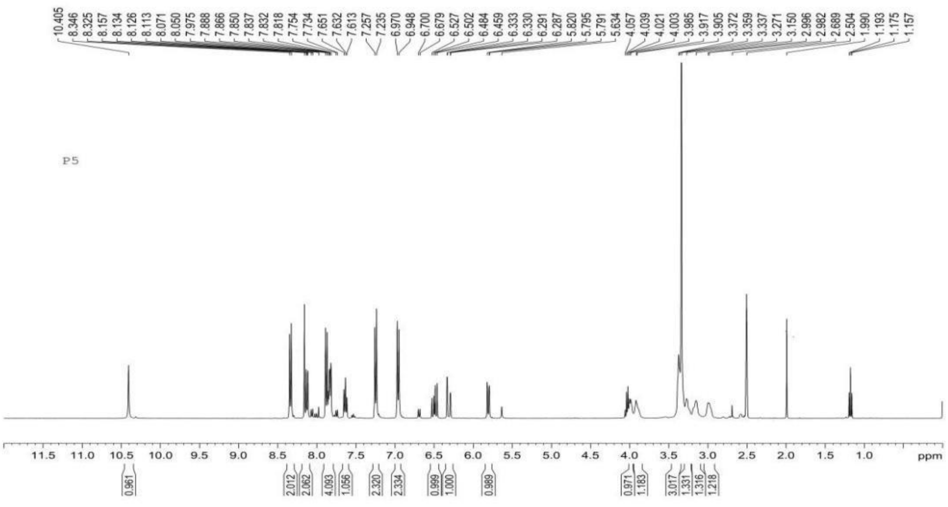

^{1}H NMR spectrum of P5

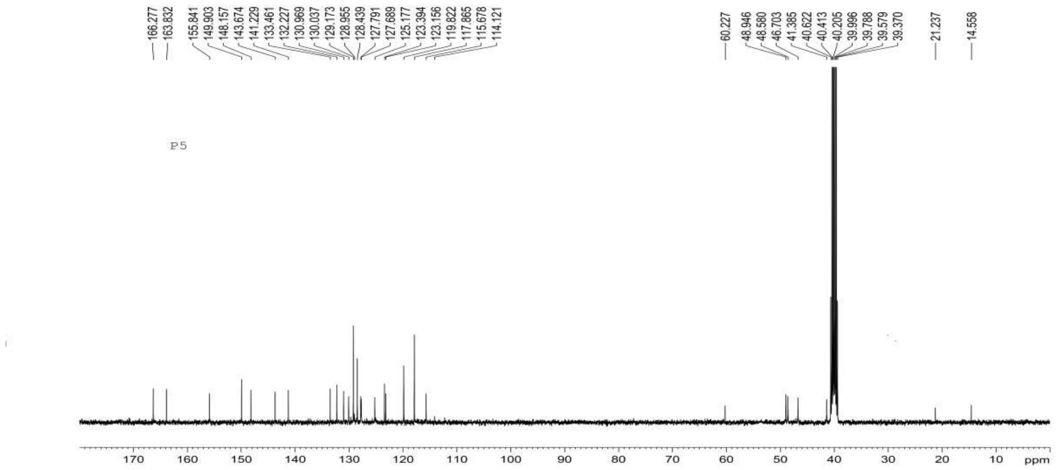

^{13}C NMR spectrum of P5

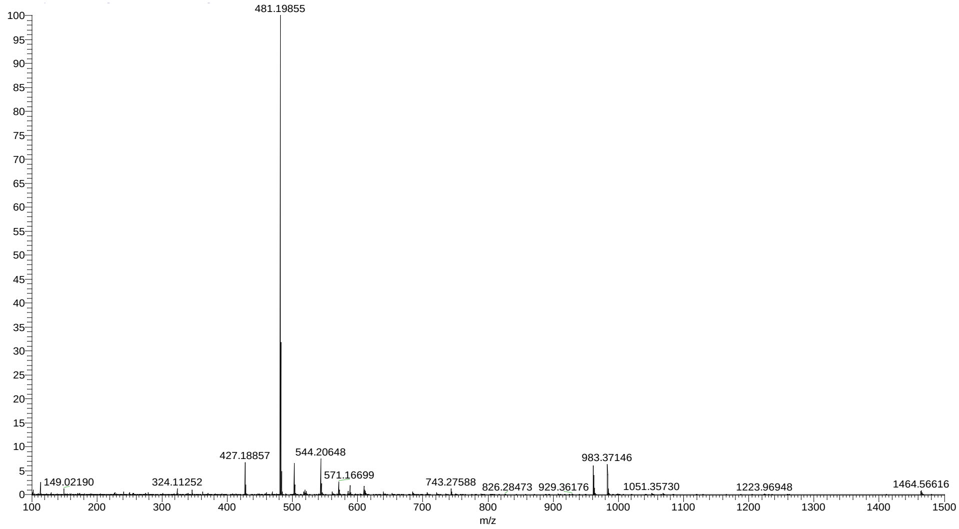

HRMS spectrum of P6

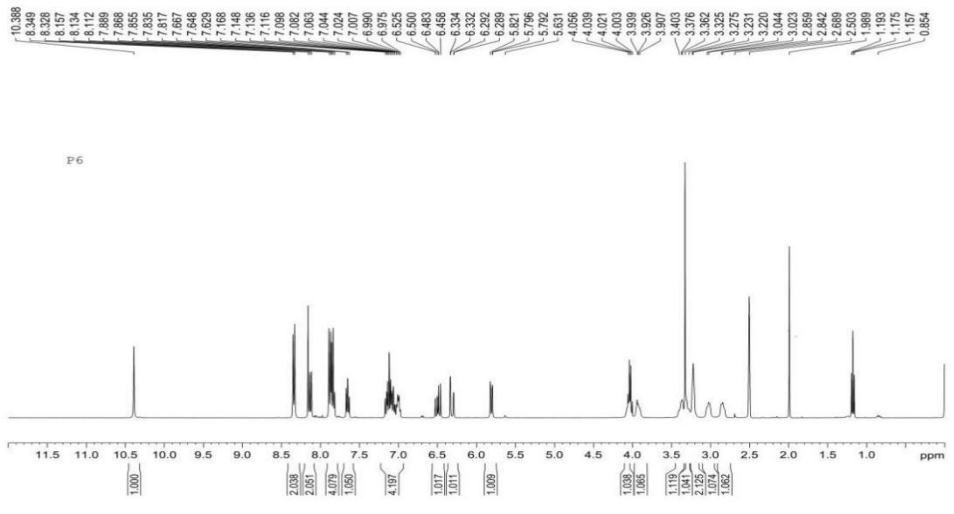

^{1}H NMR spectrum of P6

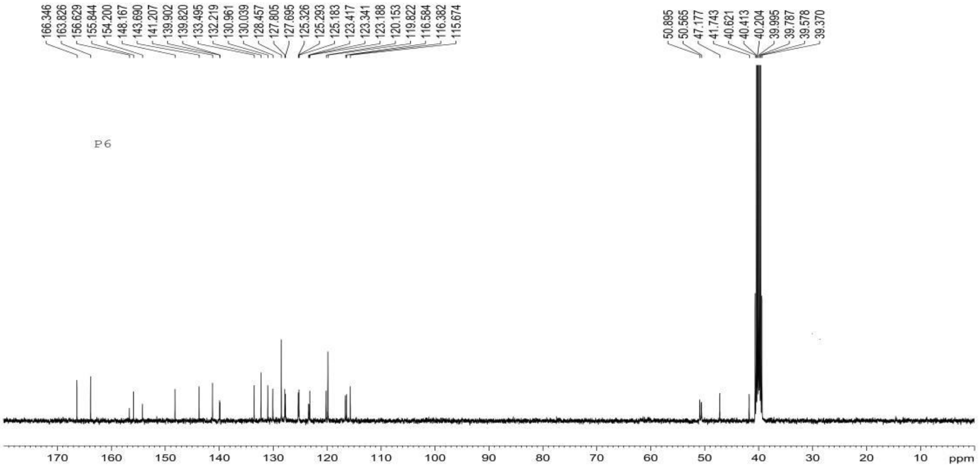

^{13}C NMR spectrum of P6

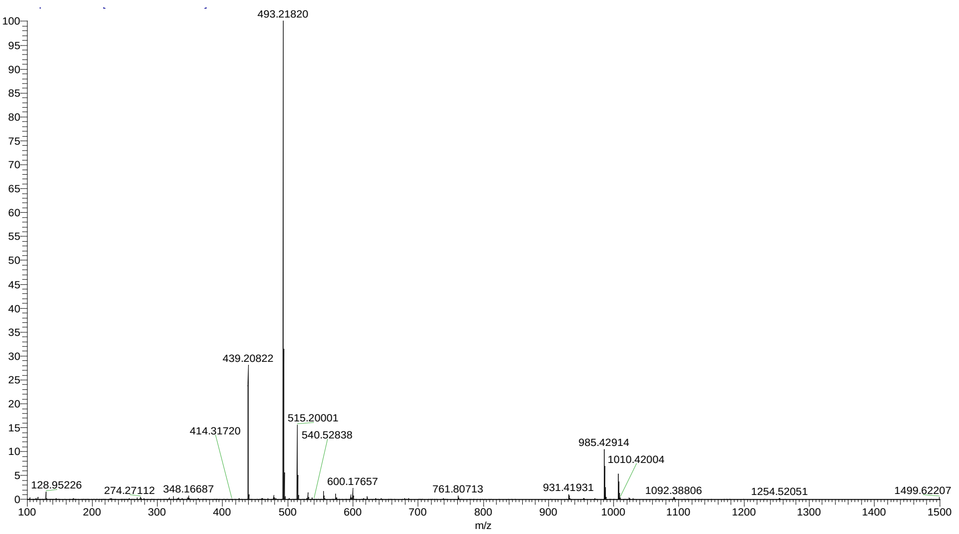

HRMS spectrum of P7

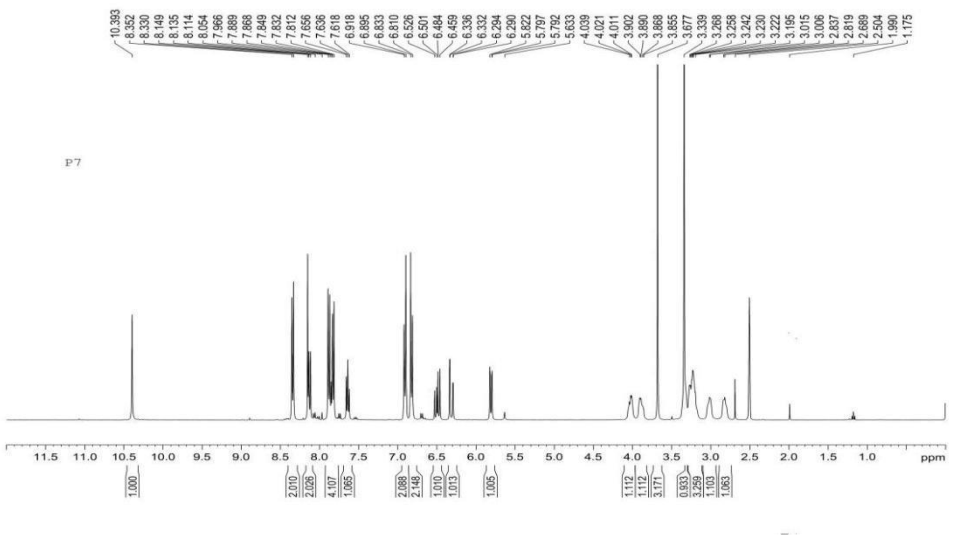

^1H NMR spectrum of P7

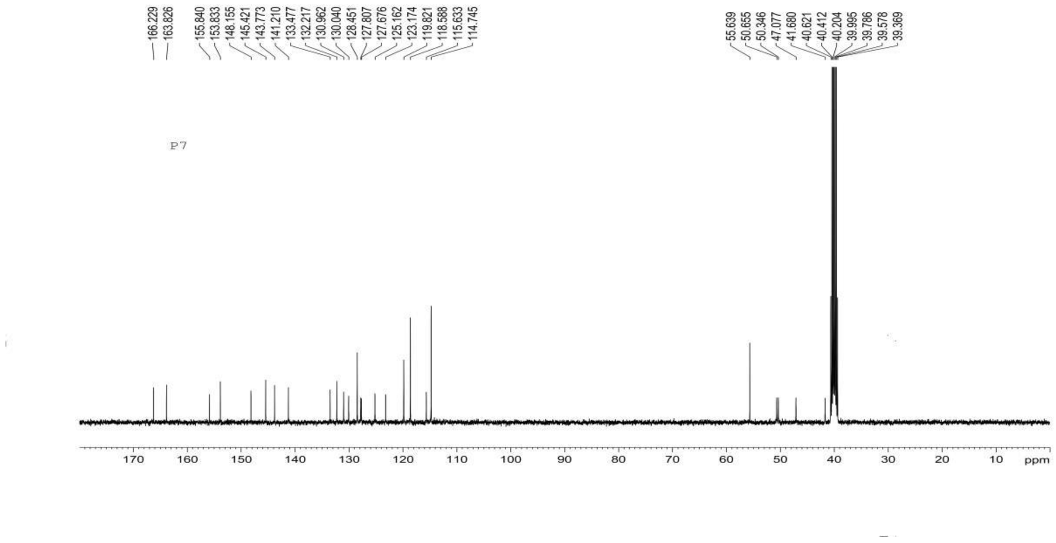

^{13}C NMR spectrum of P7

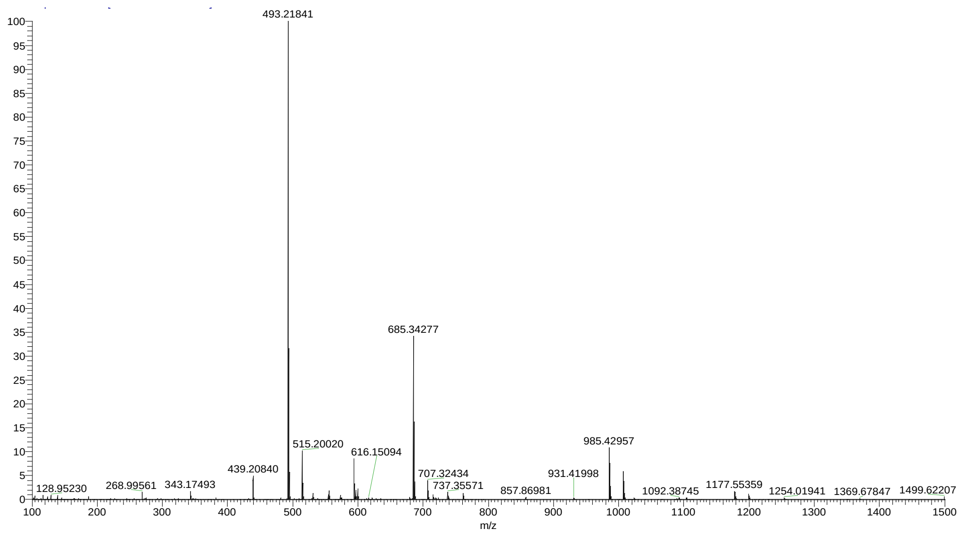

HRMS spectrum of P8

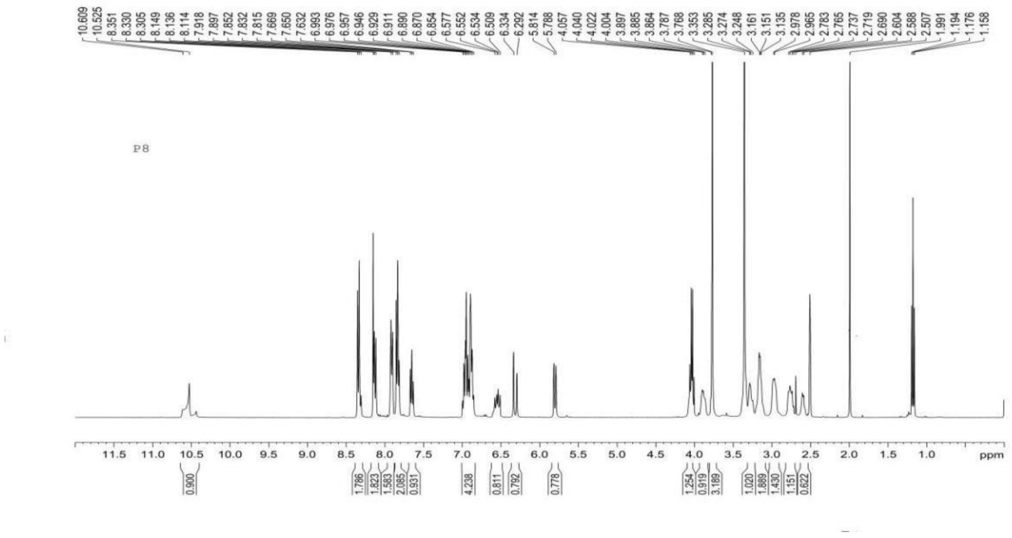

^{1}H NMR spectrum of P8

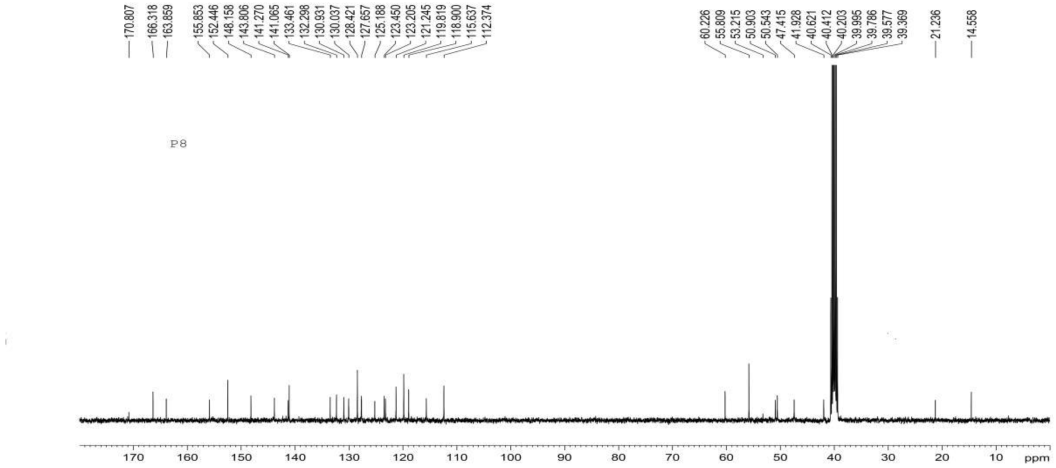

^{13}C NMR spectrum of P8

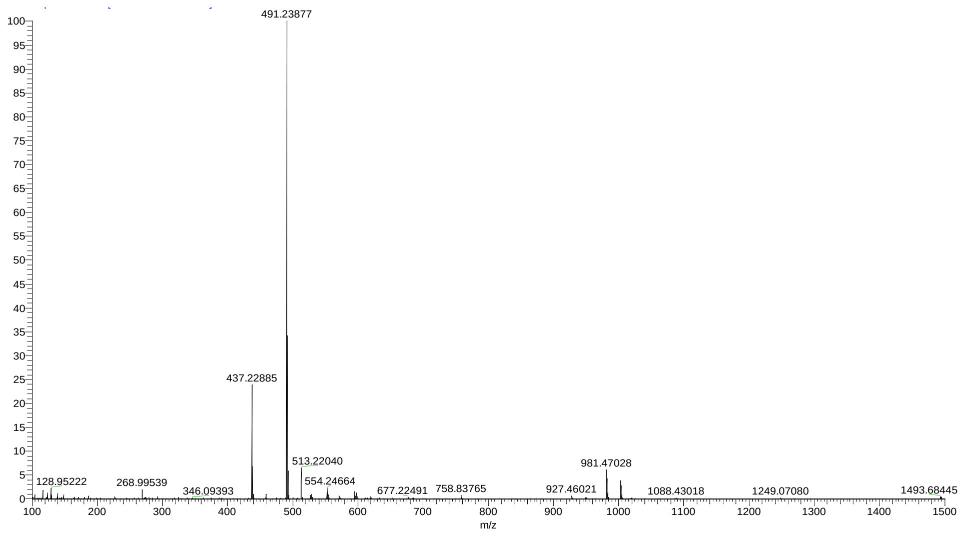

HRMS spectrum of P9

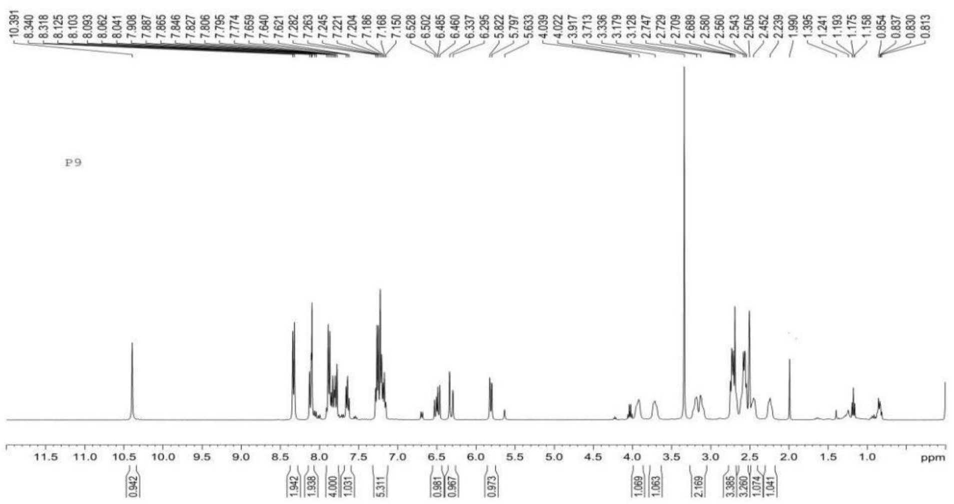

^{1}H NMR spectrum of P9

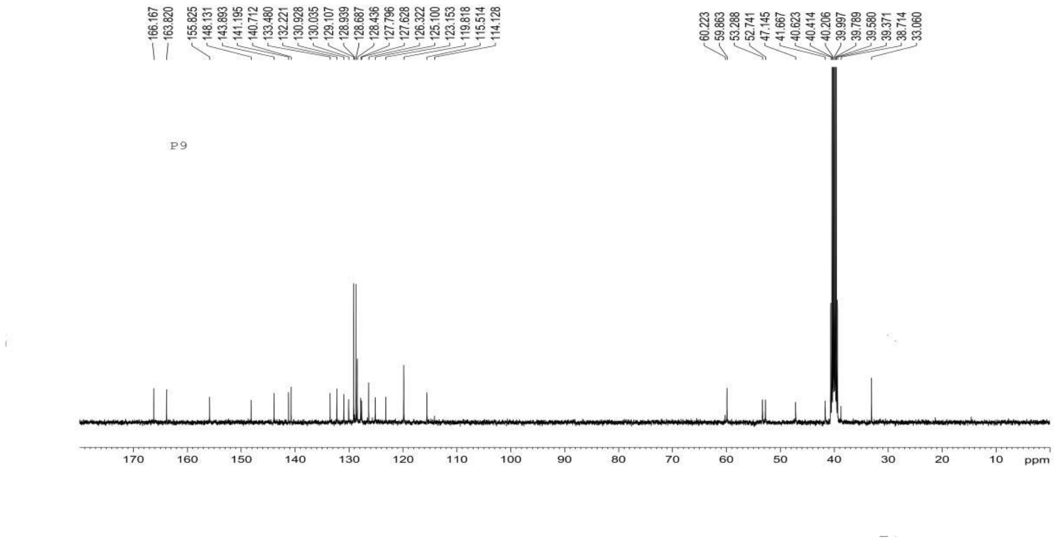

^{13}C NMR spectrum of P9

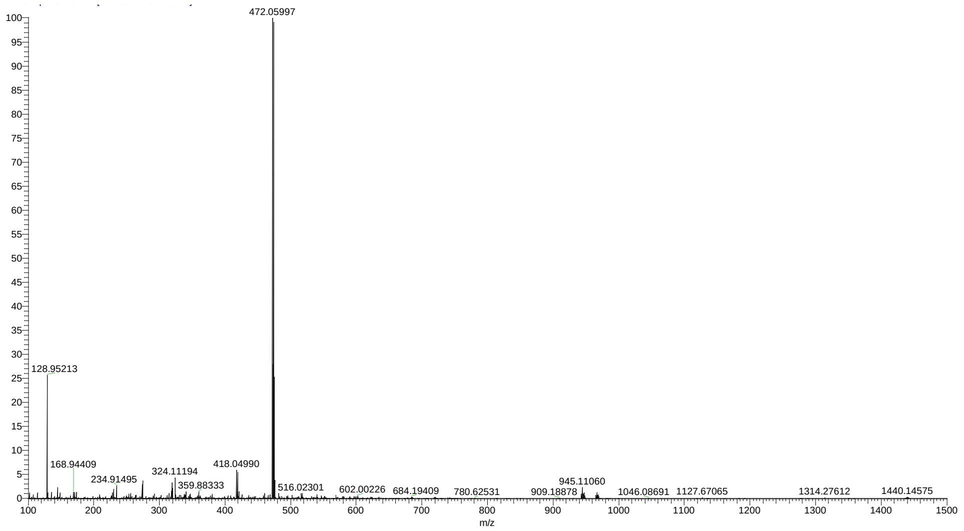

HRMS spectrum of P10

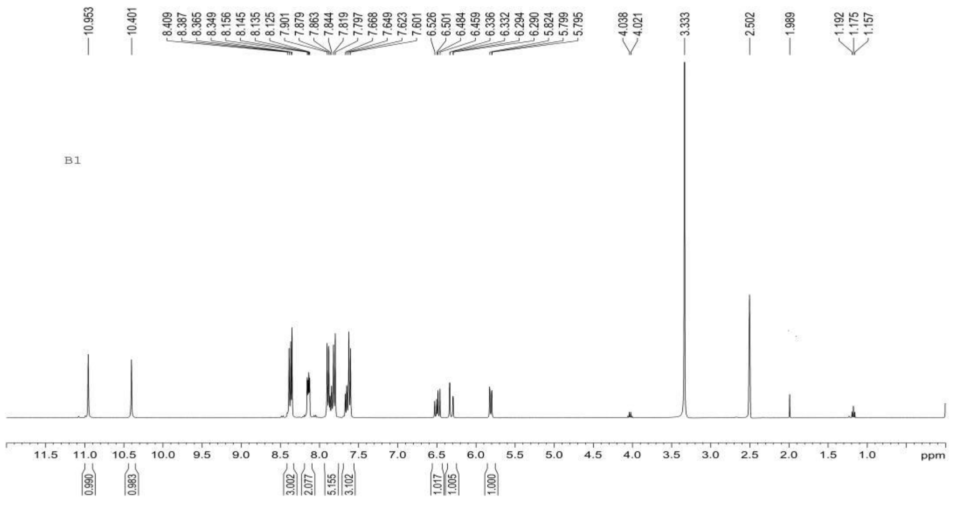

^{1}H NMR spectrum of P10

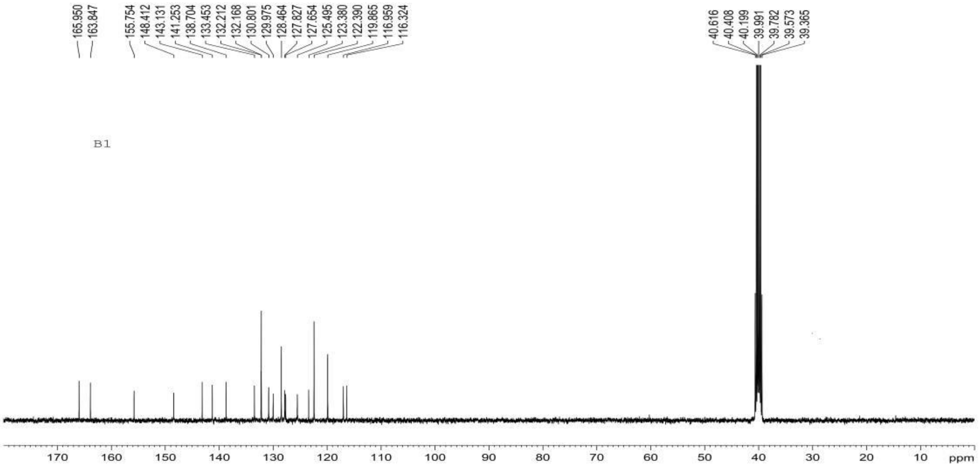

^{13}C NMR spectrum of P10

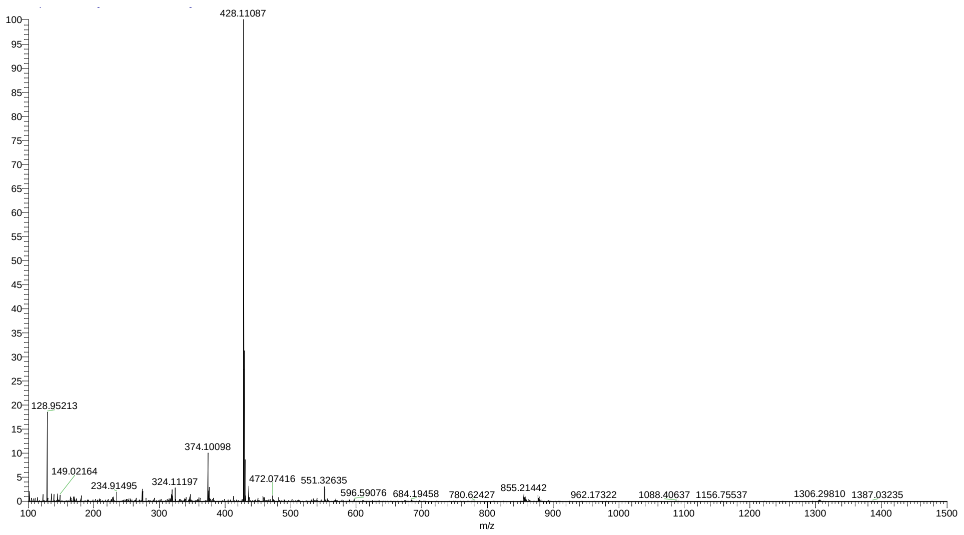

HRMS spectrum of P11

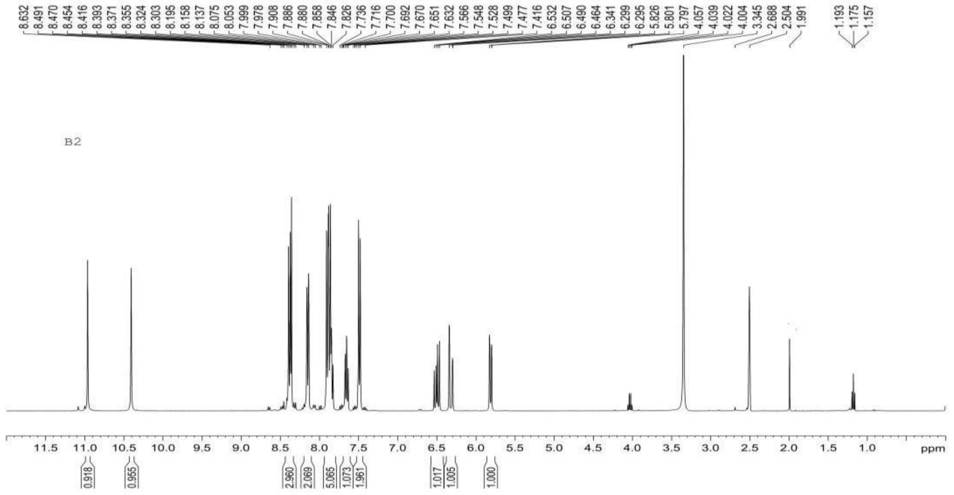

^{1}H NMR spectrum of P11

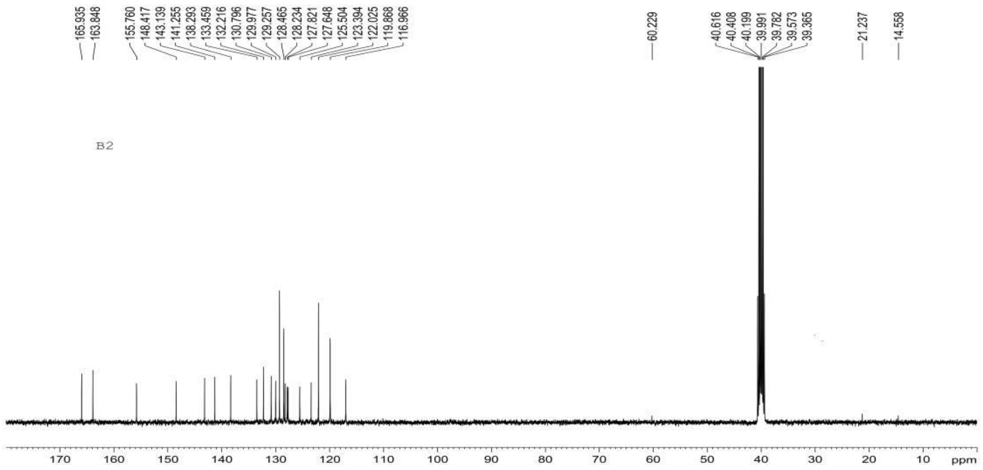

^{13}C NMR spectrum of P11

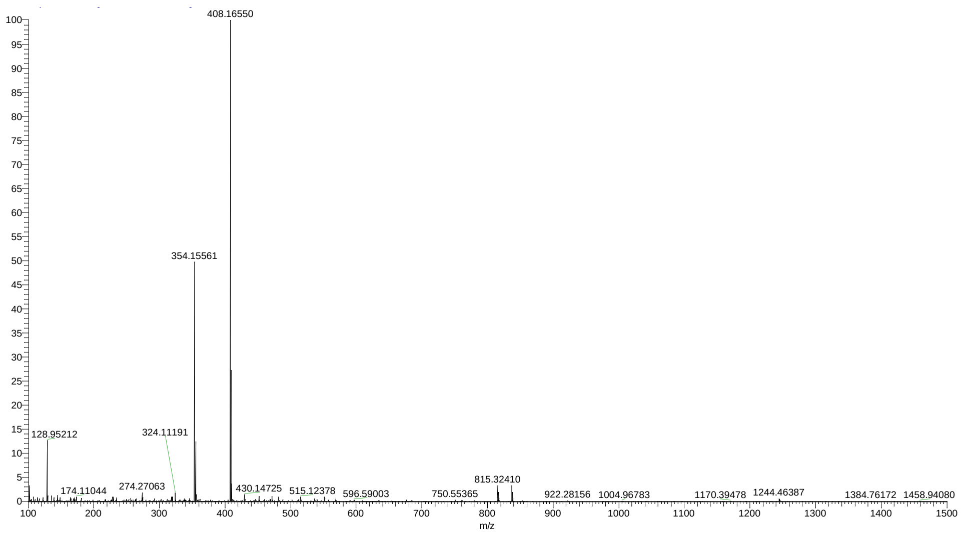

HRMS spectrum of P12

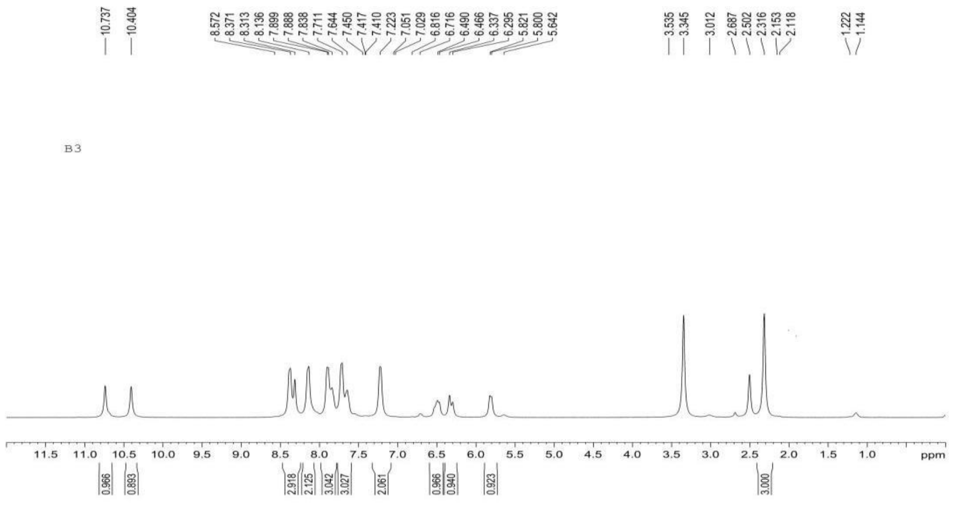

^{1}H NMR spectrum of P12

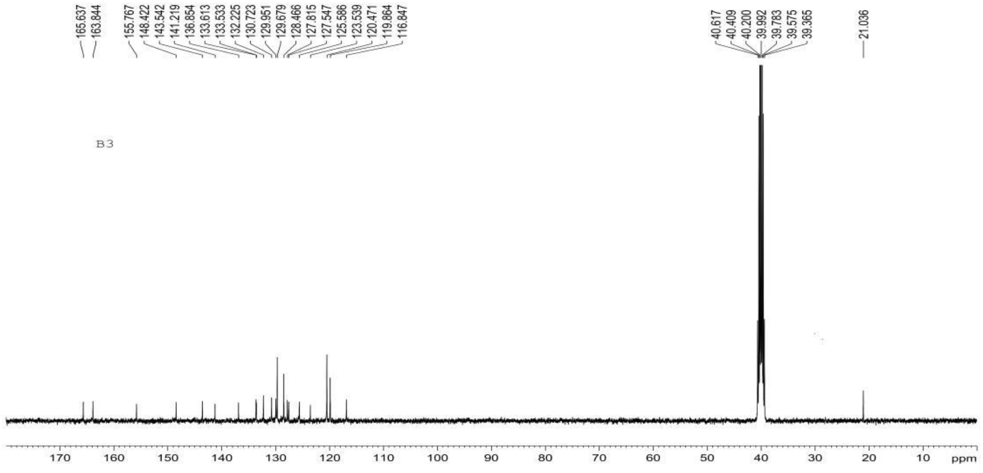

^{13}C NMR spectrum of P12

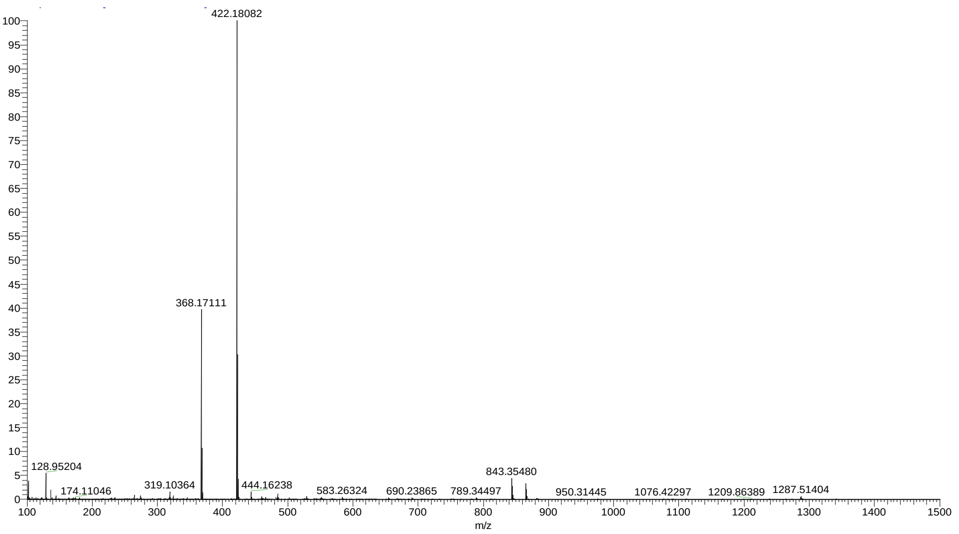

HRMS spectrum of P13

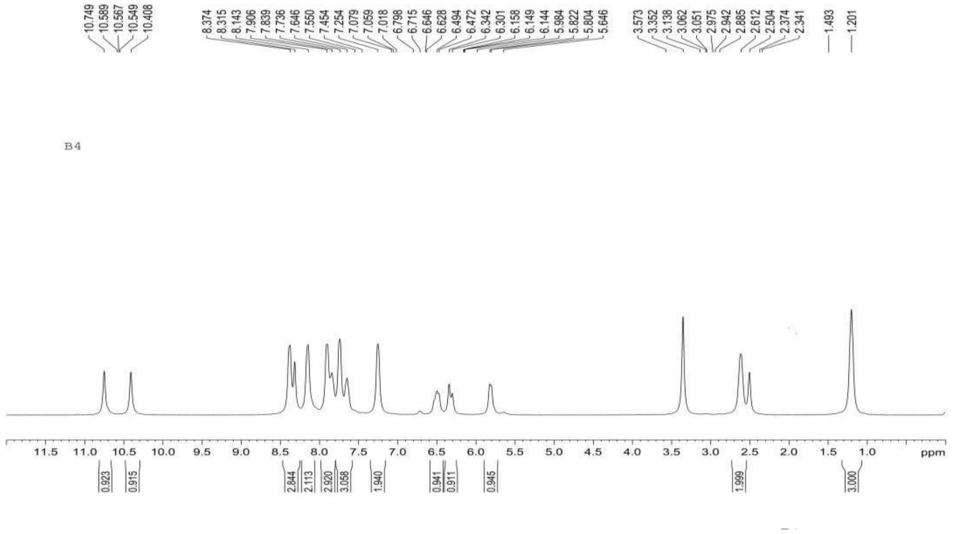

^1H NMR spectrum of P13

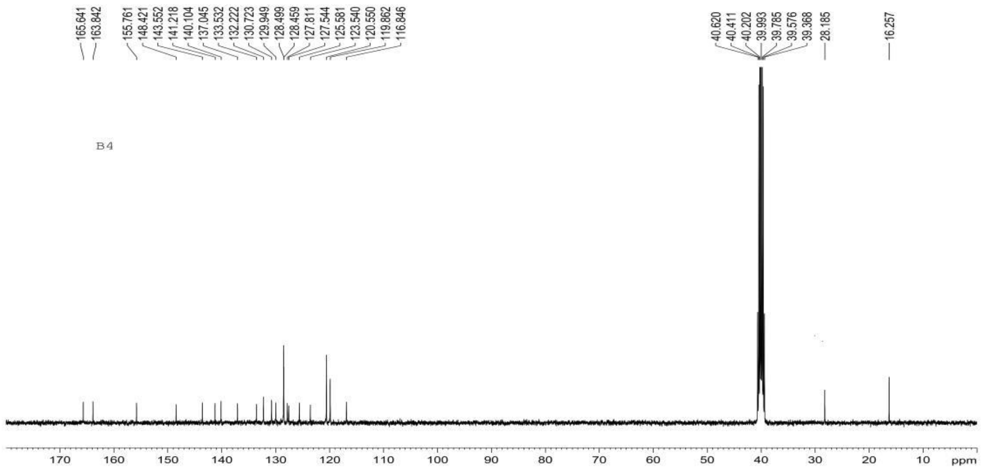

^{13}C NMR spectrum of P13

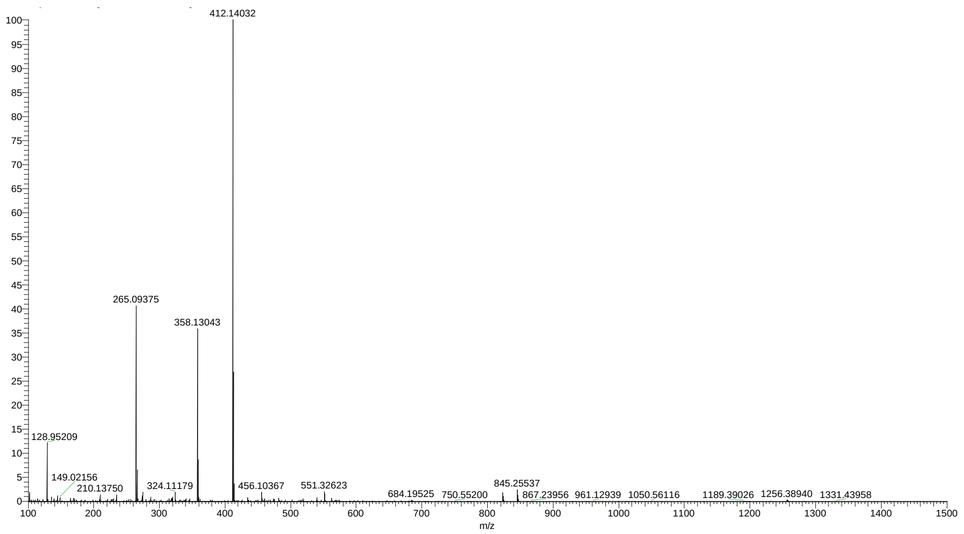

HRMS spectrum of P14

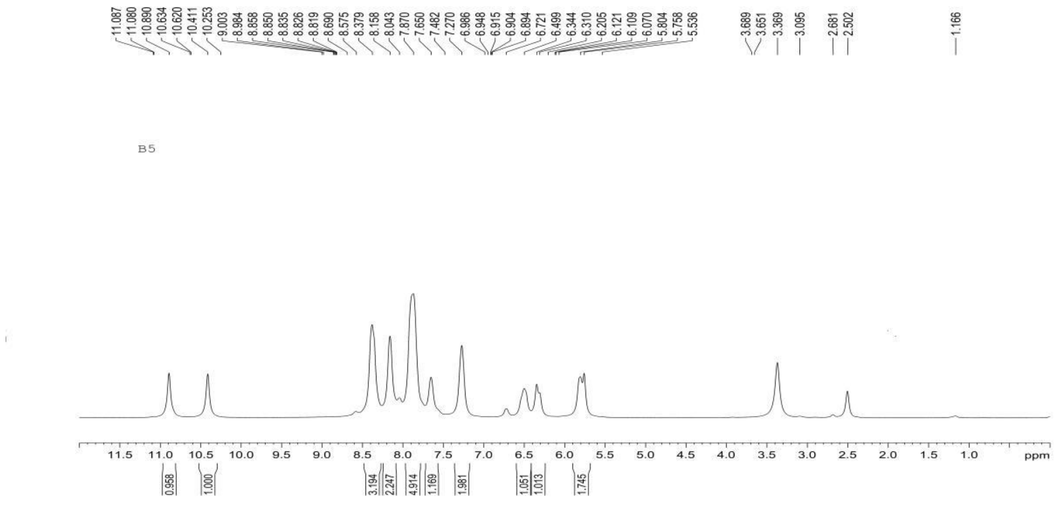

^{1}H NMR spectrum of P14

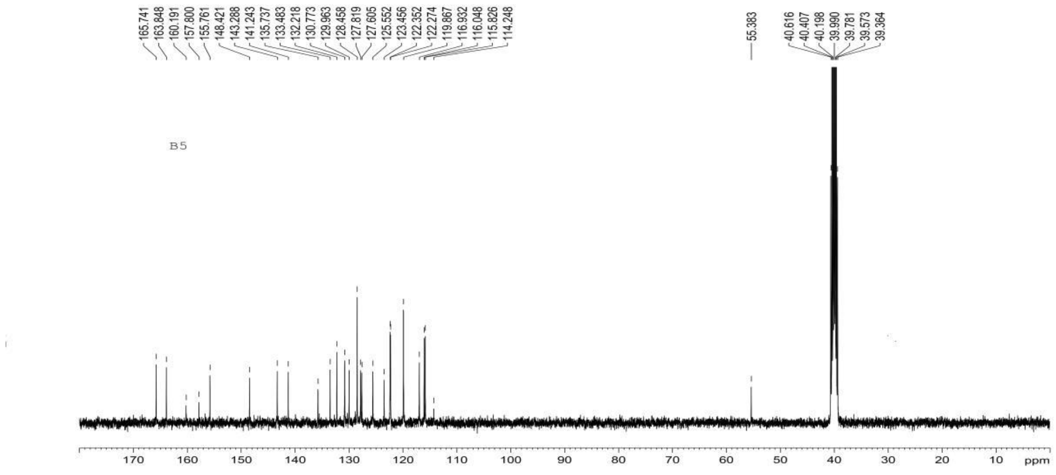

^{13}C NMR spectrum of P14

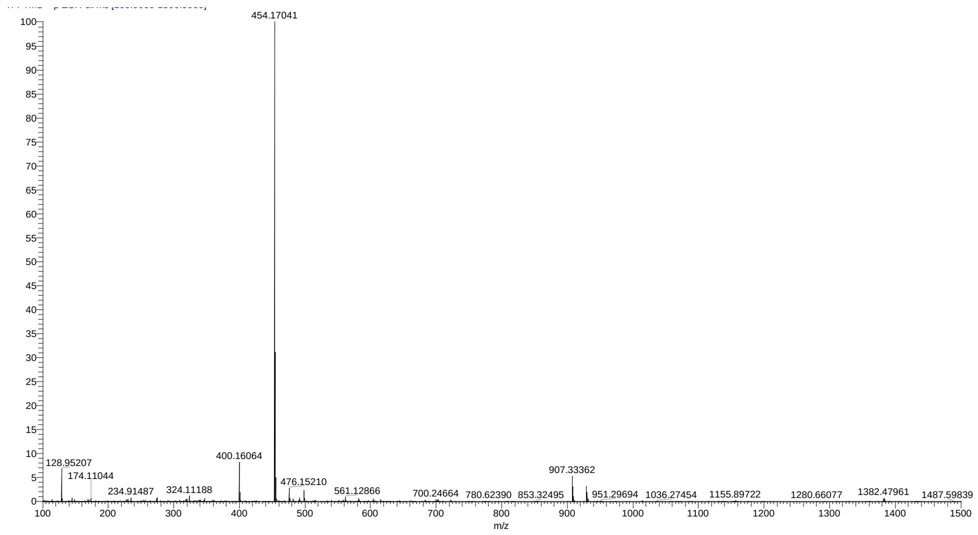

HRMS spectrum of P15

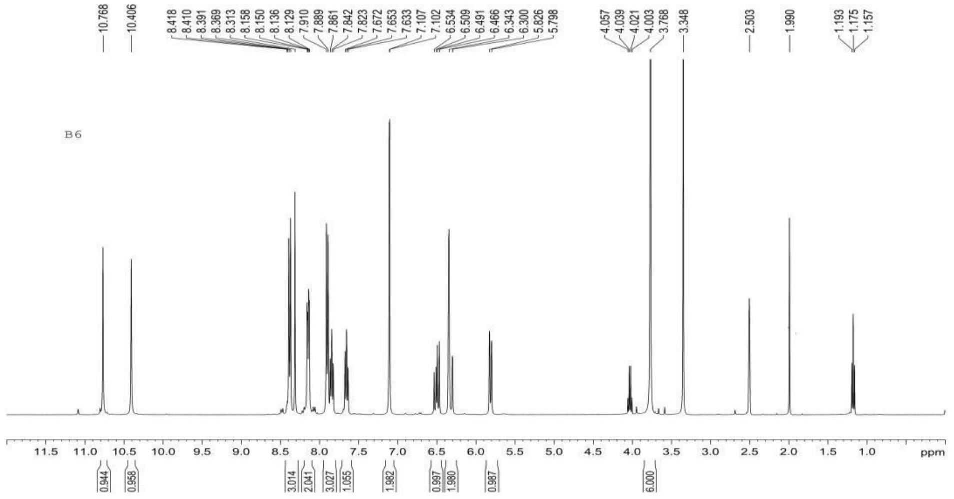

^{1}H NMR spectrum of P15

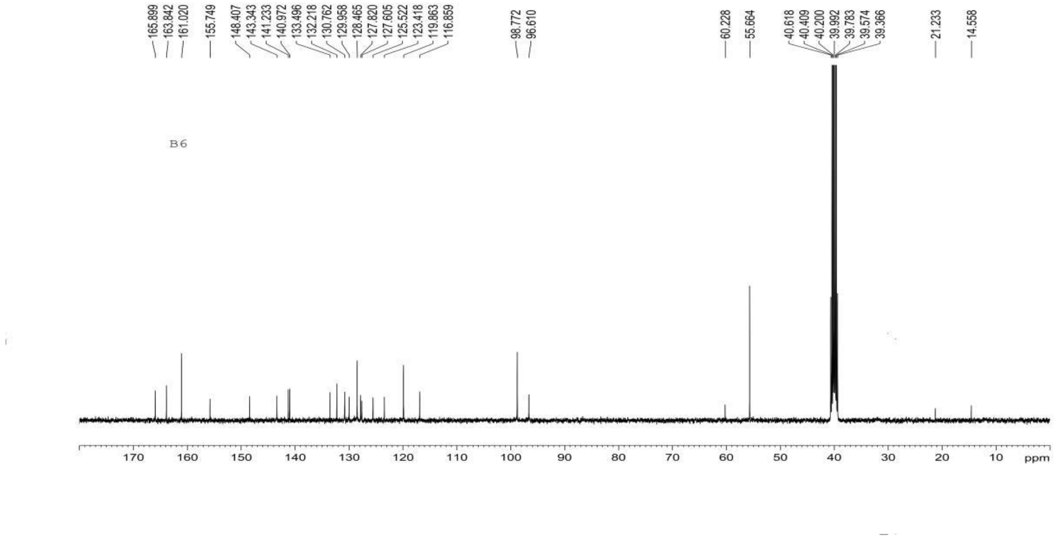

^{13}C NMR spectrum of P15

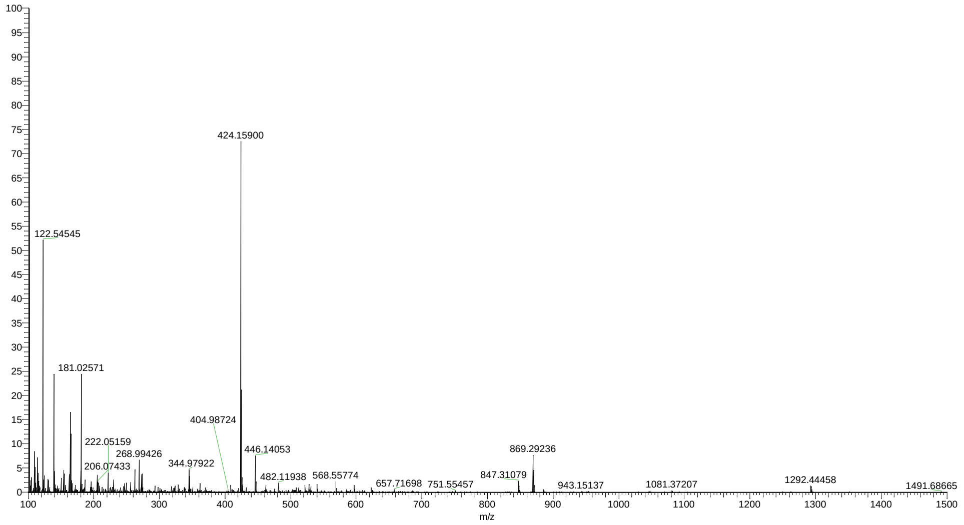

HRMS spectrum of P16

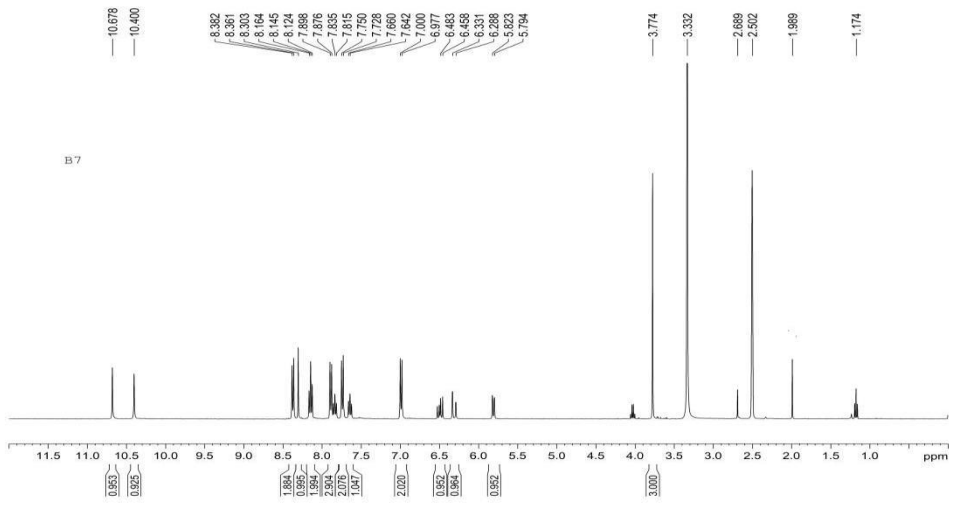

^{1}H NMR spectrum of P16

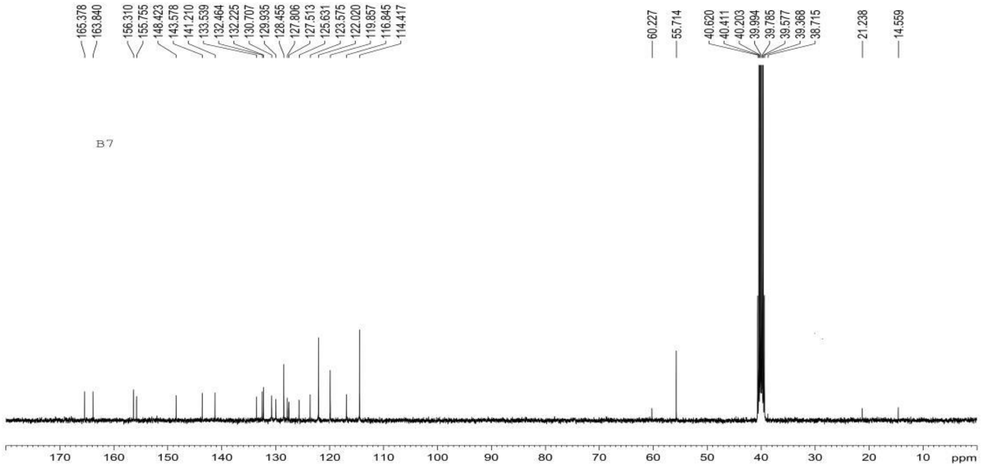

^{13}C NMR spectrum of P16

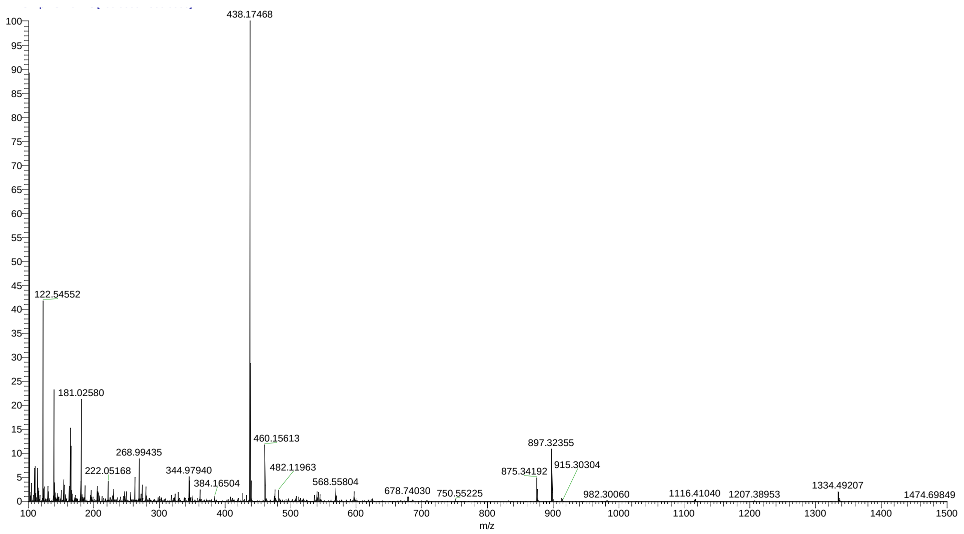

HRMS spectrum of P17

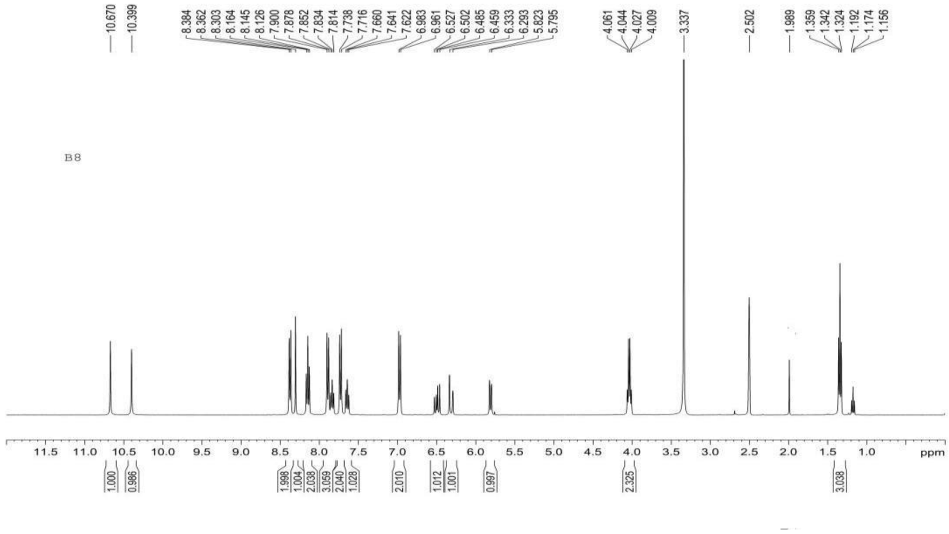

^{1}H NMR spectrum of P17

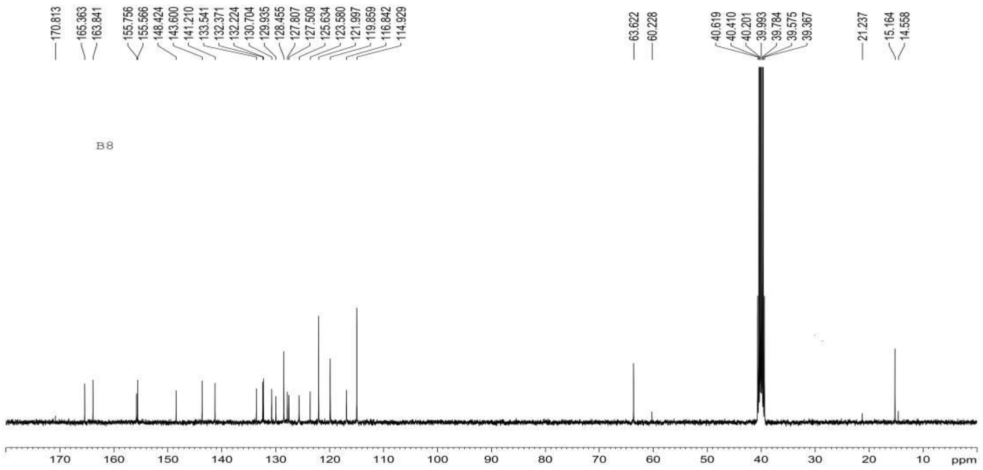

^{13}C NMR spectrum of P17

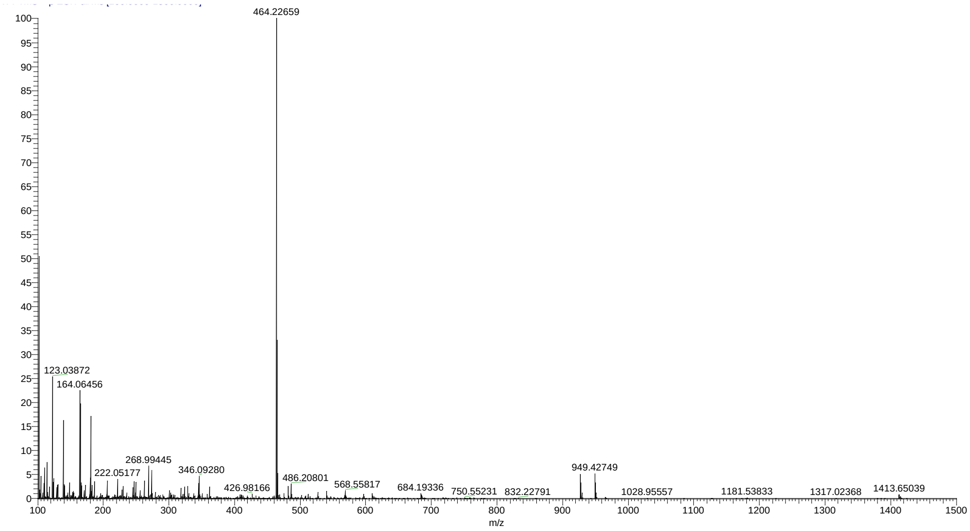

HRMS spectrum of P18

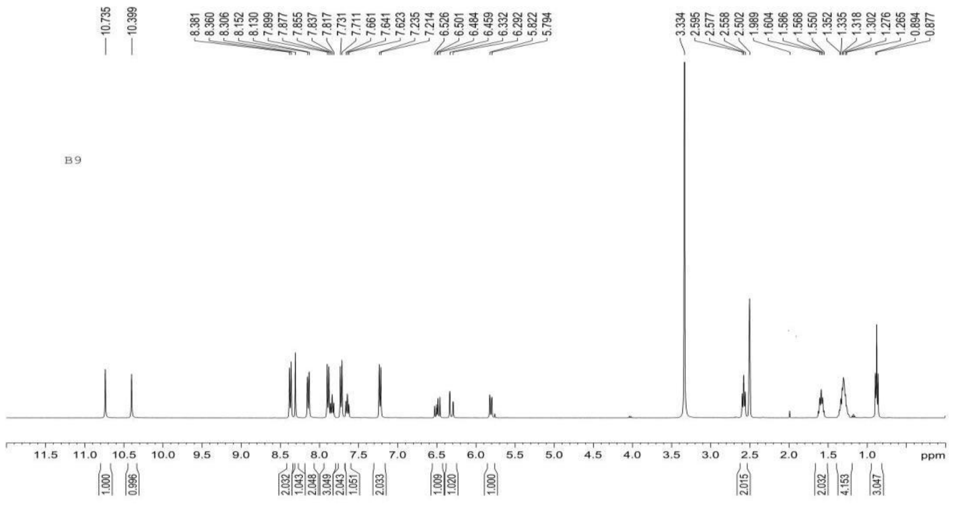

^{1}H NMR spectrum of P18

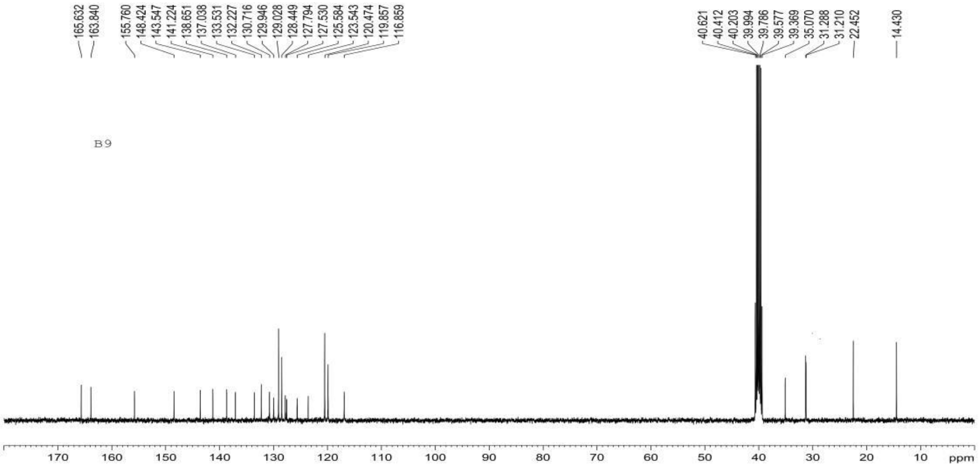

^{13}C NMR spectrum of P18

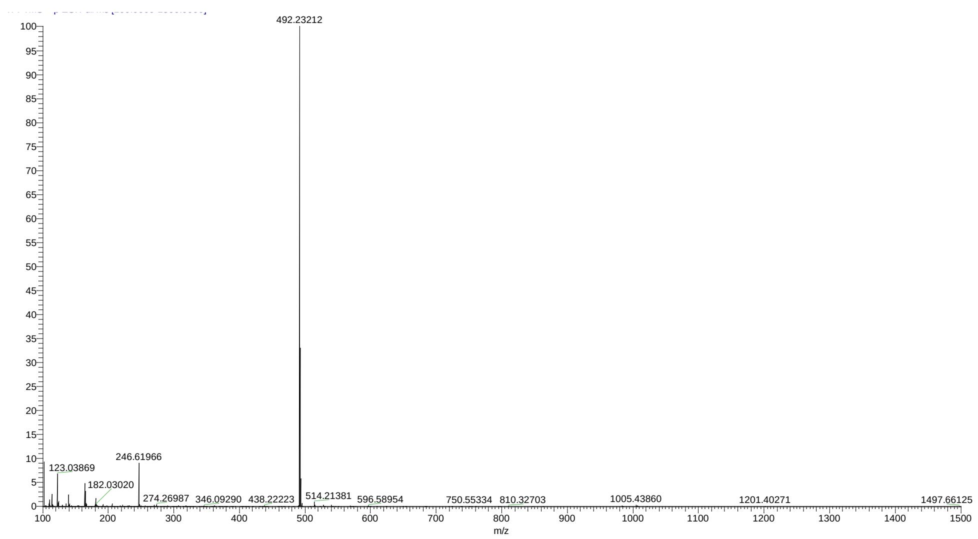

HRMS spectrum of P19

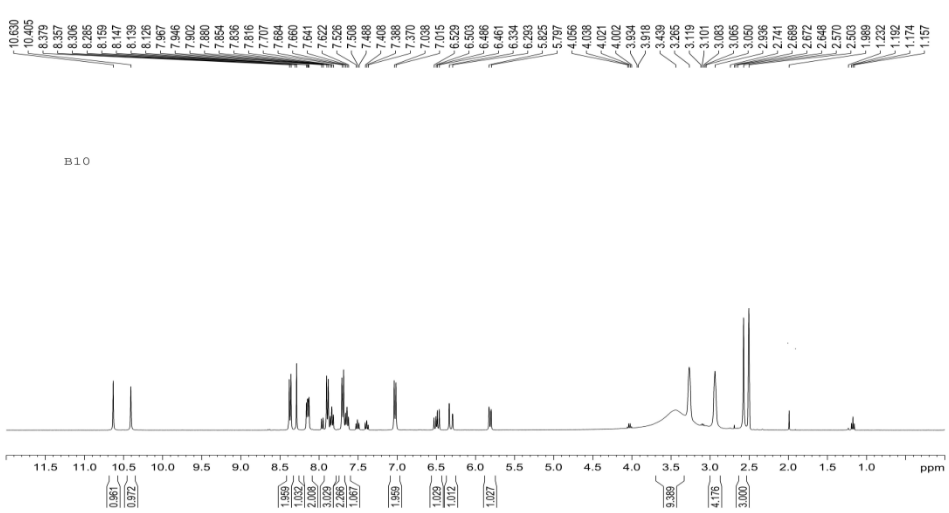

^{1}H NMR spectrum of P19

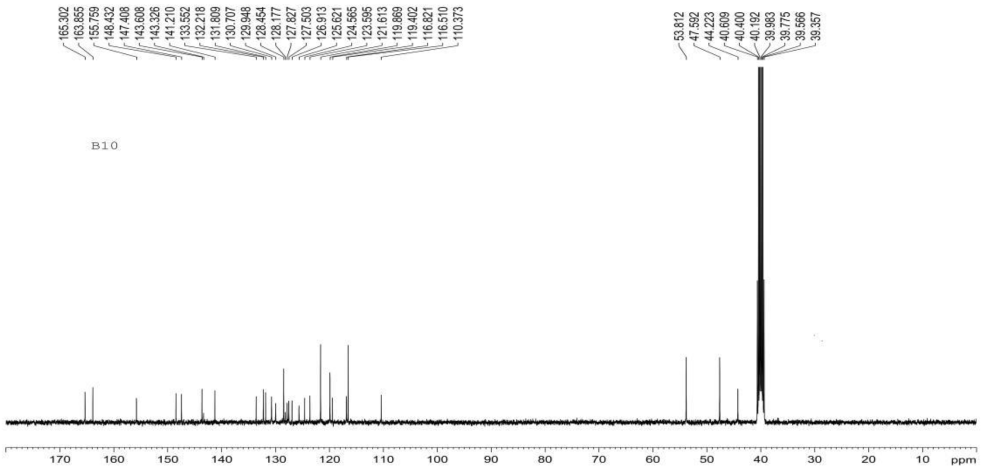

^{13}C NMR spectrum of P19

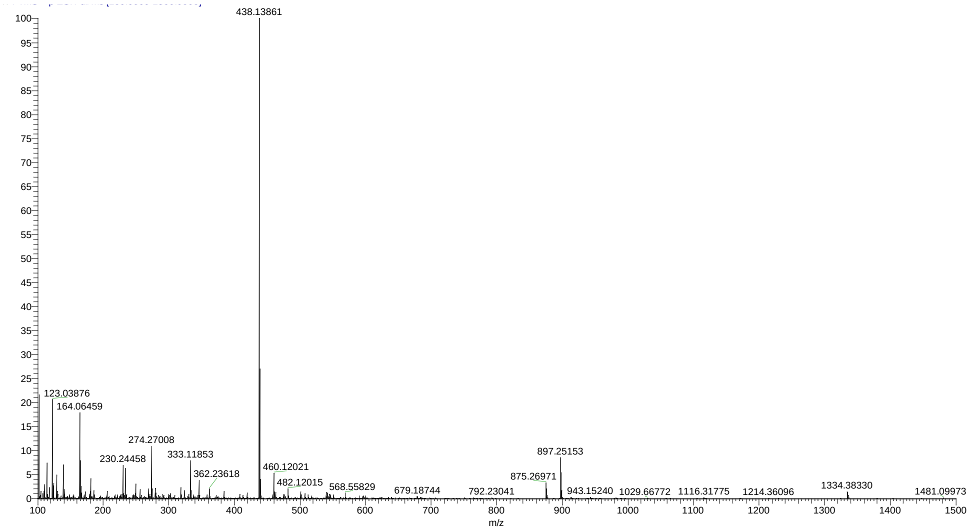

HRMS spectrum of P20

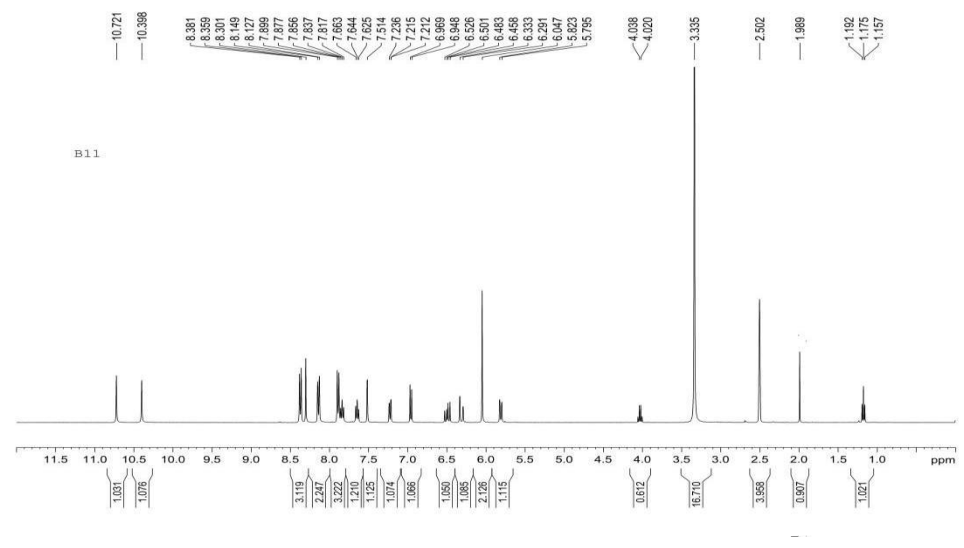

^{1}H NMR spectrum of P20

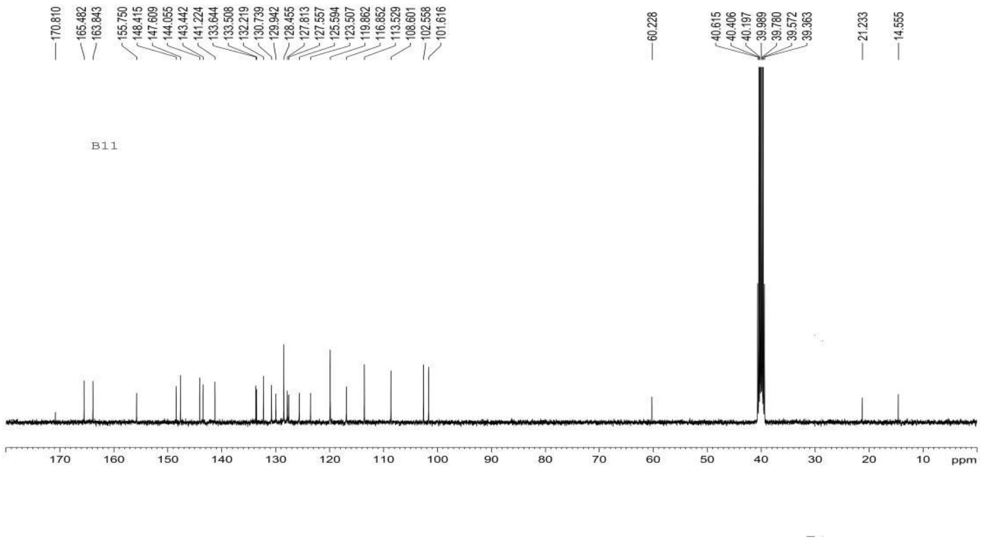

^{13}C NMR spectrum of P20

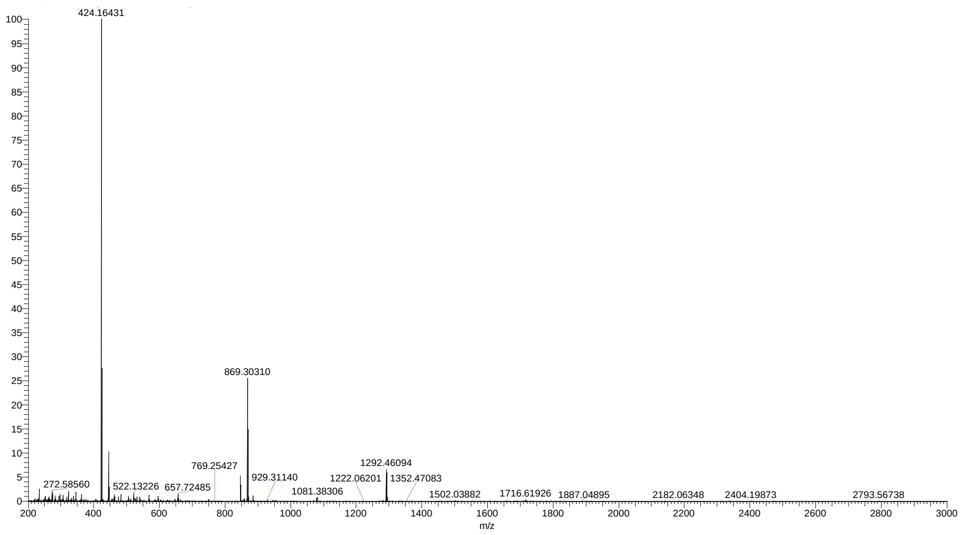

HRMS spectrum of P21

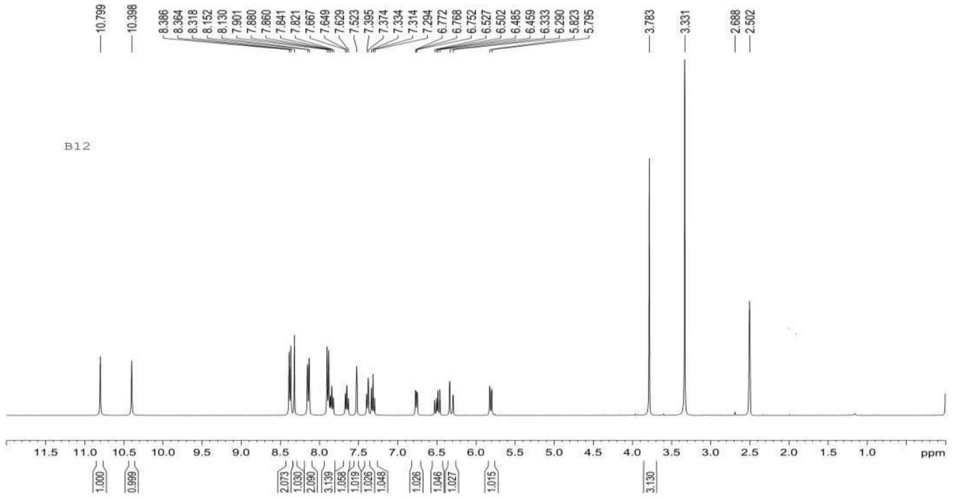

^{1}H NMR spectrum of P21

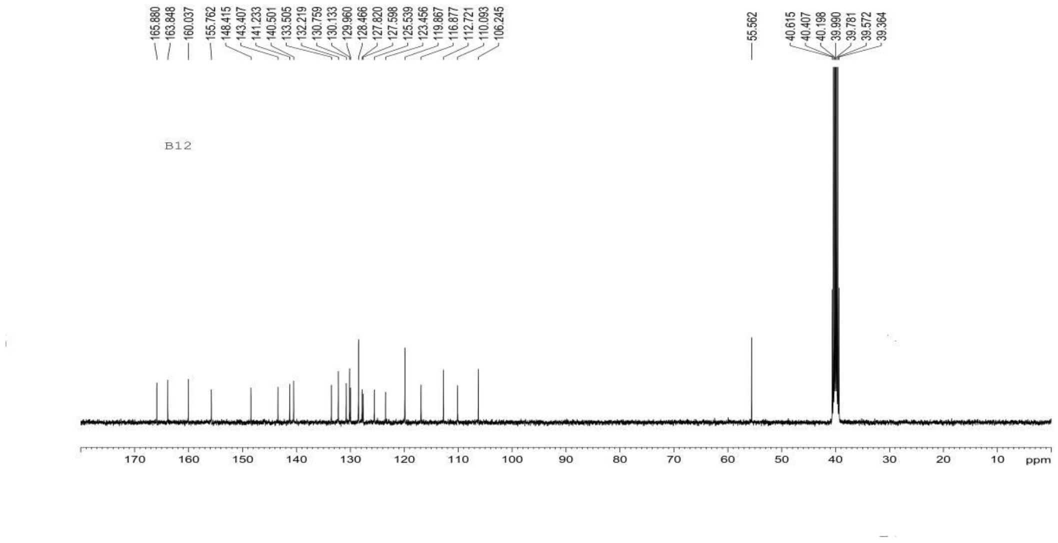

^{13}C NMR spectrum of P21

SIRT1 enzyme activity of Compound P6

P6	SIRT 1		% Activity	
	Repeat1	Repeat2	Repeat1	Repeat2
No compound	16663	16906		
**10 nM**	16532	17085	98	102
**33 nM**	16355	16465	97	98
**100 nM**	16823	16337	100	97
**333 nM**	16986	16012	101	95
**1 µM**	16237	15035	97	89
**3.3 µM**	15407	14308	91	85
**10 µM**	10593	11037	61	64
**33 µM**	7536	7093	42	40
**100 µM**	4670	4931	25	26
Background	721	745		

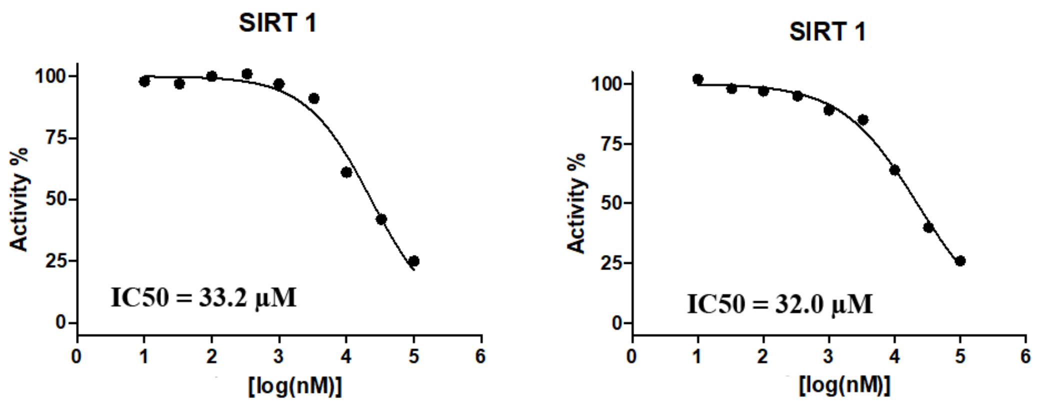

SIRT1 enzyme activity of Compound P19

P19	SIRT 1		% Activity	
	Repeat1	Repeat2	Repeat1	Repeat2
No compound	16663	16906		
**10 nM**	16431	16926	98	101
**33 nM**	16537	16148	98	96
**100 nM**	16153	16820	96	100
**333 nM**	17070	16959	102	101
**1 µM**	16383	17027	97	102
**3.3 µM**	16853	16786	100	100
**10 µM**	15747	15393	94	91
**33 µM**	11629	13000	68	76
**100 µM**	4974	5757	26	31
Background	721	745		

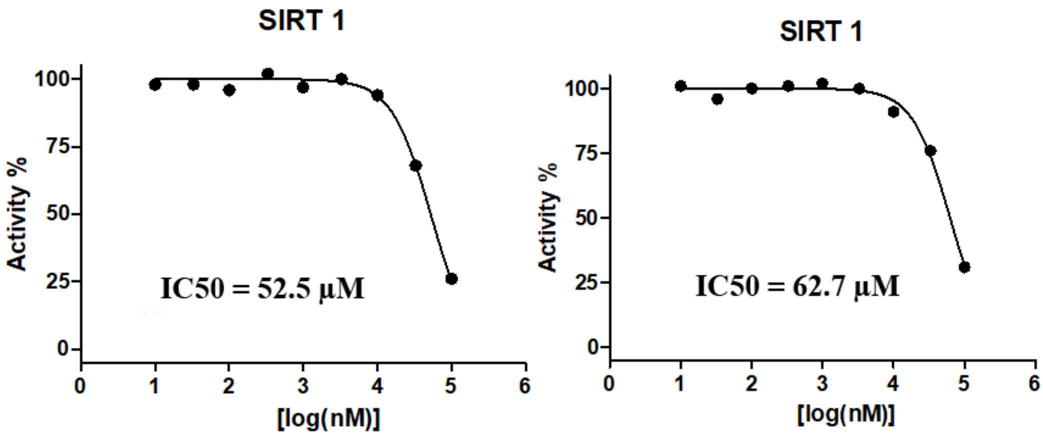

SIRT1 enzyme activity of Nicotinamide

Nicotinamide	SIRT 1		% Activity	
	Repeat1	Repeat2	Repeat1	Repeat2
No compound	16663	16906		
**10 nM**	17068	16526	102	98
**33 nM**	16978	16648	101	99
**100 nM**	16410	17074	98	102
**333 nM**	16568	16237	99	97
**1 µM**	16733	17029	100	102
**3.3 µM**	15607	16095	93	96
**10 µM**	13377	12494	79	73
**33 µM**	10165	9000	59	52
**100 µM**	2396	3386	10	17
Background	721	745		

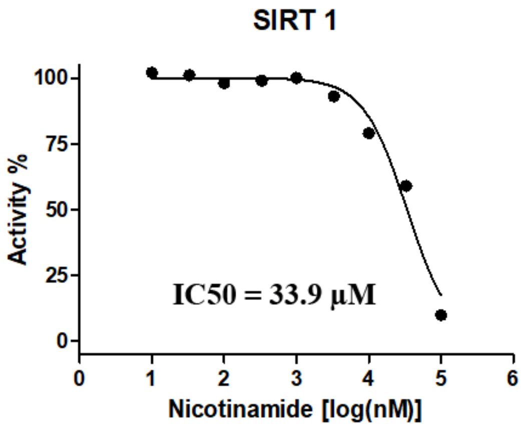

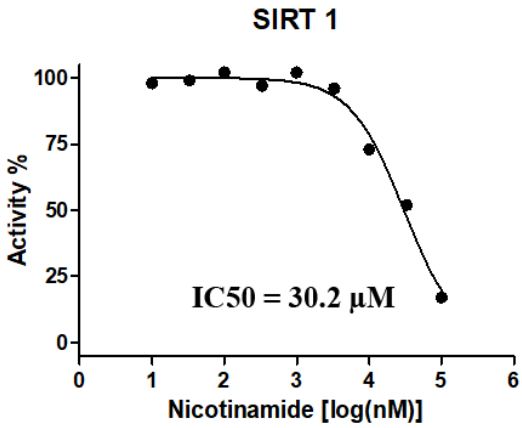

SIRT2 enzyme activity of Compound P6

P6	SIRT 2		% Activity	
	Repeat1	Repeat2	Repeat1	Repeat2
No compound	13177	13482		
**10 nM**	13270	13061	100	98
**33 nM**	12808	13329	96	100
**100 nM**	13512	13423	101	101
**333 nM**	13373	12690	100	95
**1 µM**	12498	11131	93	83
**3.3 µM**	11202	9952	83	73
**10 µM**	8767	8883	64	65
**33 µM**	7176	6799	51	48
**100 µM**	5223	5614	36	39
Background	635	664		

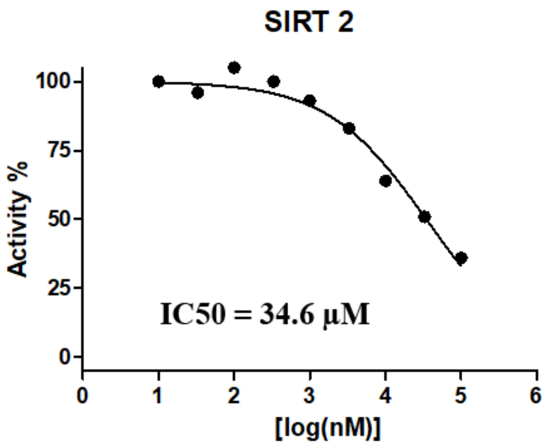

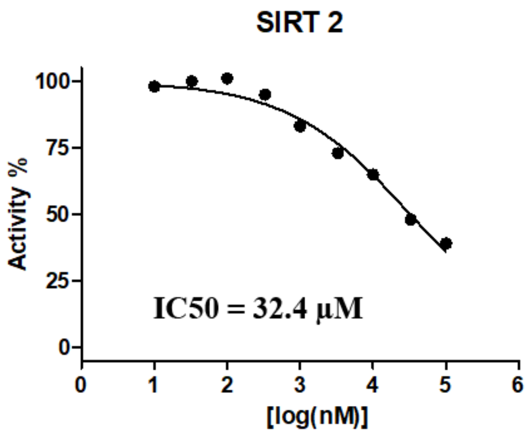

SIRT2 enzyme activity of Compound P19

P19	SIRT 2		% Activity	
	Repeat1	Repeat2	Repeat1	Repeat2
No compound	13177	13482		
**10 nM**	13481	13205	101	99
**33 nM**	13165	13149	99	99
**100 nM**	13507	13481	101	101
**333 nM**	12858	13040	96	98
**1 µM**	12952	13047	97	98
**3.3 µM**	13352	13247	100	99
**10 µM**	12220	11568	91	86
**33 µM**	10679	10301	79	76
**100 µM**	5005	4230	34	28
Background	635	664		

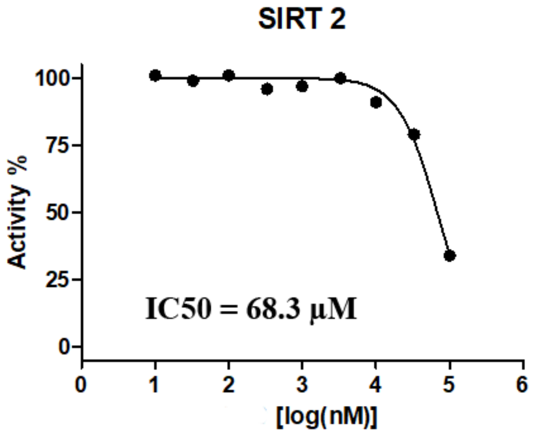

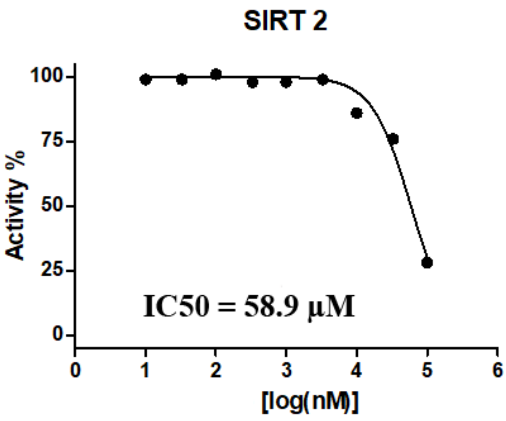

SIRT2 enzyme activity of Nicotinamide

Nicotinamide	SIRT 2		% Activity	
	Repeat1	Repeat2	Repeat1	Repeat2
No compound	13177	13482		
**10 nM**	13314	13195	100	99
**33 nM**	13247	13016	99	98
**100 nM**	12949	13411	97	101
**333 nM**	13303	13362	100	100
**1 µM**	13543	13210	102	99
**3.3 µM**	12313	12179	92	91
**10 µM**	10727	9353	79	69
**33 µM**	5581	6121	39	43
**100 µM**	2851	2124	17	12
Background	635	664		

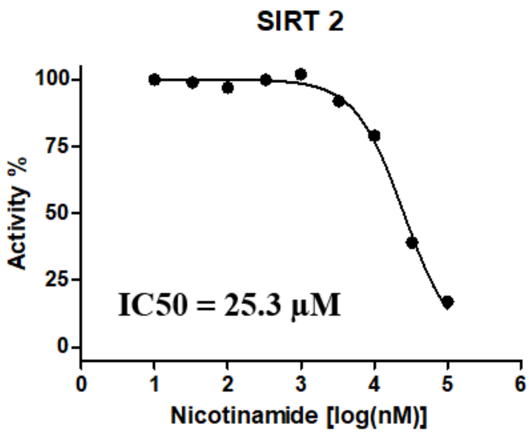

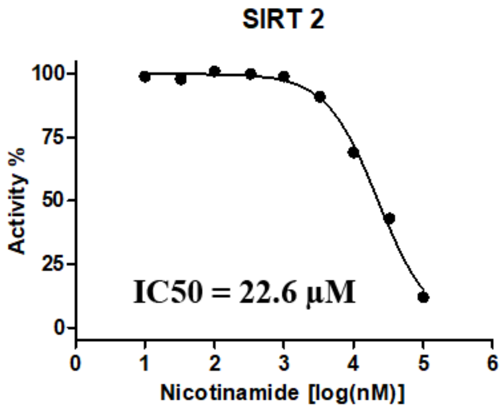

SIRT3 enzyme activity of Compound P6

P6	SIRT 3		% Activity	
	Repeat1	Repeat2	Repeat1	Repeat2
No compound	11395	11604		
**10 nM**	11621	11376	101	99
**33 nM**	11235	11165	98	97
**100 nM**	11933	10990	104	95
**333 nM**	10934	10166	95	88
**1 µM**	8868	8692	76	74
**3.3 µM**	7206	7060	61	59
**10 µM**	4731	4722	38	38
**33 µM**	4214	3899	33	31
**100 µM**	3175	2851	24	21
Background	581	537		

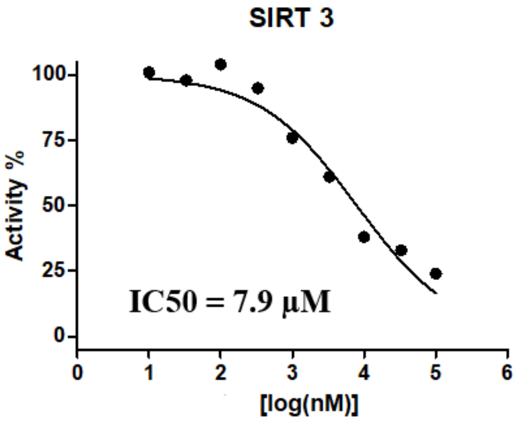

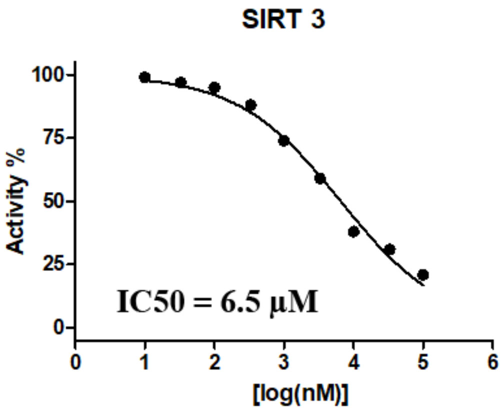

SIRT3 enzyme activity of Compound P19

P19	SIRT 3		% Activity	
	Repeat1	Repeat2	Repeat1	Repeat2
No compound	11395	11604		
**10 nM**	11533	11672	100	102
**33 nM**	11455	11457	100	100
**100 nM**	11362	11287	99	98
**333 nM**	11403	11158	99	97
**1 µM**	10799	10526	94	91
**3.3 µM**	9982	9732	86	84
**10 µM**	7709	8164	65	70
**33 µM**	5310	5994	43	50
**100 µM**	3046	3713	23	29
Background	581	537		

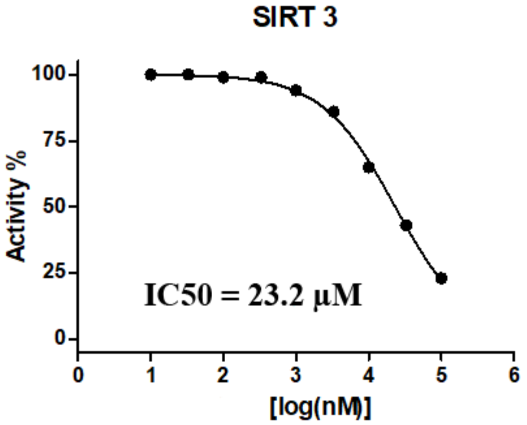

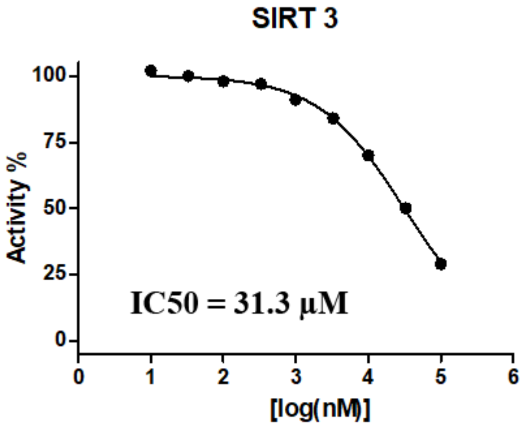

SIRT3 enzyme activity of Nicotinamide

Nicotinamide	SIRT 3		% Activity	
	Repeat1	Repeat2	Repeat1	Repeat2
No compound	11395	11604		
**10 nM**	11143	11688	97	102
**33 nM**	11094	11481	96	100
**100 nM**	11351	11076	99	96
**333 nM**	11611	11289	101	98
**1 µM**	10462	11364	91	99
**3.3 µM**	8995	9313	77	80
**10 µM**	7855	6777	67	57
**33 µM**	4610	3946	37	31
**100 µM**	1549	1945	9	13
Background	581	537		

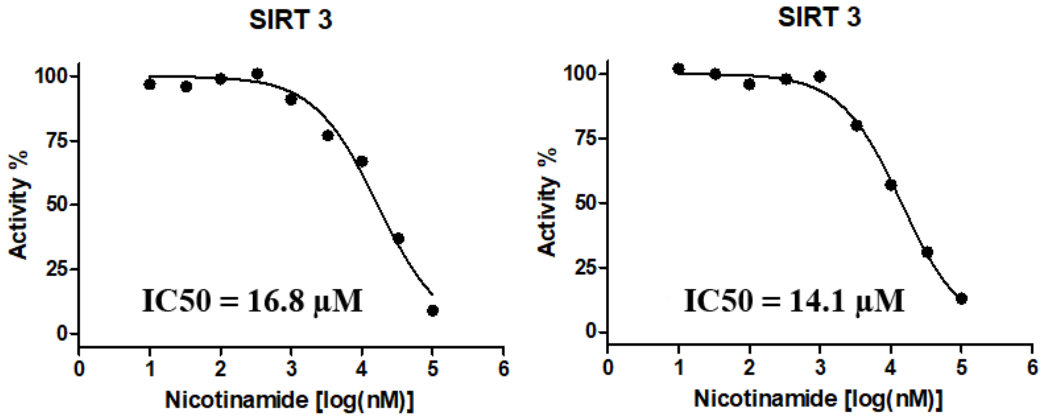
SIRT 3
Activity %
100
75
50
25
0
IC50 = 16.8 µM
0 1 2 3 4 5 6
Nicotinamide [log(nM)]
SIRT 3
Activity %
100
75
50
25
0
IC50 = 14.1 µM
0 1 2 3 4 5 6
Nicotinamide [log(nM)]
